# Supplementary material for: Heterogeneous impacts of HIV pre-exposure prophylaxis (PrEP) on drug resistance and phylogenetic cluster transmission dynamics in British Columbia, Canada: A retrospective cohort and simulation study
Source: PLoS Med. 2025 Dec 9;22(12):e1004827. doi: 10.1371/journal.pmed.1004827 (PMC12700434; doi:10.1371/journal.pmed.1004827)
Supplement: S1 Text — Table A. HIV-1 antiretroviral therapy (ART) drug names, abbreviation codes, and classes. Drug classes include nucleoside analogue reverse transcriptase inhibitor (NRTI); non-NRTI (NNRTI); protease inhibitor (PI); and integrase strand transfer inhibitor (INSTI). Excludes broadly neutralizing antibodies, capsid inhibitors, and other classes). Components of TDF/FTC oral PrEP are marked in bold. Table B. First and most recent drug regimens of newly diagnosed PrEP users. Table C. Characteristics of newly diagnosed PrEP users and non-PrEP users from 2018 to 2022. Proportions compared using chi-squared test and medians compared by Kruskal–Wallis tests. Table D. Sociodemographic and clinical factors associated with clustering among newly diagnosed PrEP users and non-PrEP users from 2018 to 2022. P-values reported for two-sided chi-squared tests for categorical variables and Kruskal–Wallis tests for numeric variables. Fig A. New diagnoses in active HIV phylogenetic clusters in BC since 2018. A) Total new diagnoses in active clusters, annotated with cluster ID; cluster size at the end of study period in December 2022. Bars are colored by PrEP use. B) Monthly new diagnoses that clustered during the study period, colored by PrEP use. C) New diagnoses in the study period by cluster. Annotated with cluster ID for the eight largest clusters. Fig B. Clustering and lineage-level viral diversification rate among newly diagnosed with and without PrEP use. A) Comparison of the proportion of individuals who clustered (membership, yes or no) by PrEP use, using a two-sided chi-squared test. B) Distribution of viral lineage-level diversification rate among newly diagnosed PrEP users and non-PrEP users, compared using a Kruskal–Wallis test. C) Viral lineage-level diversification rates were compared by PrEP use and clustering using pairwise Mann–Whitney tests. Fig C. Distribution of phylogenetic cluster sizes identified using multiple rooting strategies. Trees were rooted using midpoint (Fas [file pmed.1004827.s001.pdf]

Supplementary materials

Heterogeneous impacts of HIV pre-exposure prophylaxis (PrEP) on drug resistance and phylogenetic cluster transmission dynamics in British Columbia, Canada: a retrospective cohort and simulation study

Angela McLaughlin[1,2], Junine Toy[1], Vince Montoya[1], Paul Sereda[1], Jason Trigg[1], Mark Hull[1], Chanson J. Brumme[1,3], Rolando Barrios[1], Julio S.G. Montaner[1,3], Jeffrey B. Joy[1,2,3*]

[1] British Columbia Centre for Excellence in HIV/AIDS, Vancouver, Canada
[2] Bioinformatics, University of British Columbia, Vancouver, Canada
[3] Department of Medicine, University of British Columbia, Vancouver, Canada

* Corresponding author
Email: jjoy@bccfe.ca

Table of contents

Supplementary methods....1
- Sequence cleaning, alignment, and subtype assignment....1
- Pre-exposure prophylaxis (PrEP) eligibility and regimens in British Columbia (BC)....2
- PrEP dispensation patterns of newly diagnosed PrEP users....3
- ART regimens of newly diagnosed PrEP users....4
- Lineage-level viral diversification rate....5

Supplementary results....5
- Sociodemographic and clustering characteristics of newly HIV diagnosed with and without PrEP....5
- Phylogenetic clustering and diversification rates of newly diagnosed with and without PrEP....8
- Comparison of cluster identification under different tree rooting strategies....9
- Baseline drug resistance of newly diagnosed individuals with and without PrEP....10
- Drug resistance scores of newly diagnosed individuals with and without PrEP....11
- Active HIV-1 phylogenetic clusters in BC....12
- HIV effective reproduction number (R_e) in British Columbia and key populations....20
- Phylogenetic cluster-specific R_e....28
- Counterfactual simulations of cluster growth....34

References....41

Supplementary methods

Sequence cleaning, alignment, and subtype assignment

There were 42,043 HIV-1 partial *pol* (*protease* and partial *reverse transcriptase*) sequences from 10,740 drug treatment program (DTP) participants collected between May 30, 1996 and December 31, 2022. Sequences were removed if they had no patient identifier (n=17) or duplicate laboratory

identifier (n=101), leaving 41,941 *pol* sequences (1-50 sequences per patient). For 20 sequences, collection date was imputed as the test request date plus the median difference between collection date and request date for all other samples in that year. Data included 8759 *integrase* (*int*) sequences collected August 21, 1996 to December 31, 2022 representing 4058 unique patients. There were 309 *int* sequences without a collection date, for which we queried the sample collection date from the matching sample in the *pol* sequence; 20 sequences were removed because no matching sample date was available leaving 8739 sequences from 4052 patients (1-35 sequences per patient). Partial *pol* and *int* sequences were aligned to the HXB2 reference genome (GenBank Accession #K03455) using minimap2 parallelized by viralMSA [1,2]. COMET was used to assign HIV-1 subtypes for partial *pol* [3]. For COMET subtype assignments with less than 90% bootstrap support, subtype assignment was evaluated using REGA [4], and the assignment with higher support was chosen. 41,477 subtypes were assigned with COMET and 95 with REGA. Codons of surveillance drug resistance mutations (SDRMs) [5] and insertions relative to reference HXB2 were removed prior to phylogenetic inference.

Pre-exposure prophylaxis (PrEP) eligibility and regimens in British Columbia (BC)

As per guidance for the use of PrEP in BC updated in 2020, individuals must be at substantial risk of acquiring HIV and clinically eligible (HIV negative test within 15 days, no signs of HIV infection, normal renal function, and hepatitis B virus infection status) [6] For people who inject drugs (PWID), that constitutes reporting shared injection equipment with a HIV virally unsuppressed injecting partner. Heterosexual (HET) men and women are eligible if they report condomless intercourse with a virally unsuppressed partner. For gay, bisexual, and other men who have sex with men (GBM), eligibility is determined based on reporting condomless sex and a recent syphilis, gonorrhea, or chlamydia infection; an ongoing sexual relationship with an HIV-positive partner with detectable viral load; repeated courses of post-exposure prophylaxis; or a HIV Incidence Risk Index (HIRI) score greater than or equal to 10. A HIRI score is calculated on the basis of age, number of male sex partners, frequency of receptive anal intercourse, number of HIV positive male sex partners, frequency of unprotected anal sex with someone who is HIV positive, and recent use of methamphetamines [6].

Truvada (emtricitabine/tenofovir disoproxil fumarate; FTC/TDF) is an oral PrEP regimen recommended for daily use, but an on-demand dosing schedule may be considered for cis-gender GBM, following the “2-1-1” dosage with 2 pills 2-24 hr before sex and 1 pill daily until 48 hours after sex [7]. Individuals prescribed PrEP are required to return for follow-up visits after 1 month and at minimum every 3 months for HIV and sexually transmitted infection (STI) testing, renal function analysis, adherence counselling, risk reduction support, side effect assessment, and STI symptom assessment. In this study, we did not differentiate between daily versus on-demand PrEP usage. Other PrEP delivery systems including oral tenofovir alafenamide (TAF), and FTC (Descovy; approved by Health Canada in December 2020, but not commonly prescribed in BC unless patient has renal or bone dysfunction), long-acting injectable cabotegravir (CAB-LA; first

CAB-LA PrEP, Apretude, approved in Canada in May 2024) [8], dapivirine vaginal rings (licensed in several African nations), and lenacapavir, are not included in this study.

PrEP dispensation patterns of newly diagnosed PrEP users

We compared PrEP refill characteristics of newly diagnosed PrEP users with and without baseline drug resistant mutations. PrEP adherence more broadly in BC has been reported previously [9,10]. Using data on PrEP requisition date among newly diagnosed with PrEP use, dispensation date, and doses over time, we quantified the number of prescriptions filled; total dosage days; time from last prescription dispensation to date of first detectable viral load; time from last prescription dispensation to first antiretroviral date; for those with multiple prescriptions, the total, mean, median, minimum, and maximum days with no refill (last dosage day to next prescription filled) and the proportion days with no refill (1 – proportion of days covered).

Forty-two individuals were prescribed PrEP and later diagnosed with HIV; however, three never filled their prescription, leaving 39 newly diagnosed PrEP users. Newly diagnosed PrEP users had one to 14 PrEP prescriptions filled (median=2, mean=3.0), and 25 individuals had more than one prescription. Total dosage days ranged from 30 d to 1230 d (median=120 d; mean=219.4 d). The time from last PrEP dispensation to date of first detectable viral load ranged widely from -4 d (PrEP was likely prescribed before requisite HIV test result available) to 1605 d (median=331 d; mean=494.0 d). The distribution of times from last PrEP dispensation to date of first detectable viral load was not significantly different between those with and without baseline NRTI resistance (medians=54 d vs. 343 d; Kruskal-Wallis test, p=0.065). Time from last PrEP dispensation to date of first antiretroviral ranged from 8 d to 1611 d (median=364 d; mean=496.8 d). Those with baseline NRTI resistance had significantly less time from last PrEP dispensation to date of first detectable viral load (medians=76 vs. 373 d; Kruskal-Wallis test, p=0.045). Six individuals had recent PrEP use, similar to the definition of Misra *et al.* [11], with time from most recent dispensation to estimated seroconversion less than or equal to 90 days; two of six individuals with recent PrEP use (29 and 54 days from last dispensation to seroconversion) had baseline NRTI resistance. Nine individuals had less than 90 days from the end of their last prescribed dosage date to first detectable viral load.

For PrEP users with multiple prescriptions filled (n=20), the total number of days with no refill (from last dosage day to next prescription filled, across all prescriptions) ranged from -15 d – 753 d (median=36.5 d; mean=121.2 d), and was not significantly different between those with and without baseline NRTI resistance (Kruskal test: p=0.11). Nor were there significant differences between the median, maximum, or proportion of days with no refill between these groups (Kruskal test: p=0.36; p=0.052; p=0.052). Two PrEP users with baseline NRTI resistance who had multiple prescriptions filled also had the second and fourth highest proportion of days with no refill (0.037 and 0.40), as well as the fourth and fifth highest total number of days with no refill (203 and 290). This could be indicative of on-demand PrEP use, smaller than recommended dosing, or incomplete adherence.

Table A. HIV-1 antiretroviral therapy (ART) drug names, abbreviation codes, and classes. Drug classes include nucleoside analogue reverse transcriptase inhibitor (NRTI); non-NRTI (NNRTI); protease inhibitor (PI); and integrase strand transfer inhibitor (INSTI). Excludes broadly neutralizing antibodies, capsid inhibitors, and other classes). Components of TDF/FTC oral PrEP are marked in **bold**.

Drug name	Code	Class
**Emtricitabine**	**FTC**	**NRTI**
**Tenofovir disoproxil fumarate**	**TDF**	**NRTI**
Tenofovir alafenamide fumarate	TAF	NRTI
Lamivudine	3TC	NRTI
Abacavir	ABA/ABC	NRTI
Zidovudine	AZT	NRTI
Stavudine	D4T	NRTI
Zalcitabine	DDC	NRTI
Didanosine	DDI	NRTI
Efavirenz	DMP	NNRTI
Etravirine	ETV	NNRTI
Nevirapine	NEV	NNRTI
Rilpivirine	RPV	NNRTI
Atazanavir	ATA	PI
Darunavir	DRV	PI
Indinavir	IND	PI
Nelfinavir	NEL	PI
Saquinavir	SAQ	PI
Tipranavir	TIP	PI
Bictegravir	BCG	INSTI
Cabotegravir	CAB	INSTI
Dolutegravir	DTG	INSTI
Elvitegravir	EGV	INSTI
Raltegravir	MKS	INSTI

ART regimens of newly diagnosed PrEP users

We considered frequencies of prescribed NRTI drugs in first and most recent ART regimens among newly diagnosed PrEP users. The majority of PrEP users were initially prescribed an ART regimen containing TDF (n=15) and/or FTC (n=38). Of their most recent drug regimens, 33 were still prescribed FTC and 1 was still prescribed TDF (**Table B**).

Table B. First and most recent drug regimens of newly diagnosed PrEP users.

Drug regimen	First regimen (n newly diagnosed PrEP users)	Most recent regimen (n newly diagnosed PrEP users)
**FTC**/TAF/BCG/	22	32
**TDF/FTC**/DRV/DTG/COB/	12	0
3TC/ABA/DTG/	0	4
**TDF/FTC**/DTG/	2	0
3TC/DTG/	0	2
3TC/ABA/DRV/COB/	1	0
**FTC**/DRV/COB/TAF/BCG/	1	0
**TDF/FTC**/DRV/COB/	1	0
**TDF/FTC**/DTG/	0	1

Lineage-level viral diversification rate

In addition to comparing frequencies of clustering, we also evaluated whether newly diagnosed PrEP users and non-PrEP users differed in their lineage-level viral diversification rates, a metric reflecting historical lineage branching frequency, calculated using trees pruned to the oldest sequence per patient. Lineage-level viral diversification rate for each tip on a rooted bifurcating tree is the reciprocal sum of N_i branch lengths (l_j) from tip i to the root, with each consecutive edge (j) down-weighted by a factor of 1⁄2 [12]. For each tip, the mean lineage-level diversification rate across 100 bootstrap trees was computed.

$$Lineage - level\ viral\ diversification\ rate_i = (\sum_{j=1}^{N_i} \frac{l_j}{2^{j-1}})^{-1}$$

Supplementary results

Sociodemographic and clustering characteristics of newly HIV diagnosed with and without PrEP

We investigated differences in sociodemographic and clinical factors associated with newly HIV diagnosed with or without previous PrEP use (Table C) and associated with cluster membership stratified by newly diagnosed individuals with PrEP use or not (Table D). Among PrEP users, lower baseline CD4+ T-cell (CD4) counts were associated with clustering (Kruskal test, p=0.011), whereas in non-PrEP users, higher baseline CD4 counts were associated with clustering (p<0.001). Among newly diagnosed non-PrEP users, other factors associated with elevated risk of clustering included being a PWID (p<0.001), having previous hepatitis C virus (HCV) infection (p<0.001), which is colinear with PWID, and living in Vancouver Island health authority (p<0.001). Newly diagnosed non-PrEP users were less likely to cluster if they had heterosexual risk exposure (p=0.0015), resided in Interior or Fraser health authorities (p=0.014, p<0.001), or were infected with subtypes C, 01_AE, A1, or A6 (all p<=0.001). Since 2018, there have been new cluster members with subtypes F1 (n=2), 01_AE (n=1), B recombinant (n=1), and a B, D recombinant (n=1), however these assignments have relatively low bootstrap support and occurred in clusters predominantly comprised of subtype B (Table D).

Table C. Characteristics of newly diagnosed PrEP users and non-PrEP users from 2018-2022. Proportions compared using chi-squared test and medians compared by Kruskal-Wallis tests.

		Previous PrEP users		Non-PrEP users		
	**Parameter**	**n**	**%**	**n**	**%**	**p-value**
	**Total**	39	100	566	100	-
**Gender**	Female	0	0	83	14.7	**1.60E-02***
	Male	39	100	450	79.5	**1.09E-02***
	Transgender Male	0	0	0	0	-
	Transgender Female	0	0	6	1.1	1
**Risk Exposure**	Not reported	4	10.3	99	17.5	3.46E-01
	GBM	34	97.1	265	56.7	**9.57E-06***
	HET	1	2.9	152	32.5	**7.76E-04***
	PWID	3	8.6	114	24.4	7.99E-02
	HCV	2	5.1	109	19.3	5.08E-02
	HBV	0	0	36	6.4	**6.46E-03***
**Health Authority**	Not reported	0	0	26	4.6	3.16E-01
	Interior	1	2.6	36	6.7	5.01E-01
	Fraser	11	28.2	139	25.7	8.81E-01
	Vancouver Coastal	24	61.5	241	44.6	6.00E-02
	Vancouver Island	3	7.7	71	13.1	4.61E-01
	Northern	0	0	27	5	3.00E-01
**Subtype**	B	37	94.8	465	82.2	6.82E-02
	C	0	0	**39**	6.9	1.75E-01
	01_AE	0	0	**24**	4.2	3.74E-01
	A1	**1**	2.6	**11**	1.9	1
	A6	0	0	**7**	1.6	1
	12_BF	0	0	**3**	0.53	1
	G	0	0	**3**	0.53	1
	D	0	0	**2**	0.35	1
	F1	0	0	**2**	0.35	1
	02_AG	**1**	2.6	**1**	0.18	2.85E-01
	06_cpx	0	0	**1**	0.18	1
	07_BC	0	0	**1**	0.18	1
	33_01B	0	0	**1**	0.18	1
	35_A1D	0	0	**1**	0.18	1
	44_BF1	0	0	**1**	0.18	1
	A3	0	0	**1**	0.18	1
	B recombinant	0	0	**1**	0.18	1
	CRF 19_cpx	0	0	**1**	0.18	1
	B, D recombinant	0	0	**1**	0.18	1
		**Median**	**-**	**Median**	-	
	Log10(VL)	4.76	-	4.8	-	6.51E-01
	CD4 baseline	490	-	380	-	**3.45E-03***
	Age at first ART	32	-	37	-	**3.26E-02***

Table D. Sociodemographic and clinical factors associated with clustering among newly diagnosed PrEP users and non-PrEP users from 2018-2022. P-values reported for two-sided chi-squared tests for categorical variables and Kruskal-Wallis tests for numeric variables.

		Newly diagnosed PrEP users				Newly diagnosed non-PrEP users			
	**Parameter**	**Total**	**n clust.**	**% clust.**	**p-value**	**Total**	**n clust.**	**% clust.**	**p-value**
	**n**	39	30	76.9	-	566	303	53.5	-
**Gender**	**Female**	0	0	-	-	83	40	48.2	2.61E-01
	**Male**	39	30	76.9	-	450	251	55.8	2.02E-01
	**Transgender Male**	0	0	-	-	0	0	-	-
	**Transgender Female**	0	0	-	-	6	3	50.0	1
**Risk Exposure**	**Not reported**	4	2	50.0	4.70E-01	99	43	43.4	1.85E-01
	**GBM**	34	28	82.4	6.29E-02	265	145	54.7	3.11E-01
	**HET**	1	0	0.0	6.29E-02	152	68	44.7	**1.52E-03**
	**PWID**	3	3	100.0	2.78E-01	114	91	79.8	**6.94E-09**
	**HCV**	2	2	100.0	7.28E-01	109	86	78.9	**6.98E-08**
	**HBV**	0	0	-	8.25E-01	36	14	38.9	1.45E-01
**Health Authority**	**Not reported**	0	0	-	-	26	9	34.6	8.89E-01
	**Interior**	1	1	100.0	1	36	12	33.3	**1.39E-02**
	**Fraser**	11	9	81.8	9.74E-01	139	56	40.3	**1.50E-04**
	**Van. Coastal**	24	17	70.8	4.53E-01	241	136	56.4	4.56E-01
	**Van. Island**	3	3	100.0	7.84E-01	71	55	77.5	**5.09E-05**
	**Northern**	0	0	-	-	27	20	74.1	5.70E-02
**Subtype**	**B**	37	30	81.1	7.36E-02	465	298	64.1	**1.13E-26**
	**C**	0	0	-	-	39	0	0.0	**1.20E-11**
	**01_AE**	0	0	-	-	24	1	4.2	**2.07E-06**
	**A1**	1	0	0.0	5.17E-01	11	0	0.0	**1.00E-03**
	**A6**	0	0	-	-	7	0	0.0	**1.00E-03**
	**12_BF**	0	0	-	-	3	0	0.0	1.99E-01
	**G**	0	0	-	-	3	0	0.0	1.99E-01
	**D**	0	0	-	-	2	0	0.0	4.18E-01
	**F1**	0	0	-	-	2	2	100.0	5.42E-01
	**02_AG**	1	0	0.0	5.17E-01	1	0	0.0	9.43E-01
	**06_cpx**	0	0	-	-	1	0	0.0	9.43E-01
	**07_BC**	0	0	-	-	1	0	0.0	9.43E-01
	**33_01B**	0	0	-	-	1	0	0.0	9.43E-01
	**35_A1D**	0	0	-	-	1	0	0.0	9.43E-01
	**44_BF1**	0	0	-	-	1	0	0.0	9.43E-01
	**A3**	0	0	-	-	1	0	0.0	9.43E-01
	**B recomb.**	0	0	-	-	1	1	100.0	1
	**CRF 19_cpx**	0	0	-	-	1	0	0.0	9.43E-01
	**B, D recomb.**	0	0	-	-	1	1	100.0	1
		**Median, all**	**Median, clust.**			**Median, all**	**Median, clust.**		
	**log10 (VL)**	4.76	4.75	-	9.47E-01	4.80	4.85	-	1.12E-01
	**CD4 baseline**	490	407	-	**1.13E-02**	380	440	-	**8.09E-05**
	**Age first ARV**	32	32.5	-	7.39E-01	37	36	-	2.57E-01

Phylogenetic clustering and diversification rates of newly diagnosed with and without PrEP

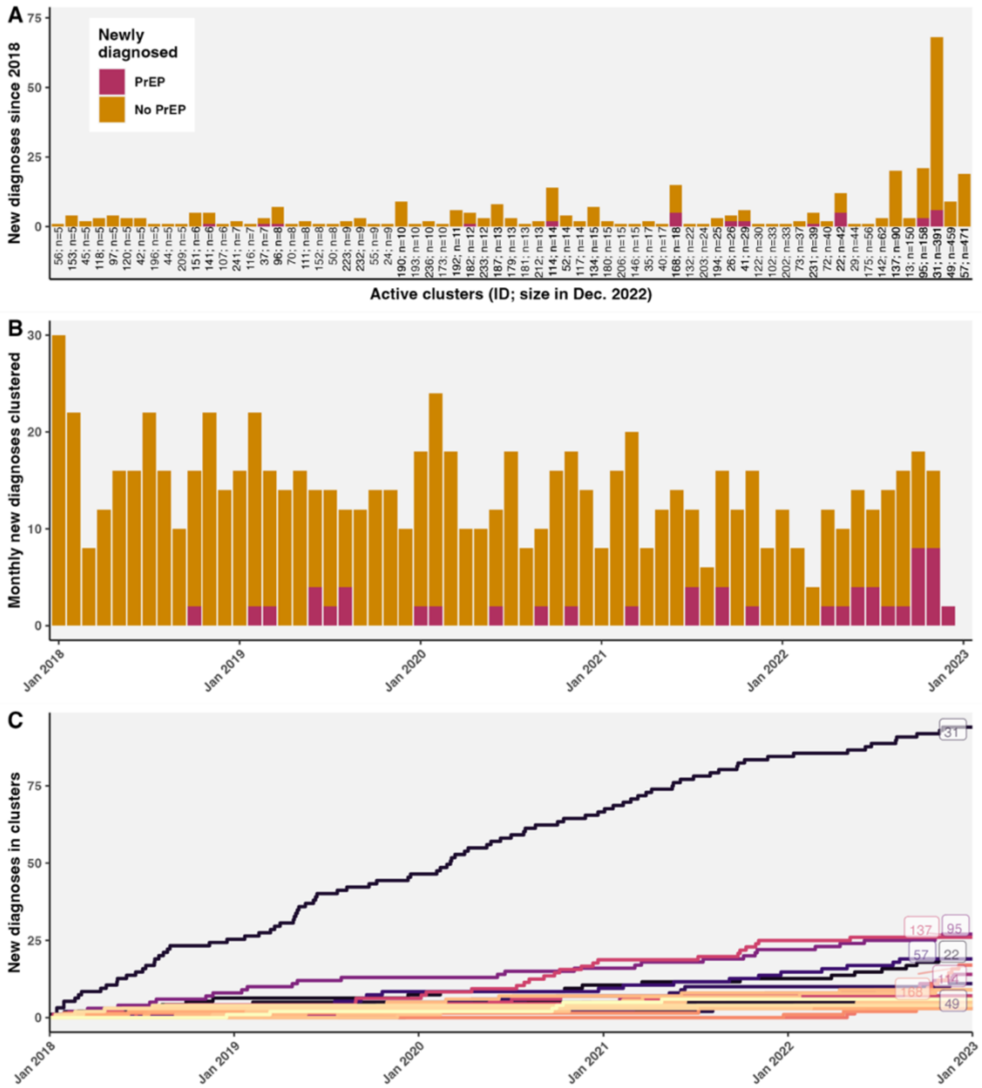

Fig A. New diagnoses in active HIV phylogenetic clusters in BC since 2018. **A)** Total new diagnoses in active clusters, annotated with cluster ID; cluster size at the end of study period in December 2022. Bars are colored by PrEP use. **B)** Monthly new diagnoses that clustered during the study period, colored by PrEP use. **C)** New diagnoses in the study period by cluster. Annotated with cluster ID for the eight largest clusters.

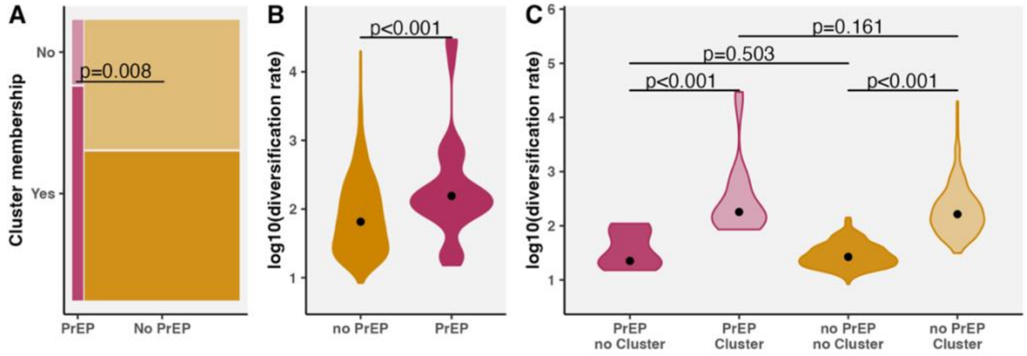

Fig B. Clustering and lineage-level viral diversification rate among newly diagnosed with and without PrEP use. A) Comparison of the proportion of individuals who clustered (membership, yes or no) by PrEP use, using a two-sided chi-squared test. **B)** Distribution of viral lineage-level diversification rate among newly diagnosed PrEP users and non-PrEP users, compared using a Kruskal-Wallis test**. C)** Viral lineage-level diversification rates were compared by PrEP use and clustering using pairwise Mann-Whitney tests.

Comparison of cluster identification under different tree rooting strategies

We investigated the sensitivity of cluster identification to rooting strategies for maximum likelihood divergence-scaled HIV-1 phylogenetic trees. Starting with the same set of 100 bootstrap partial *pol* trees from FastTree, we compared the default midpoint-rooted (MPR) trees, a binarized MPR tree, to those rooted using three different outgroups (oldest subtype B, G, and H in the BC data), along with a subset of 10 trees, due to computational feasibility, rooted under the assumption of a strict molecular clock using root-to-tip regression in TempEst [13], and we compared clusters identified using a 50, 70, and 90% threshold for the percent of bootstrap trees connecting individuals. We identified clusters with 5 or more members linked by less than 0.02 substitutions/site tree distance, and then compared the number of clusters and distribution of cluster size (Fig C).

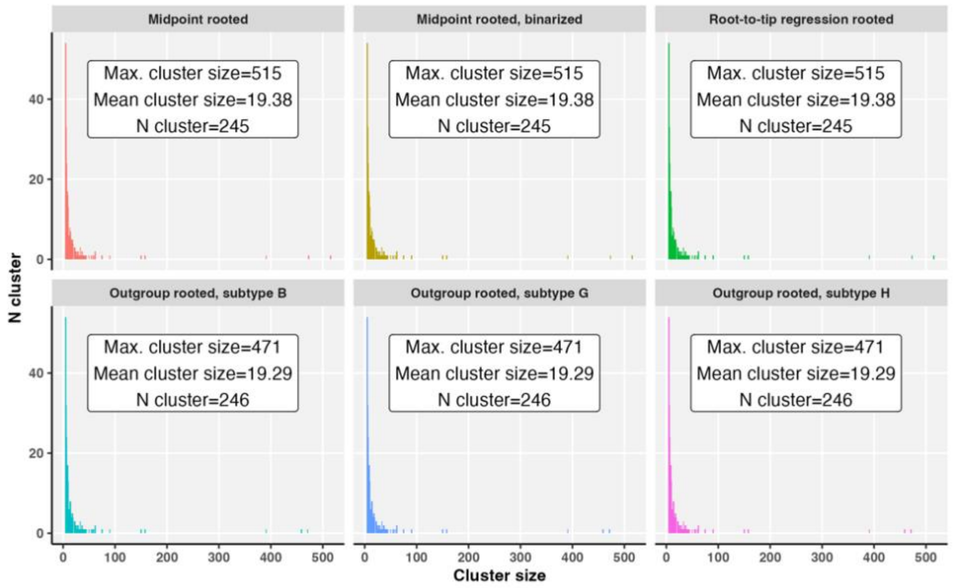

Fig C. Distribution of phylogenetic cluster sizes identified using multiple rooting strategies. Trees were rooted using midpoint (Fasttree default), midpoint then binarized, root-to-tip regression, or outgroup rooting on oldest subtype B, G, or H. Annotations for the maximum and mean cluster size, and total number of clusters.

Baseline drug resistance of newly diagnosed individuals with and without PrEP

Of NRTI resistance-associated mutations (RAMs), NRTI K70R was most common at baseline in the entire cohort and is increasing in prevalence over time (Fig D). The next most frequently detected baseline RAMs were NRTI resistance mutation, K103N, and NNRTI resistance mutation, T215E. K103N has been identified independently in six active (defined as new cases since 2018) phylogenetic clusters, while baseline T215E is present in all 14 members of cluster 114 (Fig L).

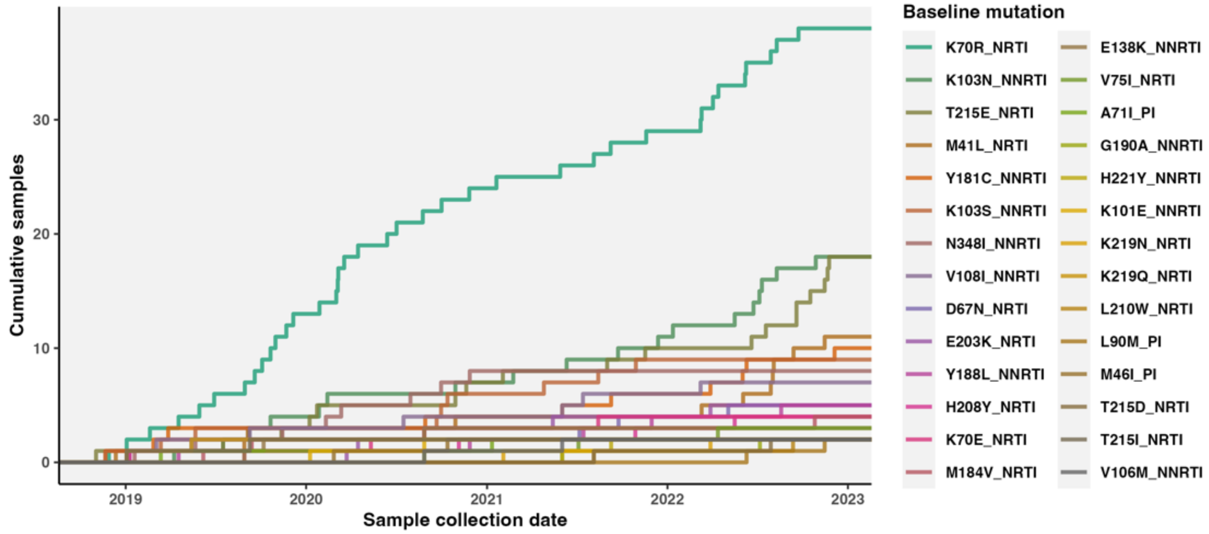

Fig D. Cumulative detection of baseline treatment selected mutations (TSMs) among newly diagnosed 2018-2022.

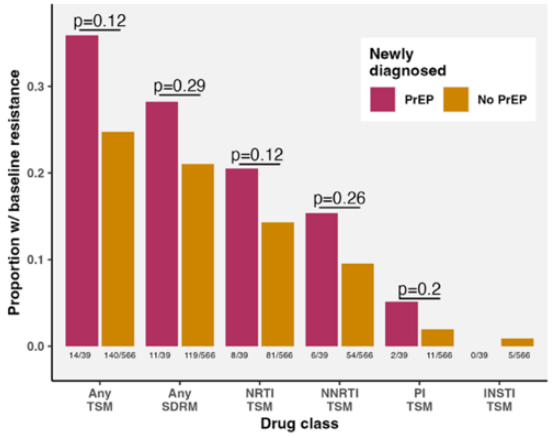

Fig E. Proportion newly diagnosed with or without PrEP with any baseline TSM or SDRM according to Stanford HIVdb definitions, by drug class.

Drug resistance scores of newly diagnosed individuals with and without PrEP

Differences in M184V manifested as elevated baseline drug resistance scores for lamivudine (3TC; Kruskal-Wallis, p<0.001), emtricitabine (FTC; p<0.0001), and abacavir (ABC; p=0.020) in PrEP users compared to non-PrEP users (Fig F). Newly diagnosed PrEP users also had significantly elevated didanosine (DDI) drug resistance scores (p=0.019), driven by co-occurring thymidine analog mutations, T215E and M41L.

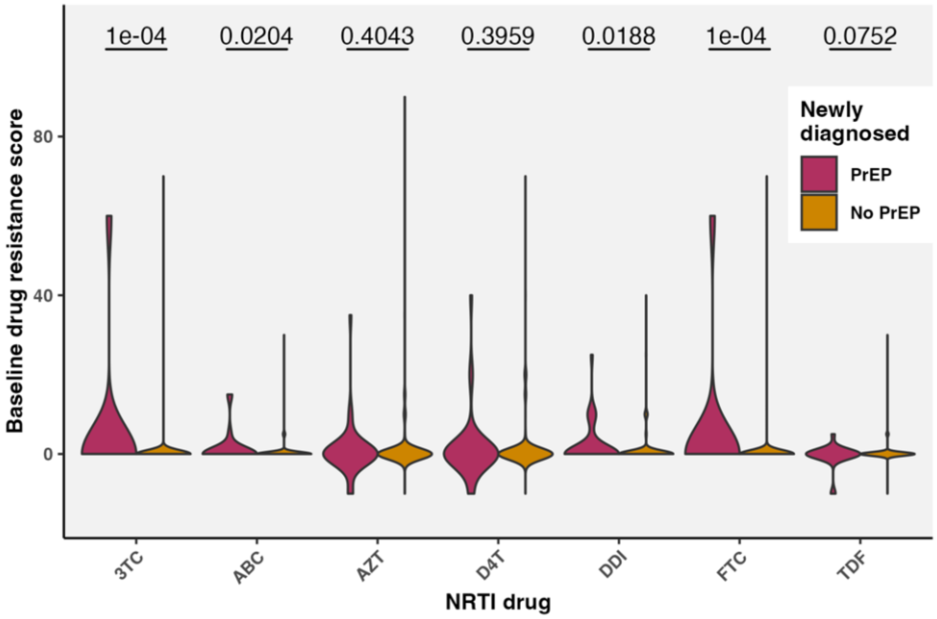

Fig F. NRTI drug resistance scores in newly diagnosed with and without PrEP. Scores calculated using Stanford HIVdb. Drugs include 3TC, ABC, zidovudine (AZT), stavudine (D4T), DDI, FTC, and TDF. P-values were calculated using Kruskal-Wallis tests.

Active HIV-1 phylogenetic clusters in BC

53 active phylogenetic clusters (with at least 1 new case since January 1, 2018) and a minimum size of 10 individuals were characterized by size in January 2023, new cases since 2018 and 2022; median date of first plasma viral load (VL), baseline CD4 count, baseline VL, age at first ART, age 2023; % key population; % regional health authorities relative to reported (Table E).

Table E. Characteristics of active phylogenetic clusters with at least 1 new case since Jan. 1, 2018 and size 10 or larger.

		New cases			Median						Key population					Health Authority				
ID	Size	Since 2018	Since 2018, PrEP	Since 2022	Date first VL	CD4 base	VL base	Age first ART	Age in 2023	% report	% GBM	% HET	% PWID	% HCV	% report	% Van. Coastal	% Van. Island	% Fraser	% North	% Interior
57	471	19	0	5	2003-11-13	250	93771	39	58	82.0	5.7	45.9	85.8	78.2	94.5	36.4	20.0	20.4	19.1	4.0
49	459	11	0	1	2000-06-05	251	55200	40	60	85.6	4.8	47.3	90.8	87.1	94.3	55.4	7.6	20.1	9.2	7.6
31	391	94	8	11	2014-04-07	390	94537	35	44	73.9	88.9	11.4	16.6	12.5	94.6	60.5	19.5	18.6	0.3	1.1
95	158	27	6	5	2014-11-23	458	63537	35	42	63.9	92.1	8.9	17.8	12.3	98.1	63.9	3.9	31.6	0.6	0.0
13	150	3	0	0	2008-09-26	330	86600	39	54	66.0	88.9	7.1	13.1	10.4	97.3	76.7	6.2	15.8	0.7	0.7
137	90	26	0	1	2008-04-20	260	100010	37	53	84.4	0.0	47.4	88.2	82.8	90.0	70.4	4.9	17.3	6.2	1.2
142	62	6	0	2	2012-03-13	290	33447	43	55	71.0	75.0	27.3	20.5	26.3	88.7	32.7	56.4	7.3	0.0	3.6
201	62	2	0	0	1998-04-24	270	37000	38	60	90.3	8.9	55.4	89.3	89.7	98.4	65.6	3.3	16.4	3.3	11.5
175	56	1	0	0	2005-07-08	200	44897	39	56	91.1	2.0	21.6	98.0	100.0	92.9	23.1	3.8	11.5	57.7	3.8
29	44	1	0	0	2002-09-05	175	100010	42	59	79.5	0.0	42.9	91.4	87.5	88.6	71.8	0.0	23.1	2.6	2.6
22	42	19	5	7	2017-04-02	455	97000	39	44	78.6	100.	0.0	12.1	10.8	90.5	73.7	2.6	21.1	0.0	2.6
72	40	3	0	0	2013-06-24	420	39761	37	49	50.0	80.0	45.0	15.0	10.3	97.5	64.1	0.0	23.1	2.6	10.3
234	39	6	1	0	2015-04-03	270	100010	33	44	84.6	78.8	18.2	15.2	13.2	100.0	30.8	12.8	7.7	0.0	48.7
73	37	2	0	0	2009-11-16	305	90225	40	52	64.9	87.5	12.5	16.7	21.2	91.9	44.1	14.7	35.3	0.0	5.9
177	37	1	0	0	2007-07-06	255	59000	41	53	89.2	15.2	51.5	90.9	88.9	97.3	69.4	2.8	25.0	0.0	2.8
109	36	1	0	0	1999-04-10	210	59800	44	63	88.9	6.3	46.9	96.9	96.9	94.4	58.8	23.5	14.7	2.9	0.0
219	36	4	0	0	2006-06-14	270	140000	40	55	75.0	70.4	29.6	22.2	18.2	94.4	61.8	0.0	38.2	0.0	0.0
102	33	1	0	0	2002-09-07	315	67850	40	57	69.7	82.6	26.1	26.1	21.2	97.0	62.5	25.0	12.5	0.0	0.0
206	33	2	0	0	2002-02-09	190	98405	36	56	84.8	14.3	60.7	85.7	79.3	87.9	69.0	3.4	24.1	0.0	3.4
217	33	4	0	0	2011-08-25	245	83707	37	48	69.7	0.0	56.5	60.9	42.9	90.9	10.0	0.0	6.7	80.0	3.3
122	30	2	0	0	2006-06-17	330	37847	37	53	90.0	3.7	29.6	92.6	89.7	96.7	20.7	58.6	20.7	0.0	0.0
41	29	7	2	0	2013-12-02	510	50370	34	41	69.0	80.0	10.0	25.0	16.0	93.1	40.7	33.3	14.8	3.7	7.4
93	28	1	0	0	2012-08-15	360	109908	29	42	60.7	94.1	0.0	17.6	8.0	96.4	70.4	0.0	18.5	7.4	3.7
26	26	5	2	0	2003-07-29	260	100010	31	46	76.9	85.0	5.0	20.0	11.5	100.0	96.2	0.0	3.8	0.0	0.0
91	25	1	0	0	2011-05-12	323	97567	37	48	52.0	69.2	7.7	38.5	20.0	96.0	54.2	4.2	37.5	0.0	4.2
209	25	5	0	2	2016-09-15	275	46744	38	44	92.0	4.3	39.1	78.3	84.0	92.0	65.2	4.3	17.4	4.3	8.7
218	24	1	0	0	2011-10-21	295	41503	35	45	70.8	0.0	35.3	88.2	80.0	75.0	5.6	5.6	5.6	83.3	0.0
132	22	1	0	0	2000-12-02	185	53600	45	61	86.4	0.0	36.8	100.0	95.5	100.0	81.8	0.0	18.2	0.0	0.0
14	19	1	0	0	2007-05-28	300	42349	43	56	73.7	85.7	0.0	35.7	31.6	94.7	83.3	0.0	16.7	0.0	0.0
168	18	17	5	17	2022-10-08	535	61200	44	45	72.2	100.0	0.0	0.0	0.0	83.3	80.0	0.0	20.0	0.0	0.0
35	17	2	0	0	2008-01-15	315	40300	37	52	82.4	92.9	21.4	14.3	12.5	100.0	58.8	5.9	29.4	5.9	0.0
40	17	3	0	0	2011-08-03	390	84705	41	58	88.2	86.7	40.0	13.3	18.8	82.4	57.1	14.3	28.6	0.0	0.0
134	15	7	0	0	2017-07-17	480	15250	37	50	80.0	0.0	41.7	91.7	91.7	80.0	66.7	0.0	33.3	0.0	0.0
146	15	1	0	1	2013-01-23	280	70789	38	46	53.3	75.0	25.0	12.5	8.3	80.0	41.7	16.7	33.3	8.3	0.0
194	15	5	0	2	2013-03-13	435	47245	36	44	86.7	15.4	61.5	92.3	85.7	93.3	78.6	0.0	21.4	0.0	0.0
221	15	1	0	0	2007-08-02	250	37072	35	49	66.7	100.0	0.0	10.0	21.4	93.3	78.6	14.3	7.1	0.0	0.0
52	14	4	0	0	2008-10-30	440	2662	36	44	71.4	70.0	60.0	30.0	7.1	100.0	57.1	0.0	35.7	7.1	0.0
114	14	14	2	6	2021-10-09	400	136000	23	25	100.0	92.9	7.1	7.1	0.0	92.9	30.8	15.4	30.8	0.0	23.1
117	14	2	0	0	2009-11-30	467	37289	43	59	71.4	90.0	40.0	30.0	21.4	92.9	30.8	0.0	61.5	0.0	7.7
193	13	3	0	0	2004-07-05	300	78413	41	65	84.6	0.0	36.4	100.0	100.0	76.9	80.0	0.0	20.0	0.0	0.0
196	13	3	0	0	2015-02-02	260	209844	34	47	92.3	0.0	83.3	58.3	38.5	100.0	53.8	7.7	38.5	0.0	0.0
202	13	9	0	1	2019-06-12	460	57700	38	42	84.6	0.0	45.5	100.0	100.0	92.3	58.3	0.0	41.7	0.0	0.0
227	13	2	0	0	2010-08-23	230	66535	28	39	69.2	100.0	11.1	11.1	8.3	92.3	41.7	0.0	58.3	0.0	0.0
245	13	1	0	0	2016-04-09	494	160895	27	34	84.6	81.8	18.2	9.1	0.0	100.0	23.1	0.0	69.2	0.0	7.7
179	12	3	0	2	2009-12-07	370	15945	36	45	75.0	100.0	11.1	0.0	0.0	100.0	8.3	66.7	16.7	0.0	8.3
197	12	5	1	2	2017-11-22	477	41078	33	42	91.7	81.8	18.2	0.0	10.0	91.7	45.5	0.0	18.2	0.0	36.4
207	11	6	0	0	2019-11-20	560	240000	36	42	90.9	10.0	30.0	80.0	81.8	81.8	22.2	0.0	55.6	11.1	11.1
33	10	1	0	0	2010-05-22	500	49606	37	55	20.0	100.0	0.0	0.0	0.0	80.0	87.5	0.0	12.5	0.0	0.0
173	10	1	0	0	2011-05-21	345	107236	41	51	80.0	100.0	0.0	12.5	22.2	90.0	77.8	0.0	11.1	11.1	0.0
182	10	3	0	0	2008-02-28	150	120023	48	61	70.0	28.6	85.7	28.6	50.0	80.0	12.5	0.0	37.5	50.0	0.0
203	10	1	0	0	2003-01-21	240	83005	40	55	90.0	11.1	44.4	100.0	88.9	90.0	66.7	11.1	11.1	0.0	11.1
205	10	9	0	2	2020-08-22	660	171000	31	33	60.0	0.0	33.3	100.0	100.0	80.0	87.5	0.0	12.5	0.0	0.0
208	10	1	0	0	2010-11-16	360	23149	40	51	80.0	0.0	50.0	62.5	60.0	100.0	50.0	0.0	50.0	0.0	0.0

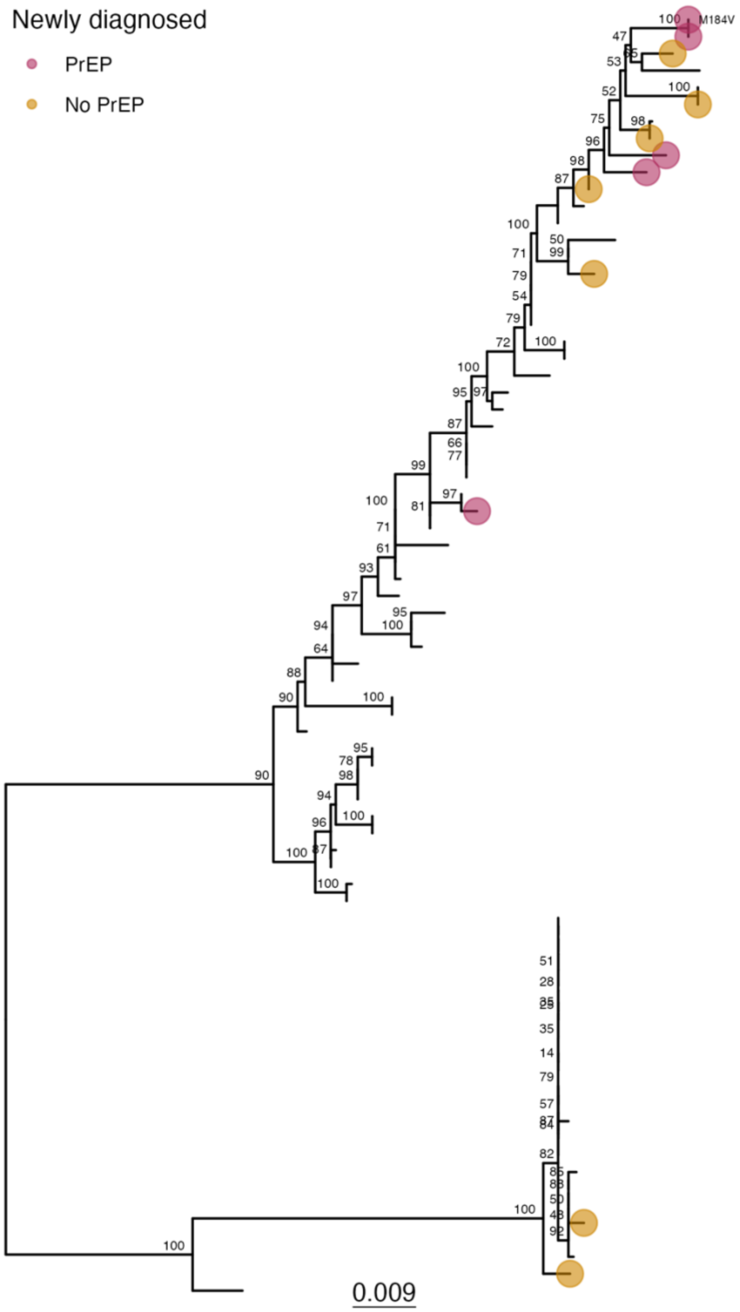

Fig G. Maximum likelihood phylogenetic tree of HIV cluster 22. Tree was inferred with partial *pol* alignment stripped of drug resistance mutation codons in IQ-TREE with a generalized time reversible model, and rooted using root-to-tip regression. Nodes annotated with ultrafast bootstrap support values. Tips colored for viruses from newly diagnosed (2018-2022) PrEP users and non-PrEP users, and annotated with NRTI resistance associated mutations including M184IV. Multiple sequences shown per participant.

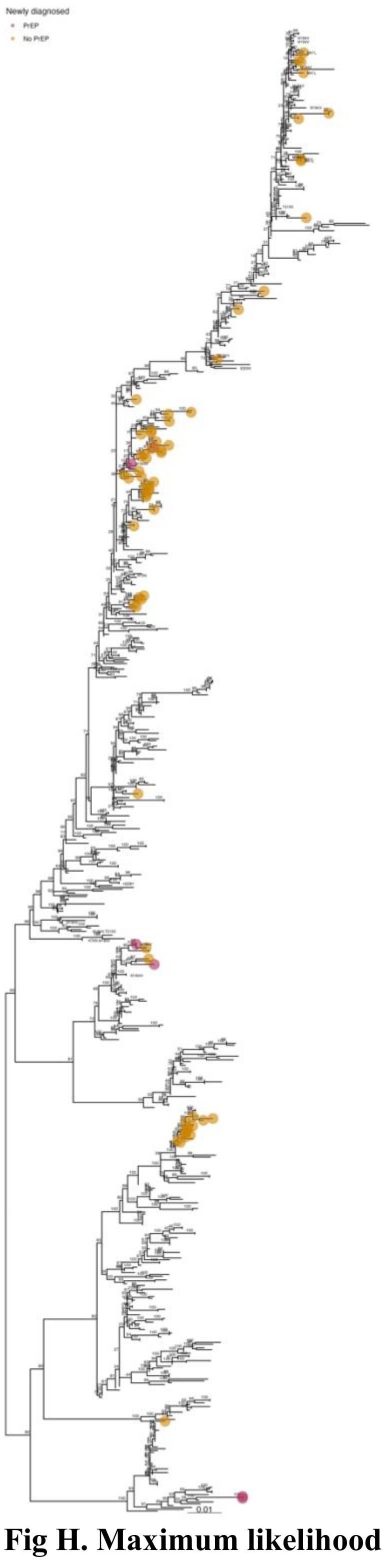

Fig H. Maximum likelihood phylogenetic tree of HIV cluster 31.

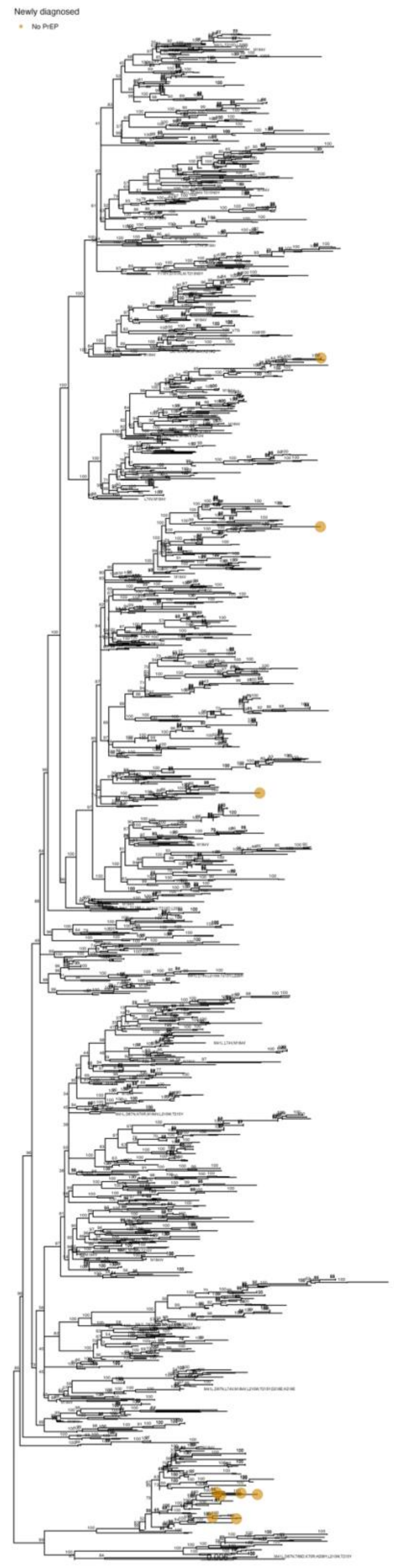

Fig I. Maximum likelihood phylogenetic tree of HIV cluster 49.

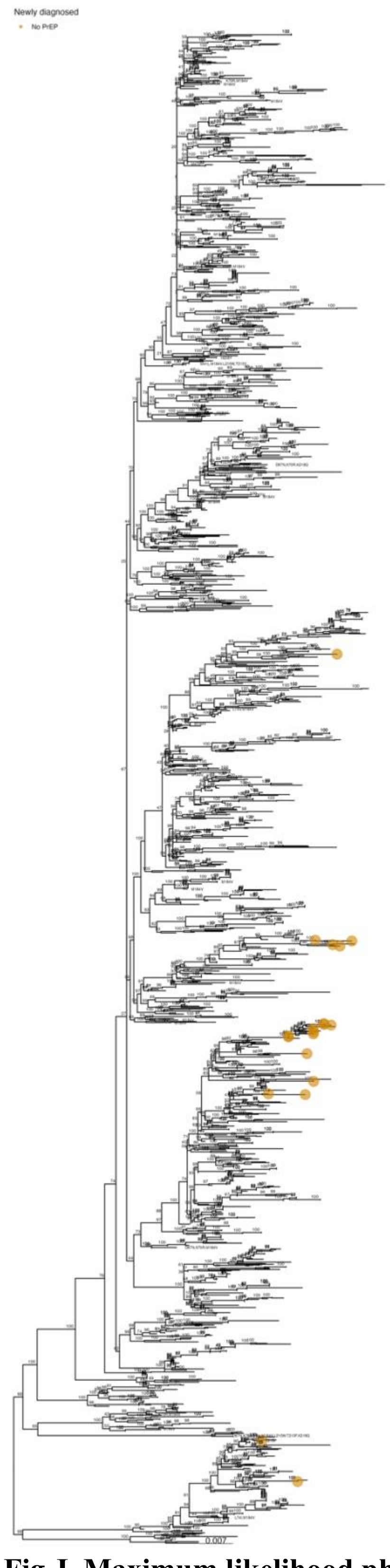

Fig J. Maximum likelihood phylogenetic tree of HIV cluster 57.

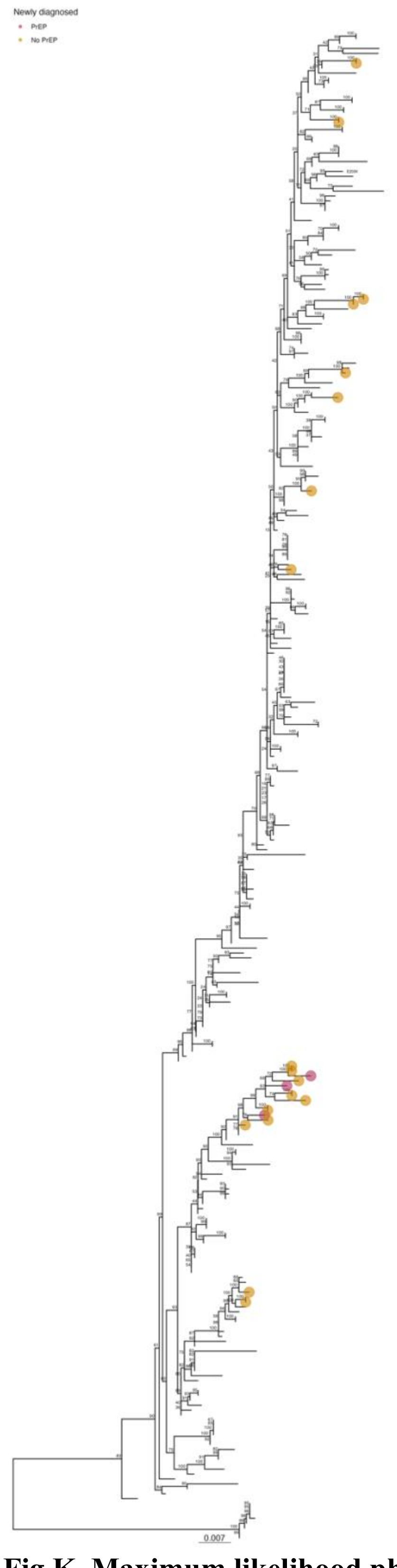

Fig K. Maximum likelihood phylogenetic tree of HIV cluster 95.

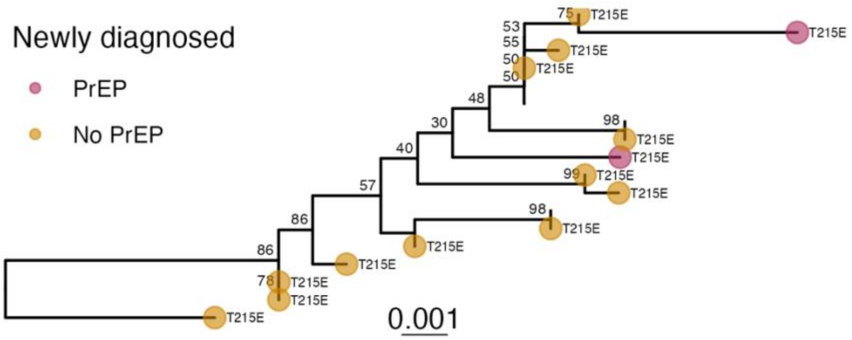

Fig L. Maximum likelihood phylogenetic tree of HIV cluster 114.

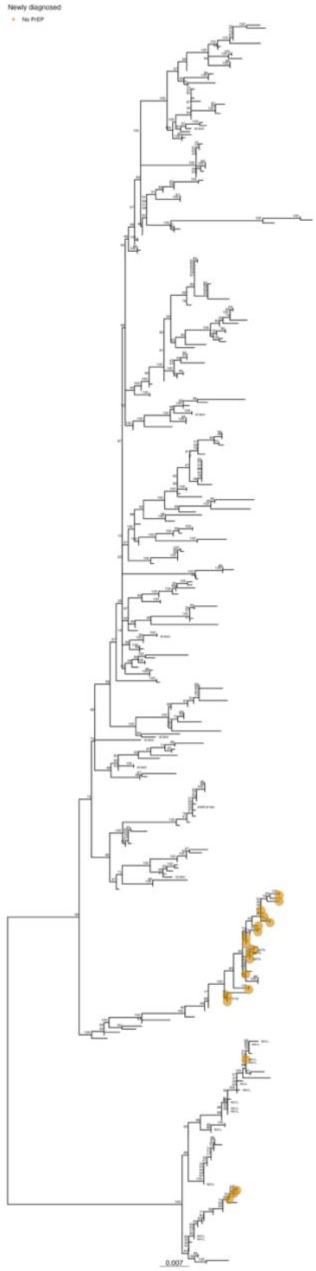

Fig M. Maximum likelihood phylogenetic tree of HIV cluster 137.

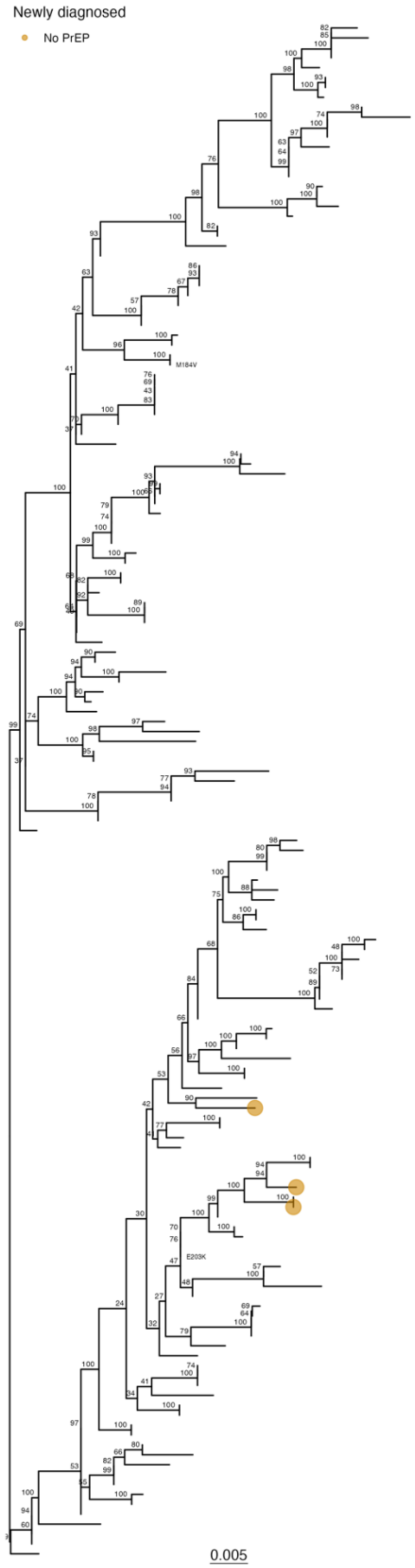

26 **Fig N. Maximum likelihood phylogenetic tree of HIV cluster 142.**

HIV effective reproduction number (R_e) in British Columbia and key populations

We calculated annual HIV incidence and prevalence per 100,000 (Fig O) in BC for 14,919 cumulative DTP participants, excluding those with previous ART history (HIV-positive migrant), adjusted by quarterly population size [14]. The prevalent PLWH population size was calculated as cumulative new cases minus deaths and emigrants by quarter; and growth rate as new cases per prevalent case per quarter.

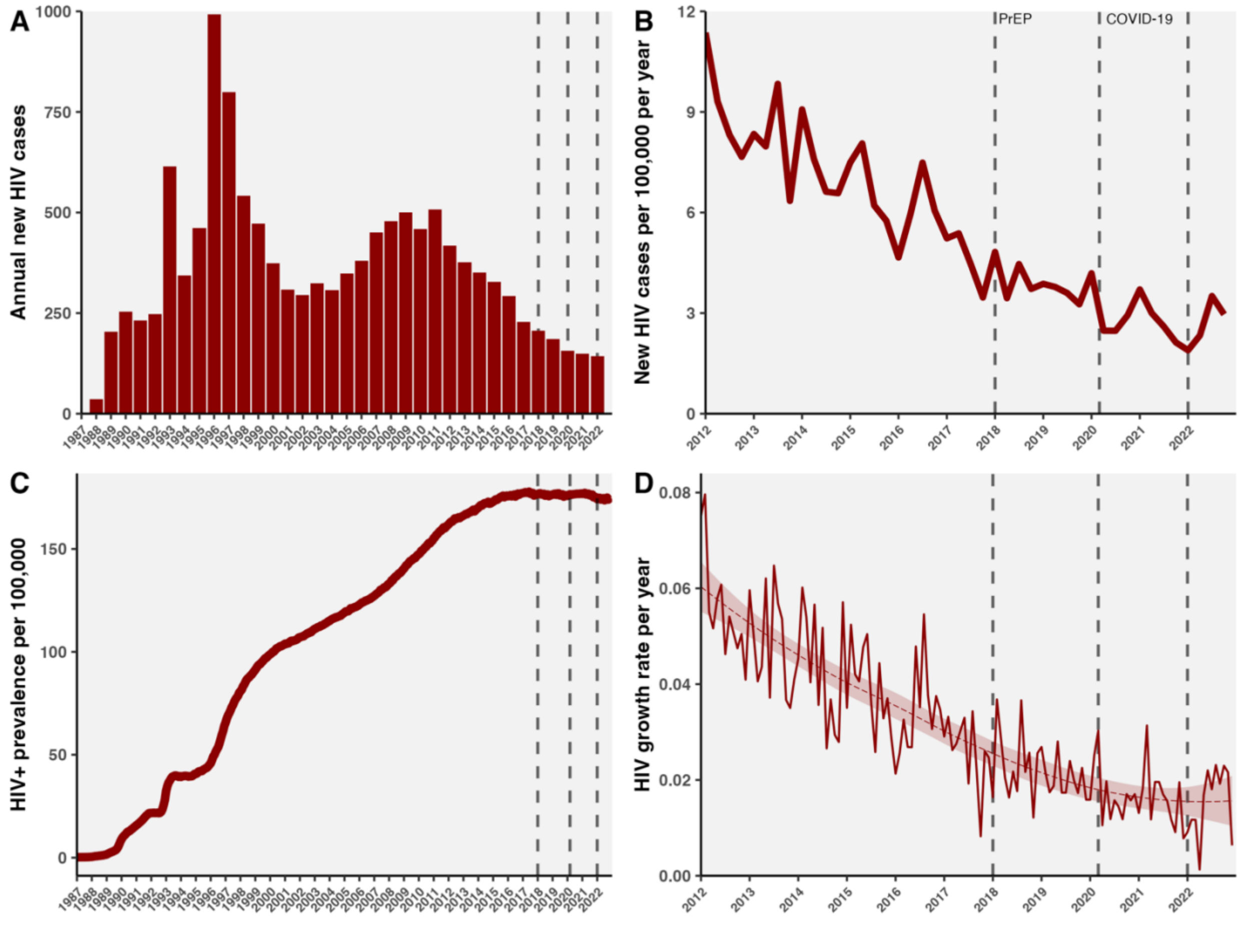

Fig O. HIV incidence and prevalence in BC based on DTP participants, in the context of PrEP availability. A) Annual new HIV cases in BC from 1987 to the end of 2022, estimated by the date of first ART, excluding those with previous ART history (HIV-positive migrant). **B)** New HIV cases in BC per 100,000 per year calculated quarterly from 2012 to 2022, normalized to BC population size by quarter [14]. **C)** HIV prevalence per 100,000 in BC from 1987 to 2022, calculated as the cumulative sum of new cases and HIV-positive migrants, subtracting the number of deaths and emigrants. **D)** HIV growth rate per year in BC from 2012 to 2022, calculated as new cases divided by the prevalent population size in each calendar month converted to per year. Dotted vertical lines denote PrEP availability in January 2018 onwards and COVID-19 pandemic interventions from March 2020 to January 2022.

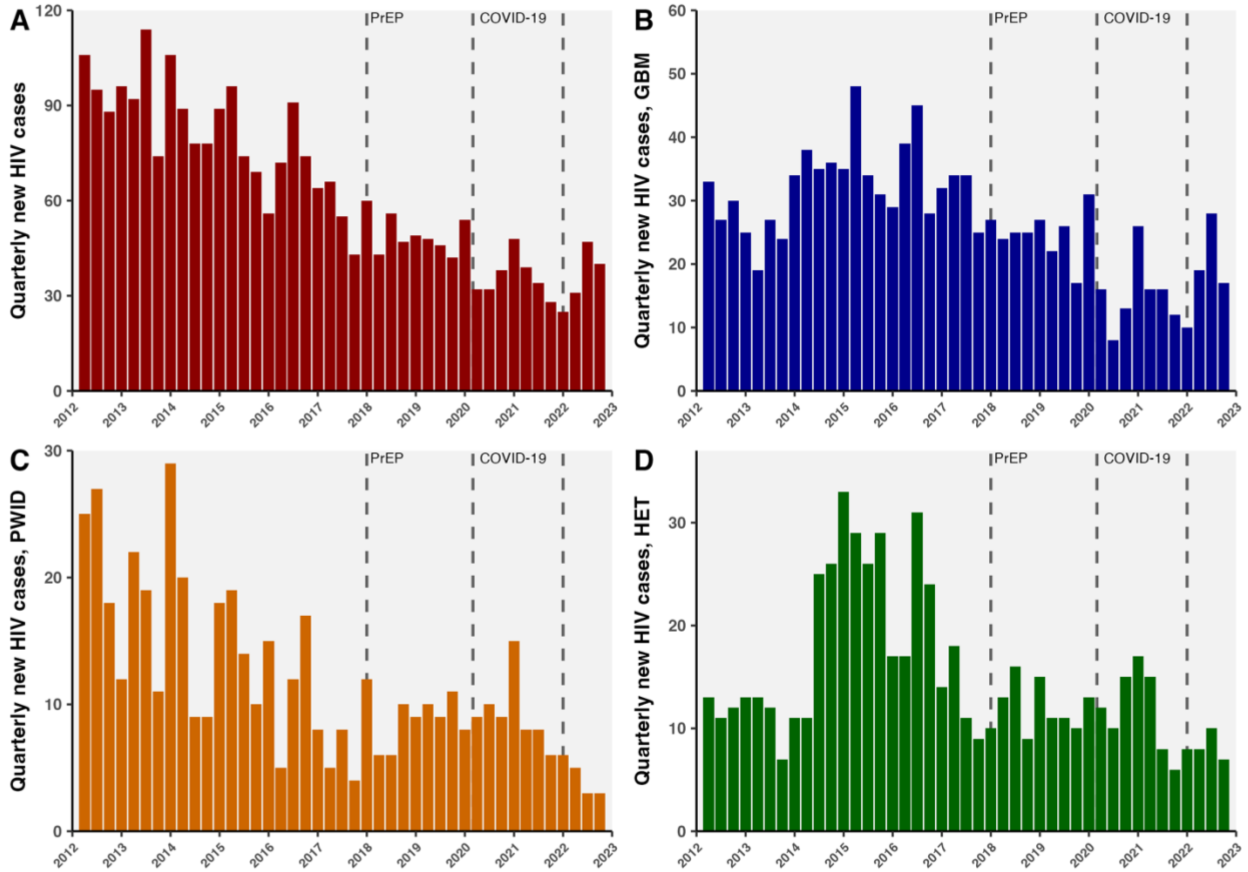

Fig P. Quarterly new HIV cases from January 2012 to February 2023 by key population. For **A)** all BC residents, **B)** GBM, **C)** PWID, and **D)** HET. Individuals with multiple risk factors are represented in multiple panels. Dotted vertical lines denote PrEP availability in January 2018 onwards and COVID-19 pandemic interventions from March 2020 to January 2022.

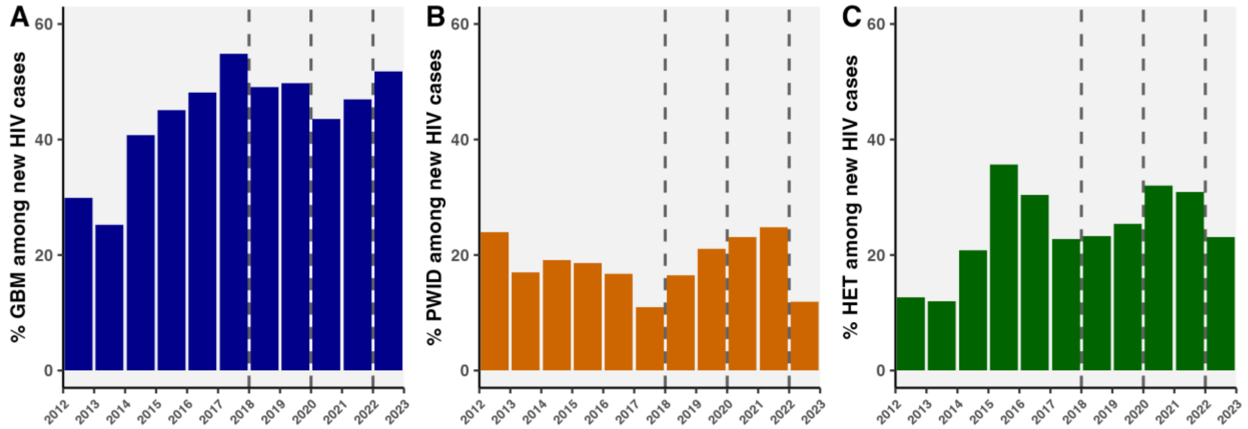

Fig Q. Percentage of annual new HIV cases within key populations. A) GBM, **B)** PWID, **C)** HET. Individuals who reported multiple risk factors are represented in multiple panels and not all individuals reported risk factors. Dotted vertical lines denote PrEP availability in January 2018 onwards and COVID-19 pandemic interventions from March 2020 to January 2022.

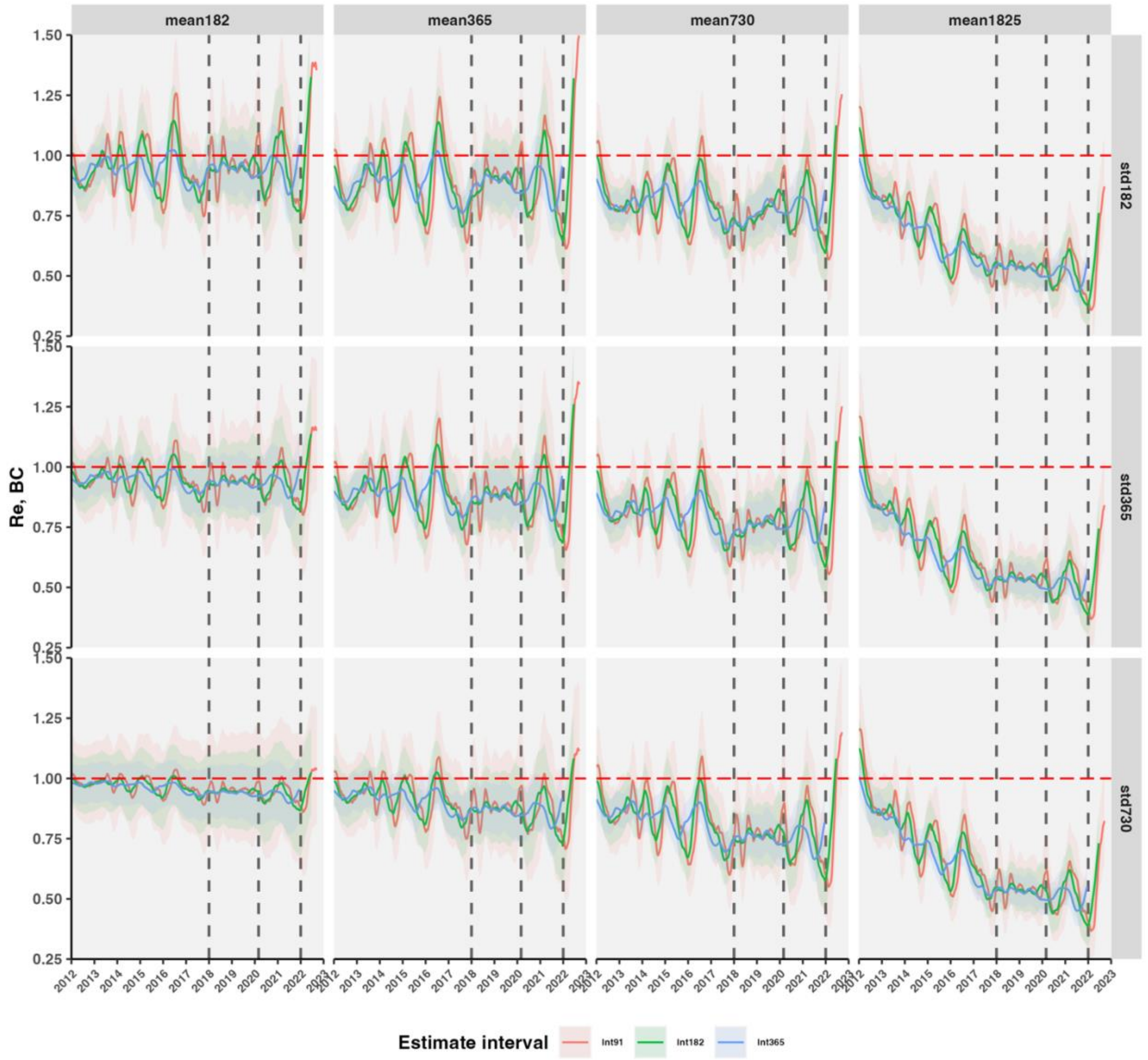

Fig R. HIV R_e in BC from 2012 to 2022 under varying serial interval assumptions. Instantaneous R_e was estimated in EpiEstim with gamma-distributed serial intervals for multiple parameter sets with means (by columns) of 0.5 year (y) (182 days (d)), 1 y (365 d), 2 y (730 d), or 5 y (1825 d); standard deviations (std or sd; by rows) of 0.5 y, 1 y, or 2 y; and estimate windows (by color) of 0.25 y (91 d), 0.5 y, or 1 y. R_e were smoothed with k=90 d, right-aligned (i.e. average over past 90 d).

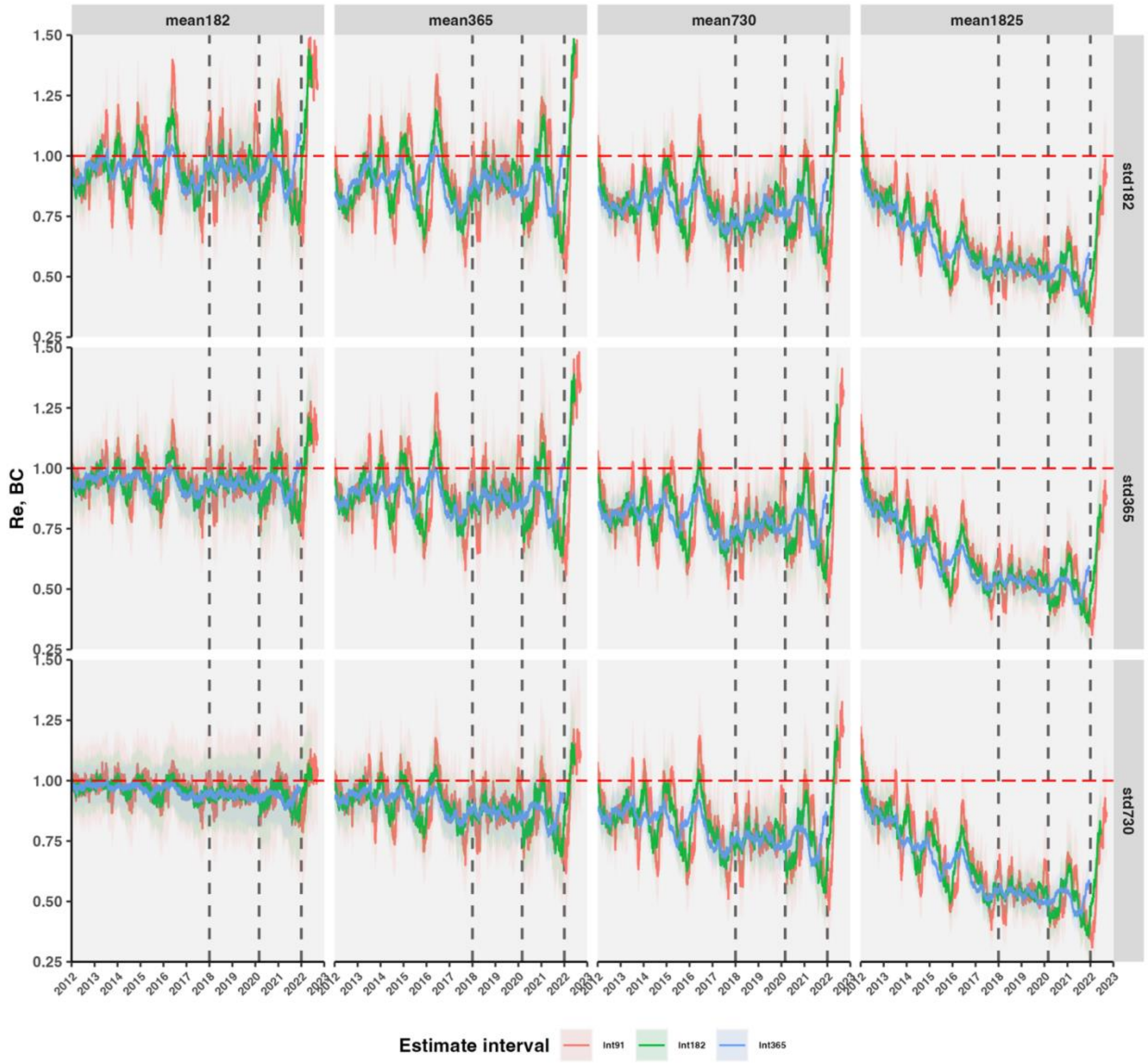

62
63 **Fig S. HIV R_e in BC as in Fig R, with no smoothing.**

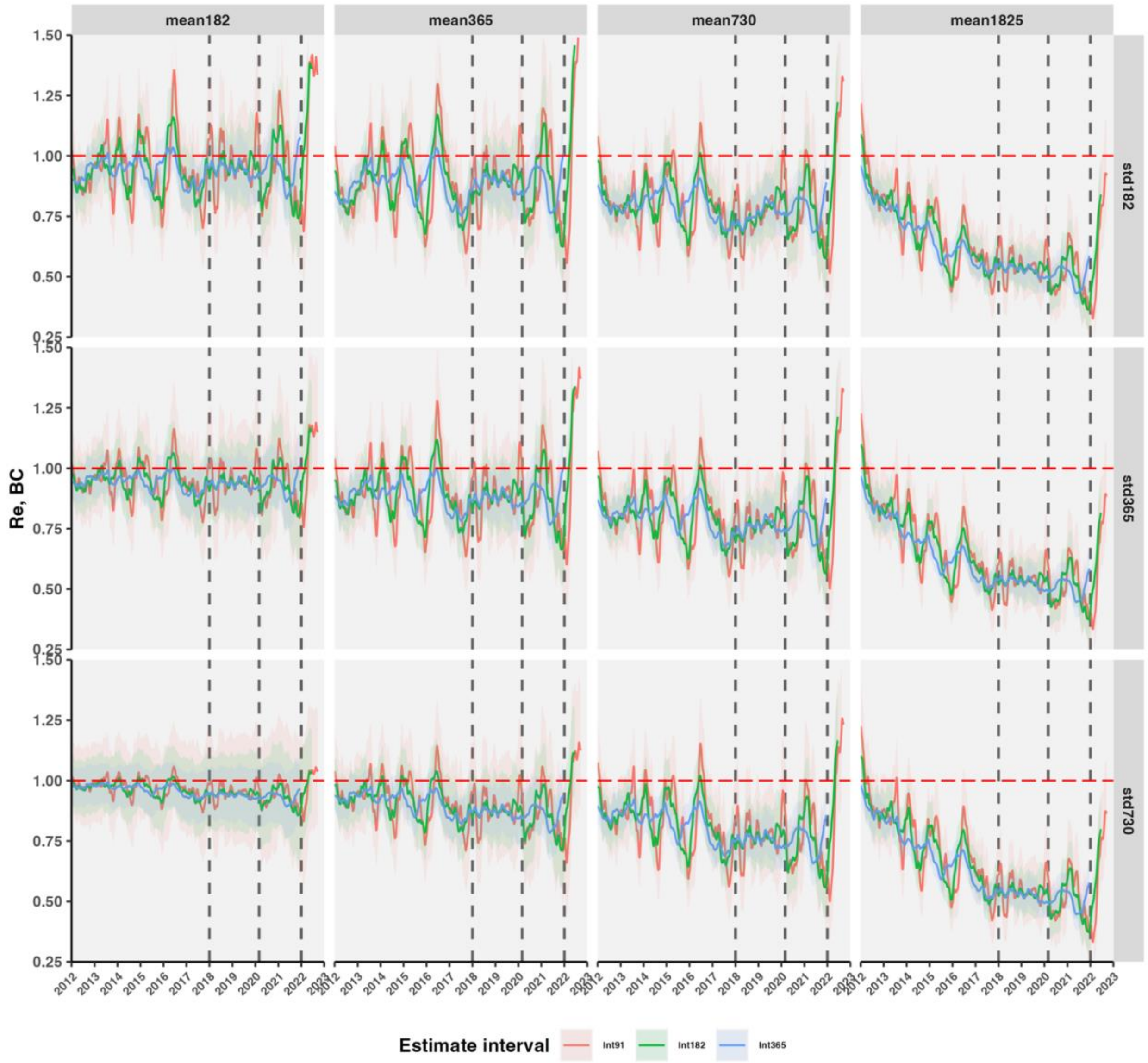

Fig T. HIV R_e in BC as in Fig R, with 30 d smoothing.

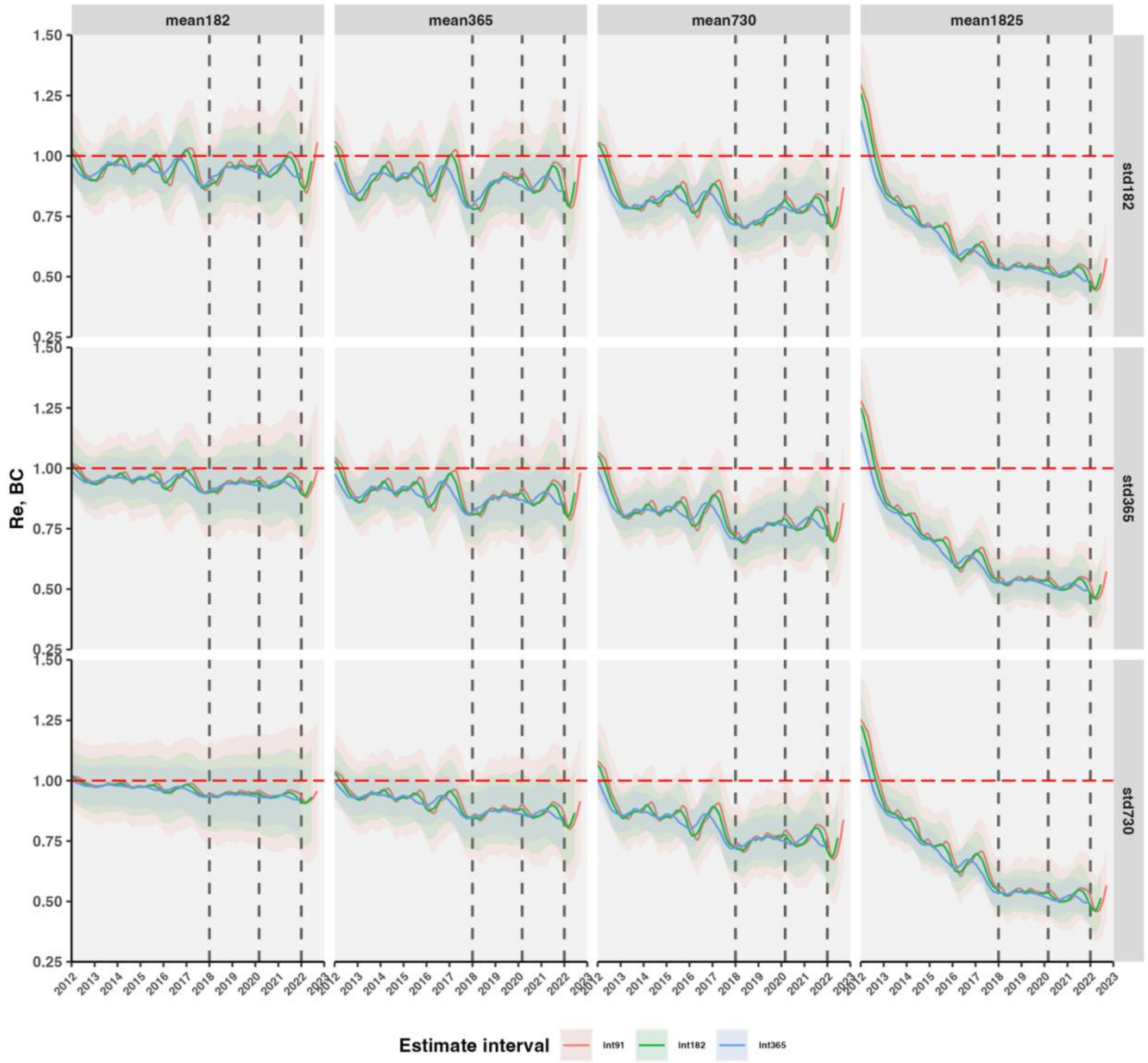

Fig U. HIV R_e in BC as in Fig R, with 365 d smoothing.

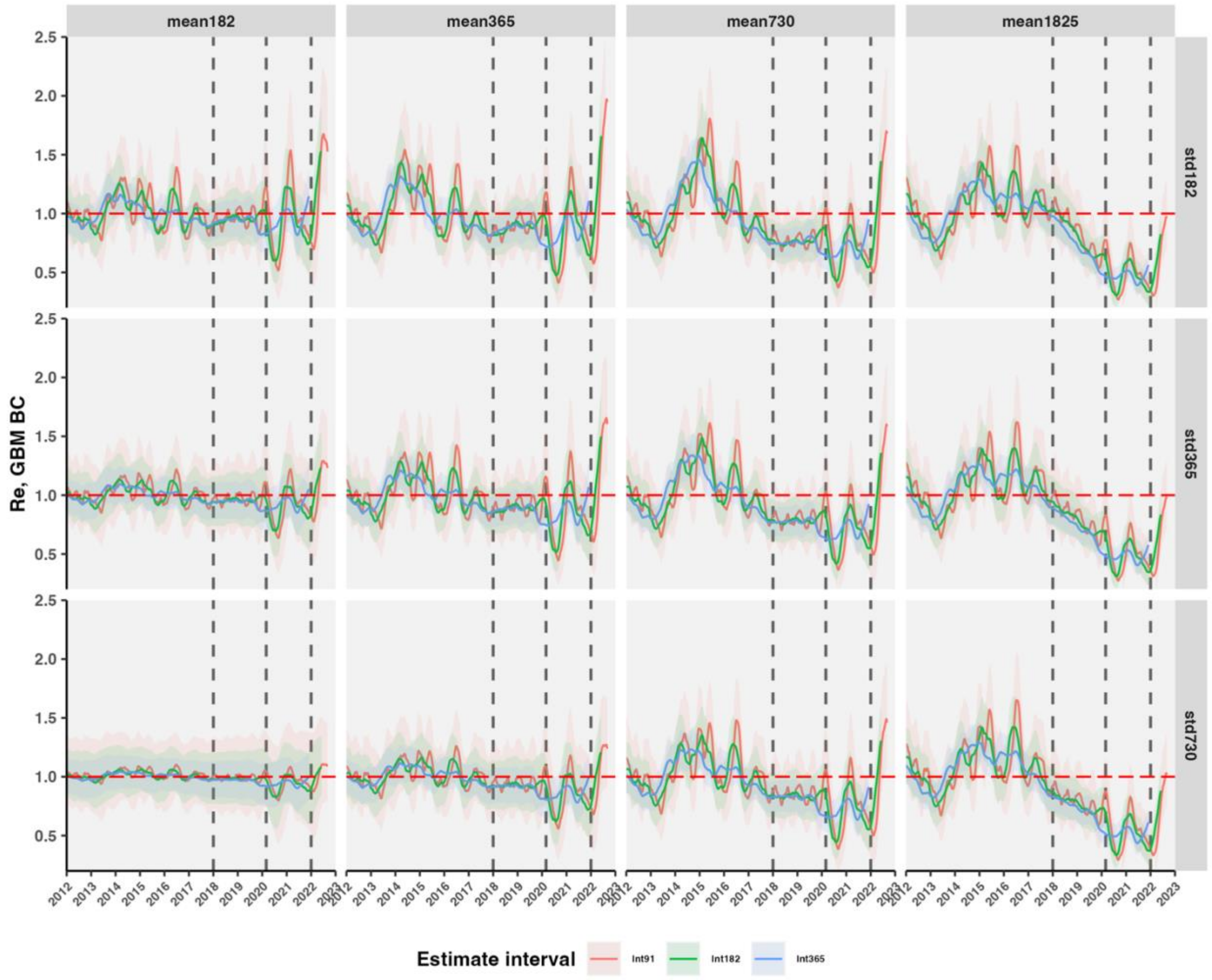

Fig V. HIV R_e among GBM in BC from 2012 to 2022 under multiple serial interval assumptions. R_e estimated with gamma distributed serial intervals for multiple parameters with means (by columns) of 0.5 y (182 d), 1 y (365 d), 2 y (730 d), or 5 y (1825 d); standard deviations (by rows) of 0.5 y, 1 y, or 2 y; and estimate intervals (by color) of 0.25 y (91 d), 0.5 y, or 1 y. R_e were smoothed with k=90 d, right-aligned.

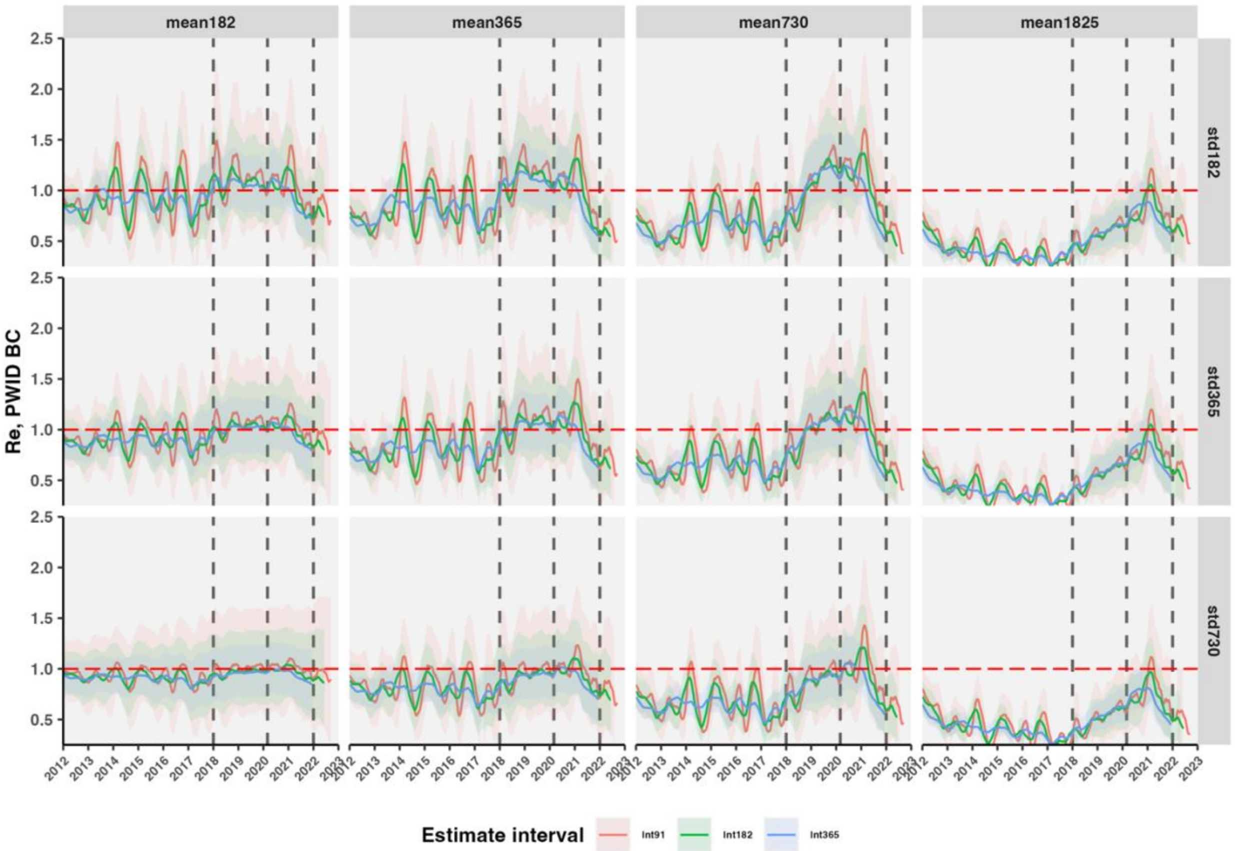

Fig W. HIV R_e among PWID in BC from 2012 to 2022 under multiple serial interval assumptions. R_e was estimated with gamma distributed serial intervals for multiple parameters with means (by columns) of 0.5 y (182 d), 1 y (365 d), 2 y (730 d), or 5 y (1825 d); standard deviations (by rows) of 0.5 y, 1 y, or 2 y; and estimate intervals (by color) of 0.25 y (91 d), 0.5 y, or 1 y. R_e were smoothed by 90 d, right-aligned.

Longer mean serial intervals up to five years were associated with R_e further from 1, but are unrealistic in recent years where most individuals get diagnosed and rendered untransmissible with antiretroviral treatment. Wider standard deviation of the serial interval gamma distribution brought R_e closer to 1 (Fig R-23). Lower mean serial intervals were associated with wider confidence intervals. In our primary analysis, we report R_e estimated with intermediate values: gamma-distributed serial interval with mean 1 y, standard deviation 1 y, and R_e estimate interval of 0.5 y, and 90 d smoothing. Temporal trends in the R_e inflection points were similar for multiple smoothing windows (Figs T-V).

Phylogenetic cluster-specific R_e

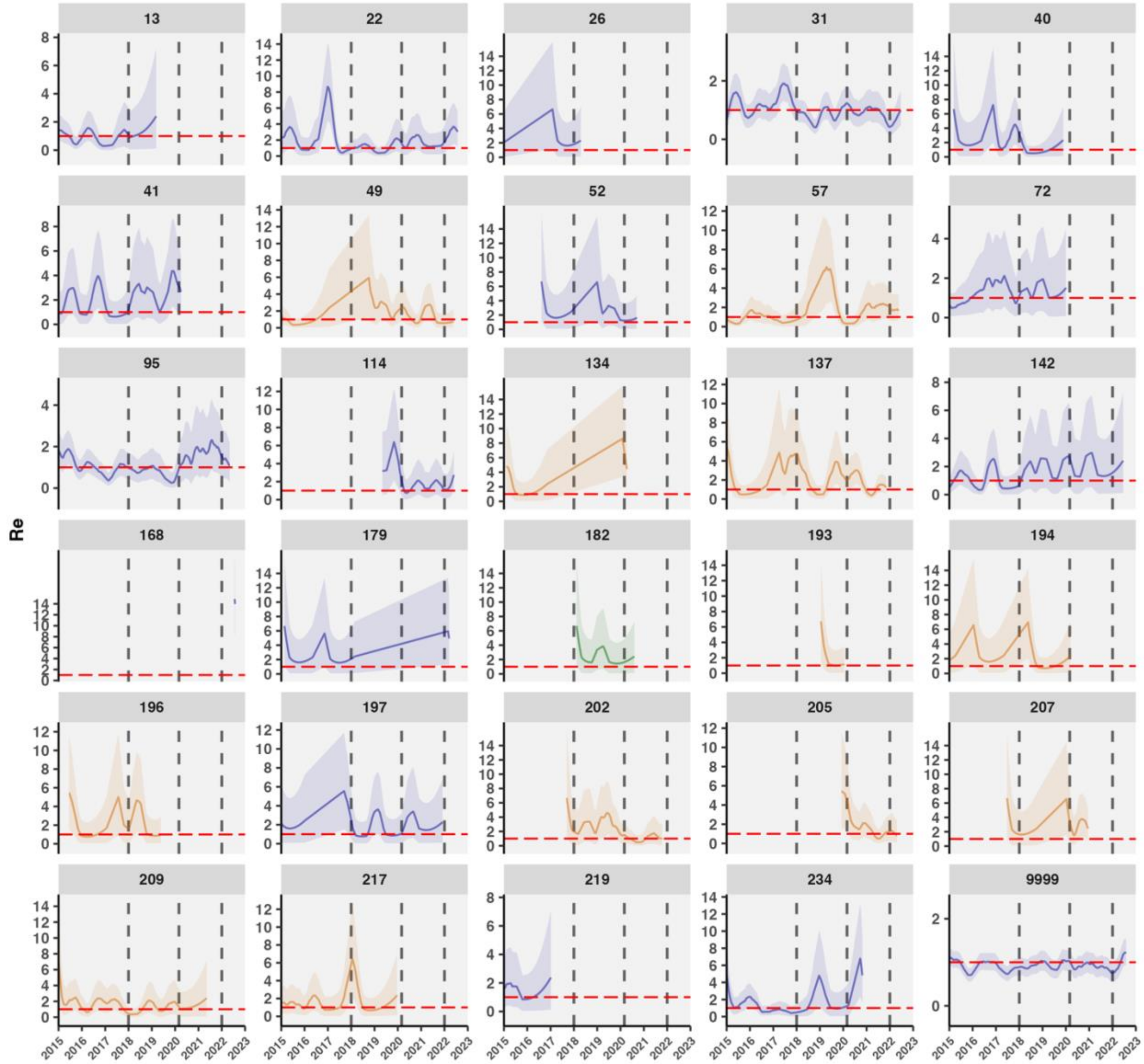

Fig X. HIV R_e across large (size in 2022>9) and active (new cases since 2018>2) clusters, estimated with gamma-distributed serial interval mean 1 y, standard deviation (sd) 0.5 y, estimating window 0.5 y, and 90 d smoothing. Excludes values where the confidence interval width exceeded 10 due to sparse cases. Cluster 9999 is all non-clustered cases. Color denotes predominant population (blue: GBM, orange: PWID, and green: HET).

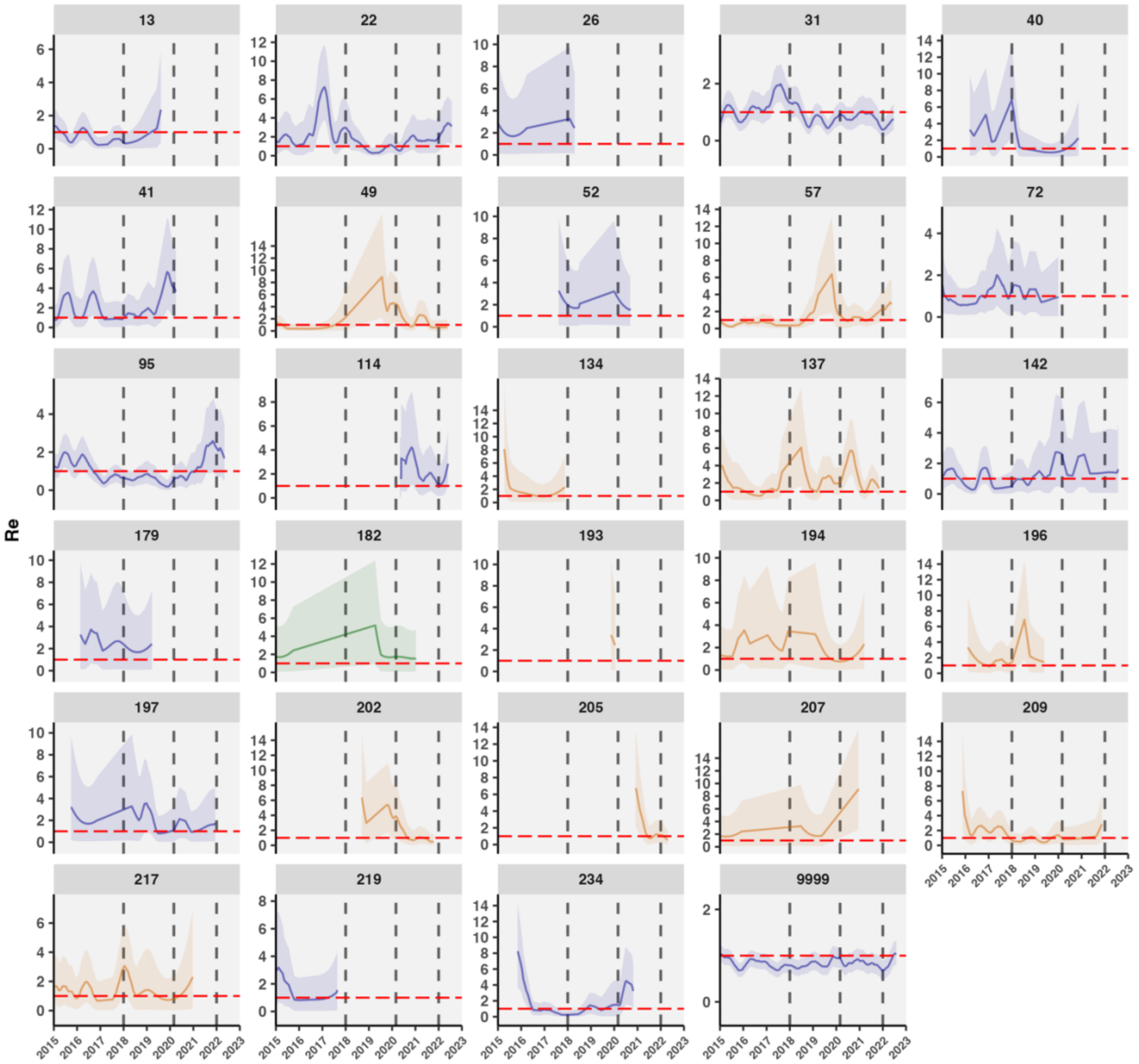

Fig Y. HIV R_e across large and active clusters, estimated with gamma-distributed serial interval *mean 2 y*, sd 0.5 y, estimating window 0.5 y, and 90 d smoothing.

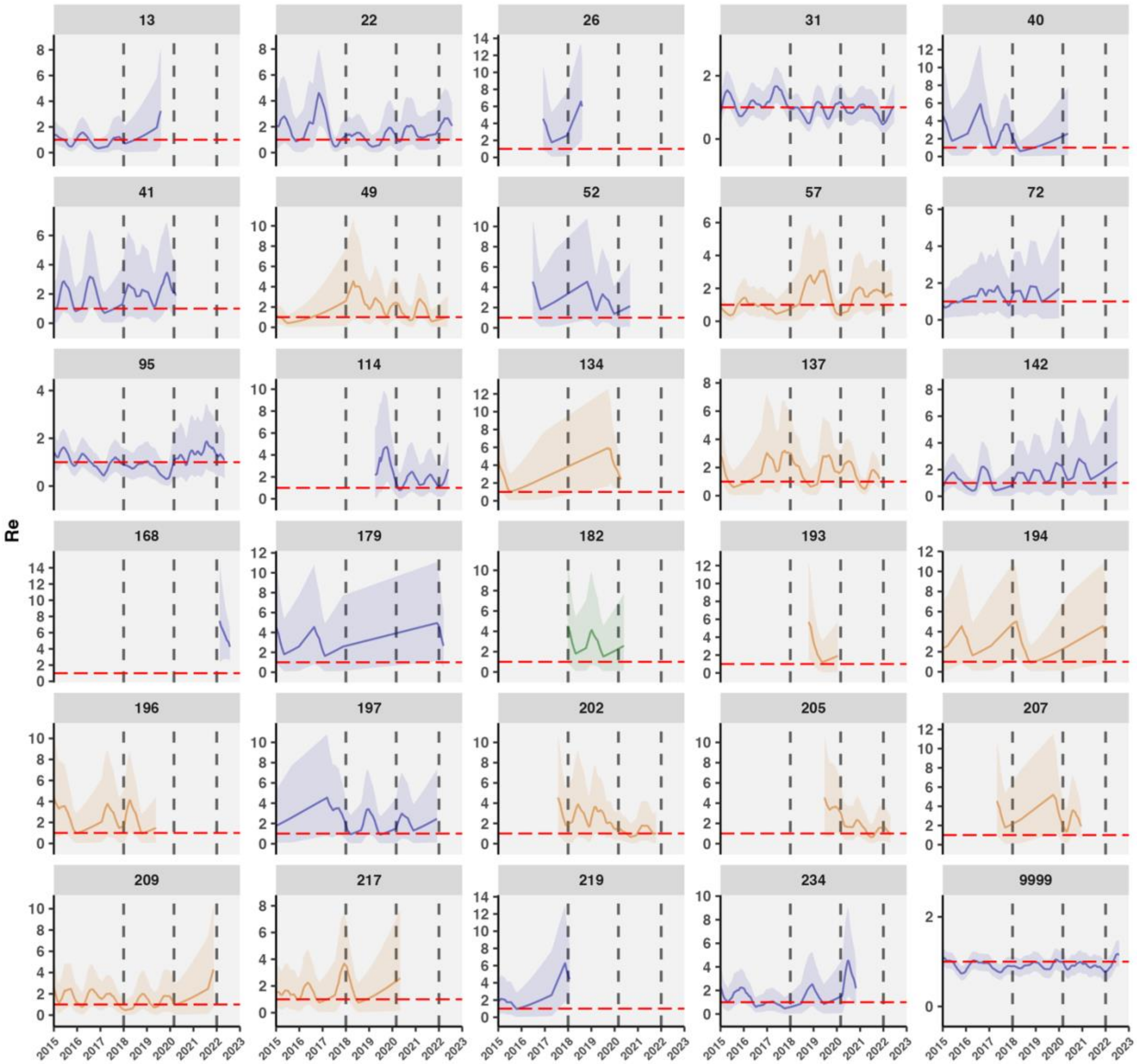

Fig Z. HIV R_e across large and active clusters, estimated with gamma-distributed serial interval mean 1 y, *sd 1 y*, estimating window 0.5 y, and 90 d smoothing.

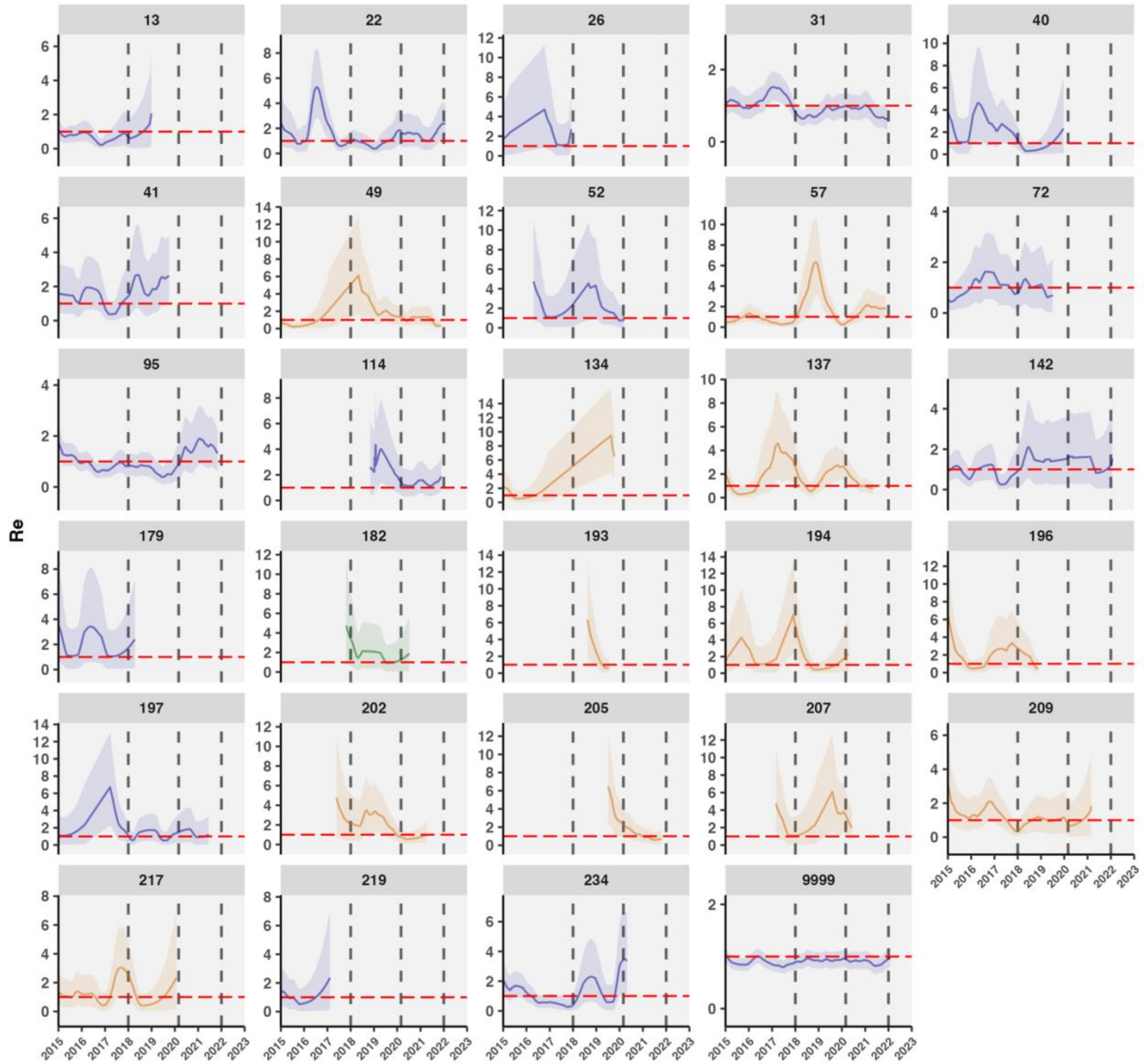

Fig AA. HIV R_e across large and active clusters, estimated with gamma-distributed serial interval mean 1 y, sd 0.5 y, *estimating window 1 y*, and 90 d smoothing.

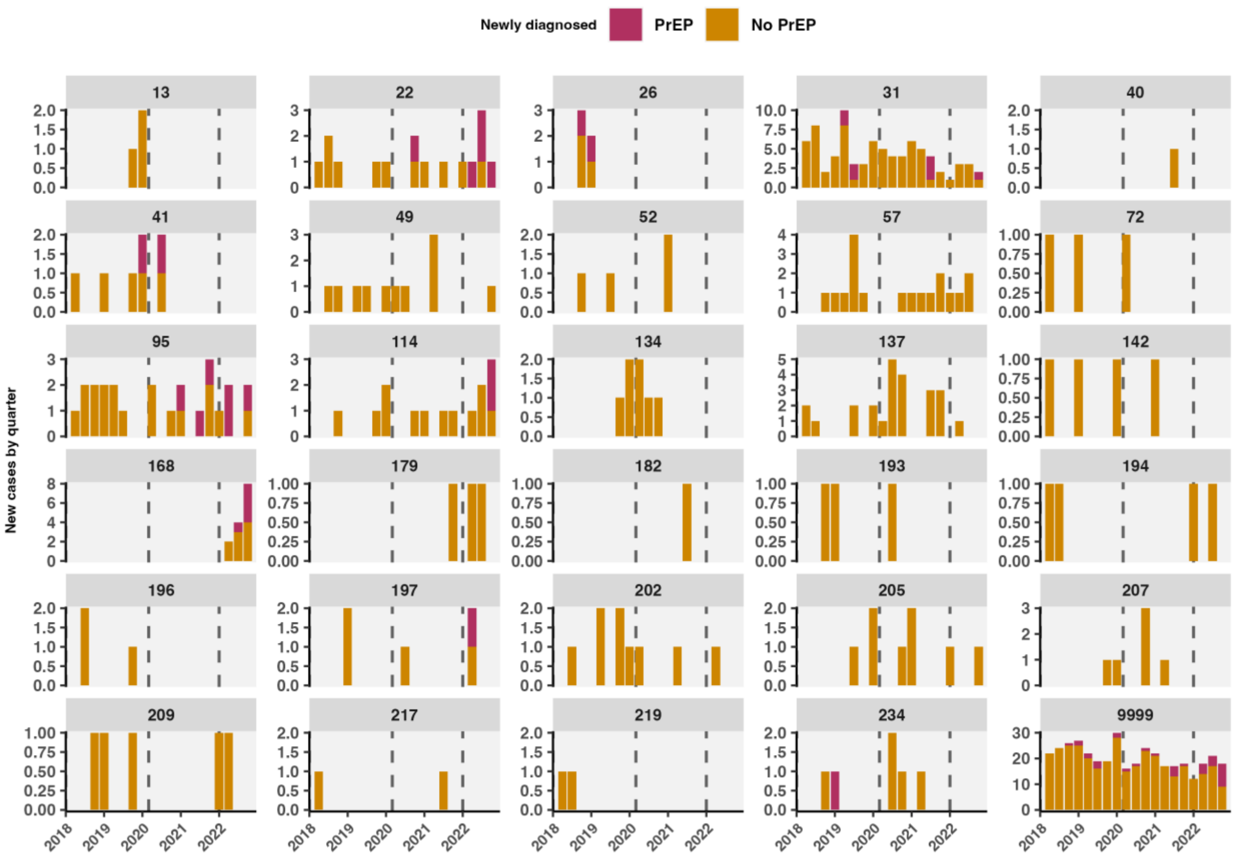

Fig AB. Quarterly new cases in large active clusters by PrEP use. Cluster 9999 represents non-clustered cases.

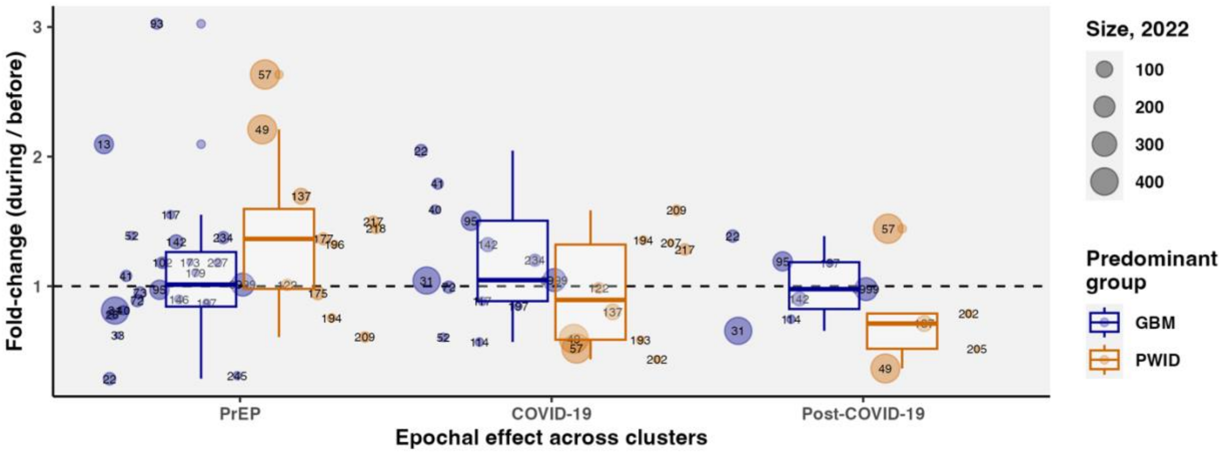

Fig AC. Epochal PrEP effect and COVID-19 effect on clusters' R_e. Epochal effects were calculated as fold-change in the piecewise average R_e in the PrEP period, compared to before PrEP (during PrEP/pre-COVID, Jan. 2018 – Feb. 2020 vs. before PrEP, Jan. 2016 – Dec. 2017), COVID-19 (during COVID, Mar. 2020 – Dec. 2021 vs. during PrEP/pre-COVID), and post-COVID-19 (Jan. – Dec. 2022 vs. during COVID). Point size represents cluster size in 2022, color is predominant population. Restricted to medium (>=10 size), active (>=1 case since 2018) clusters. Box plots show median and interquartile range of predominant groups' epochal effects.

Counterfactual simulations of cluster growth

Table F. Adjusting clusters' simulation seed size (number of infectious individuals at time zero) based on ratio of new samples simulated versus observed in an initial simulation. Initial seed was estimated as clusters' cases minus deaths and emigrations at the end of 2017, multiplied by the proportion of diagnosed cases virally unsuppressed, based on estimates from 2019 that 82% of cases in BC were diagnosed, 76% of those diagnosed were on ART, and 83% of those on ART were virally suppressed [46]. Seeds were calibrated to align observed new cases in each cluster from 2018 to end of 2022, with the mean number of sampled new cases from simulations using observed cluster R_e (Table F, Fig V).Active clusters shown. Cluster 9999 represents non-clustered individuals.

Cluster ID	Initial seed	N new samples observed	Mean simulated N new samples (observed R_e)	Adjust factor	Adjusted seed
9999	116	439	205	0.4669	249
57	17	19	8	0.4210	41
31	5	94	16	0.1702	30
49	17	11	18	1.6363	11
202	1	9	1	0.1111	10
168	1	17	2	0.1176	9
207	1	6	1	0.1667	6
197	1	5	1	0.2	5
40	1	3	1	0.3333	4
52	1	4	1	0.25	4
134	1	7	2	0.2857	4
196	1	3	1	0.3333	4
95	2	27	18	0.6667	3
137	3	26	28	1.0769	3
13	3	3	8	2.6667	2
26	1	5	4	0.8	2
142	2	6	8	1.3333	2
182	1	3	2	0.6667	2
201	3	2	4	2	2
209	1	5	3	0.6	2
217	1	4	3	0.75	2
219	1	4	2	0.5	2
234	1	6	4	0.6667	2
194	1	5	4	0.8	2
114	1	14	9	0.6428	2
205	1	9	5	0.5555	2
14	1	1	2	2	1
22	1	19	24	1.2631	1
29	2	1	3	3	1
33	1	1	4	4	1
35	1	2	2	1	1
41	1	7	10	1.4285	1

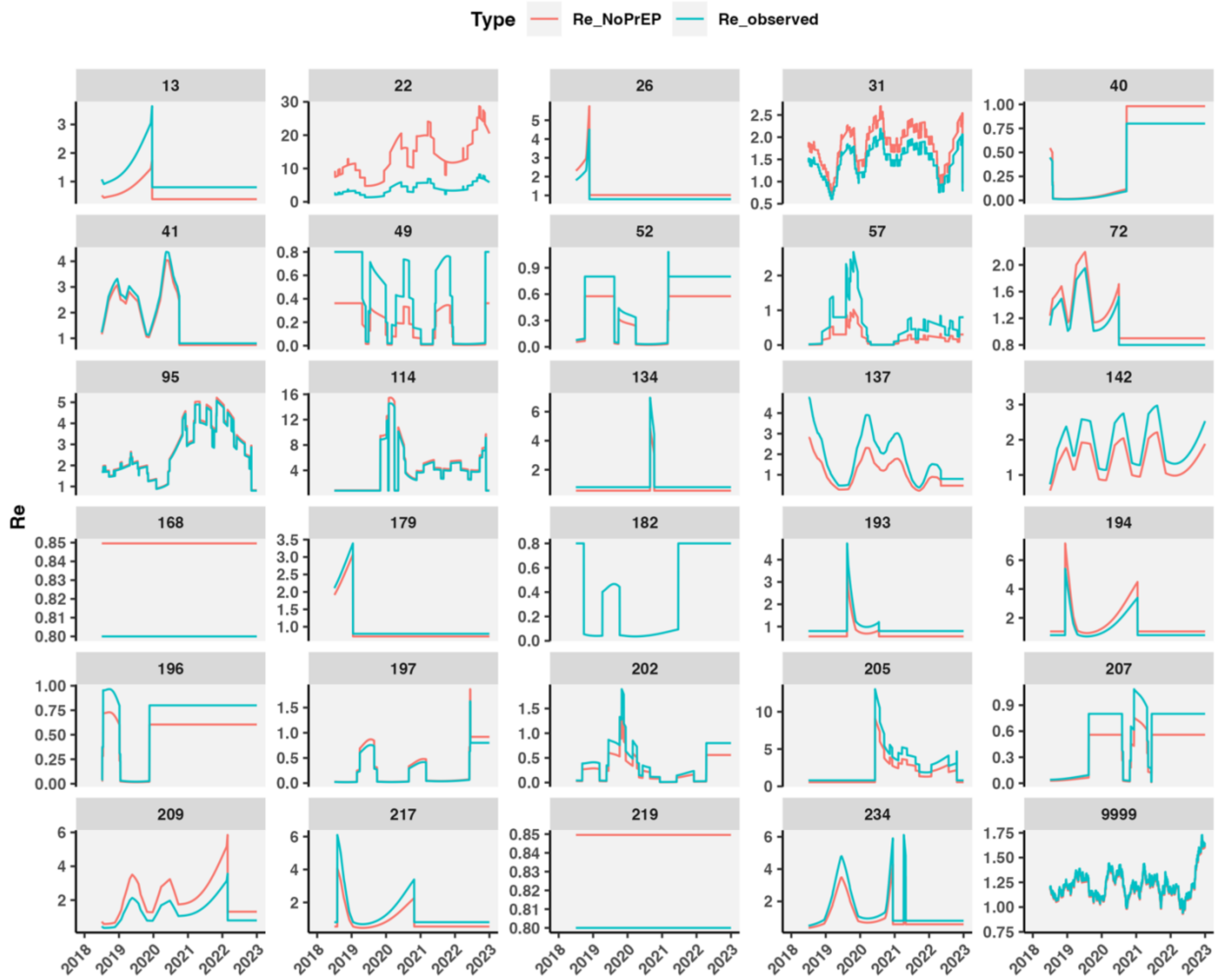

Fig AD. Observed and adjusted (in the absence of PrEP) cluster R_e. Includes clusters with at least one new case since 2018 and size of at least 10 in January 2023.

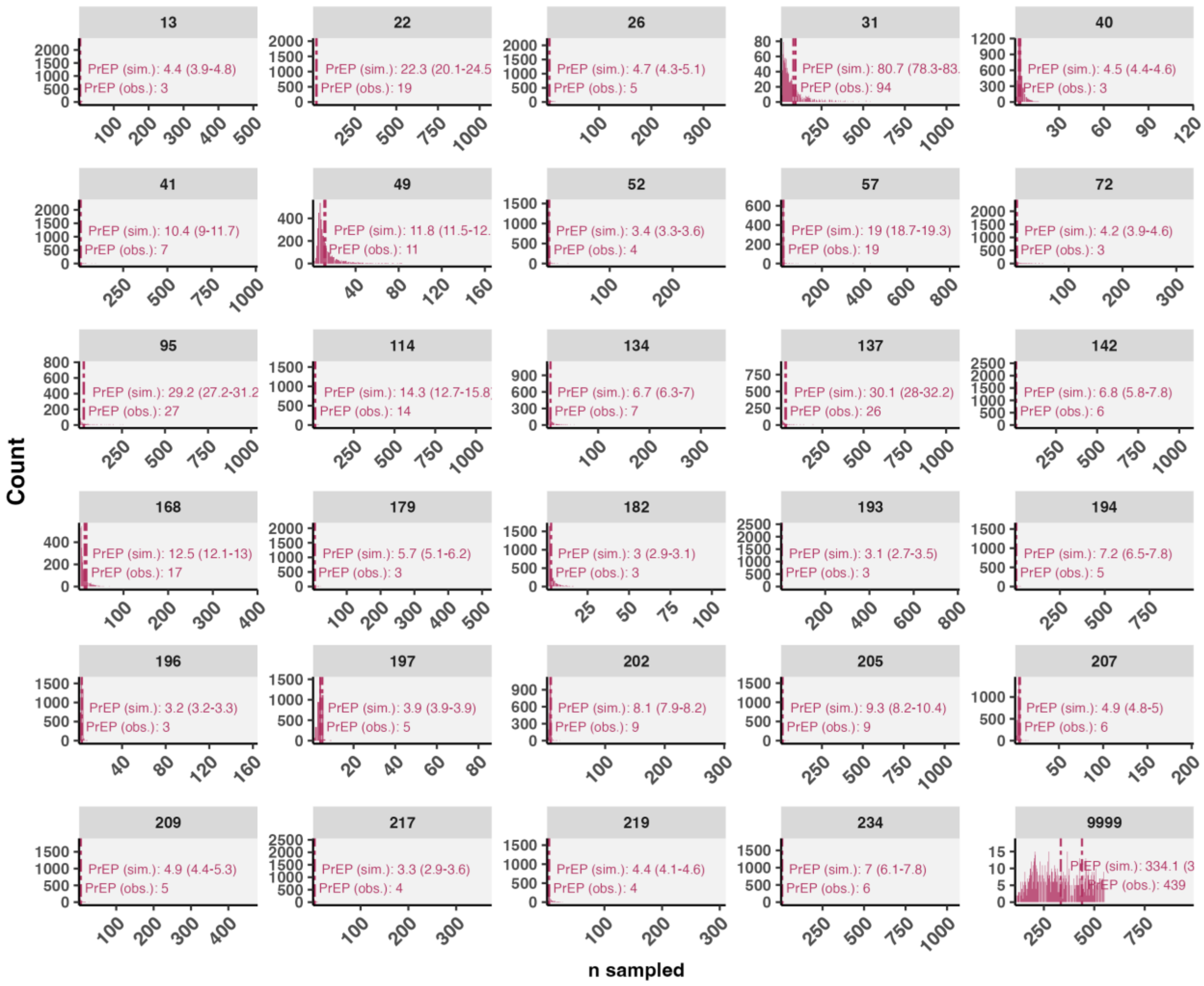

Fig AE. Stochastic branching processes recapitulate observed cluster growth after adjusting seed size. Observed number of new samples ‘PrEP (obs.)’ in large, active clusters from 2018 to 2022 compared to distribution of new samples across 2000 simulations using clusters’ observed R_e ‘PrEP (sim.)’.

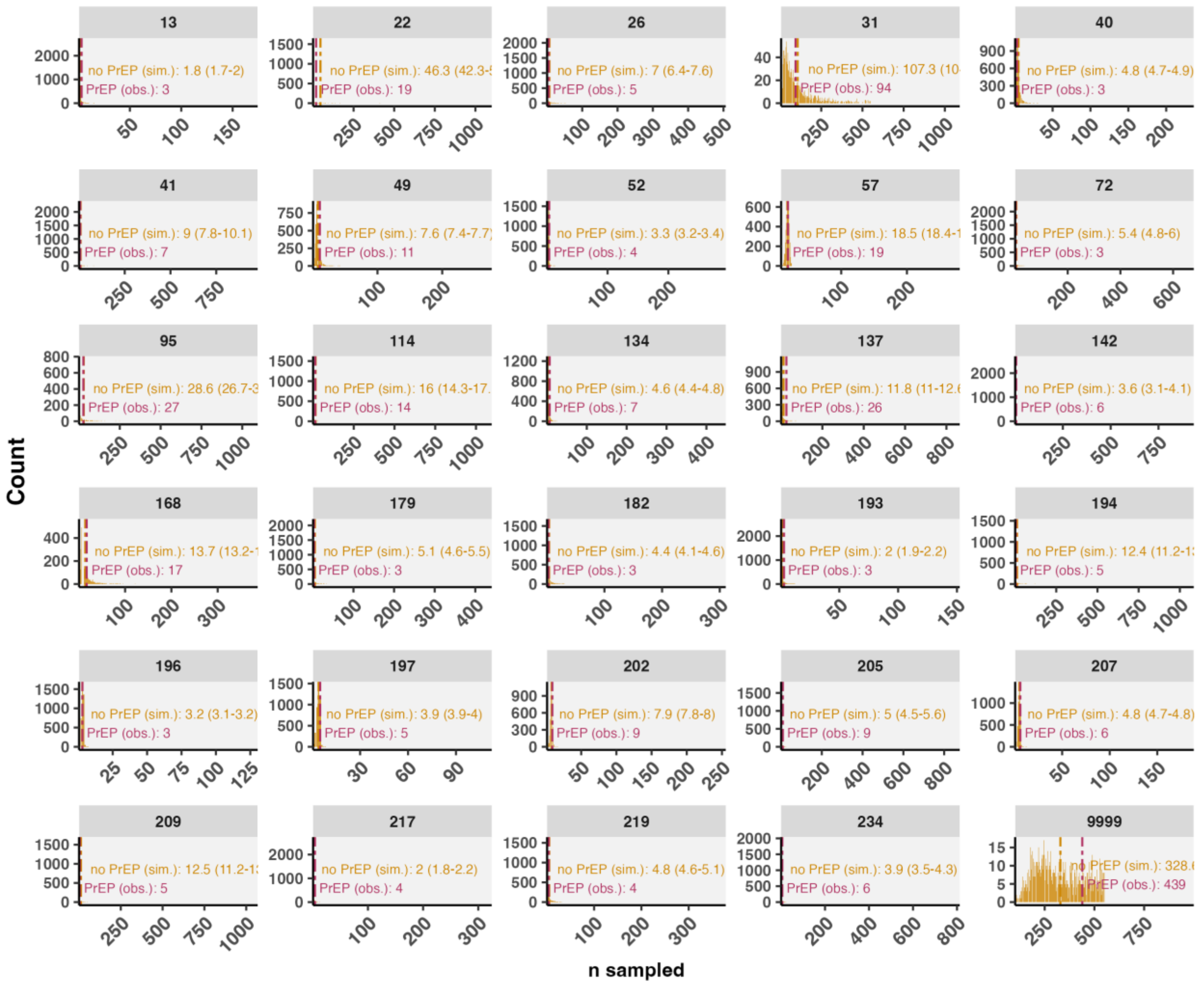

Fig AF. Simulations of cluster samples in the absence of PrEP. Observed number of new samples (among new diagnoses) ‘PrEP (obs.)’ in large, active clusters from 2018 to 2022 compared to distribution of new samples across 4000 simulations using clusters R_e adjusted by PrEP effect ‘no PrEP (sim.)’.

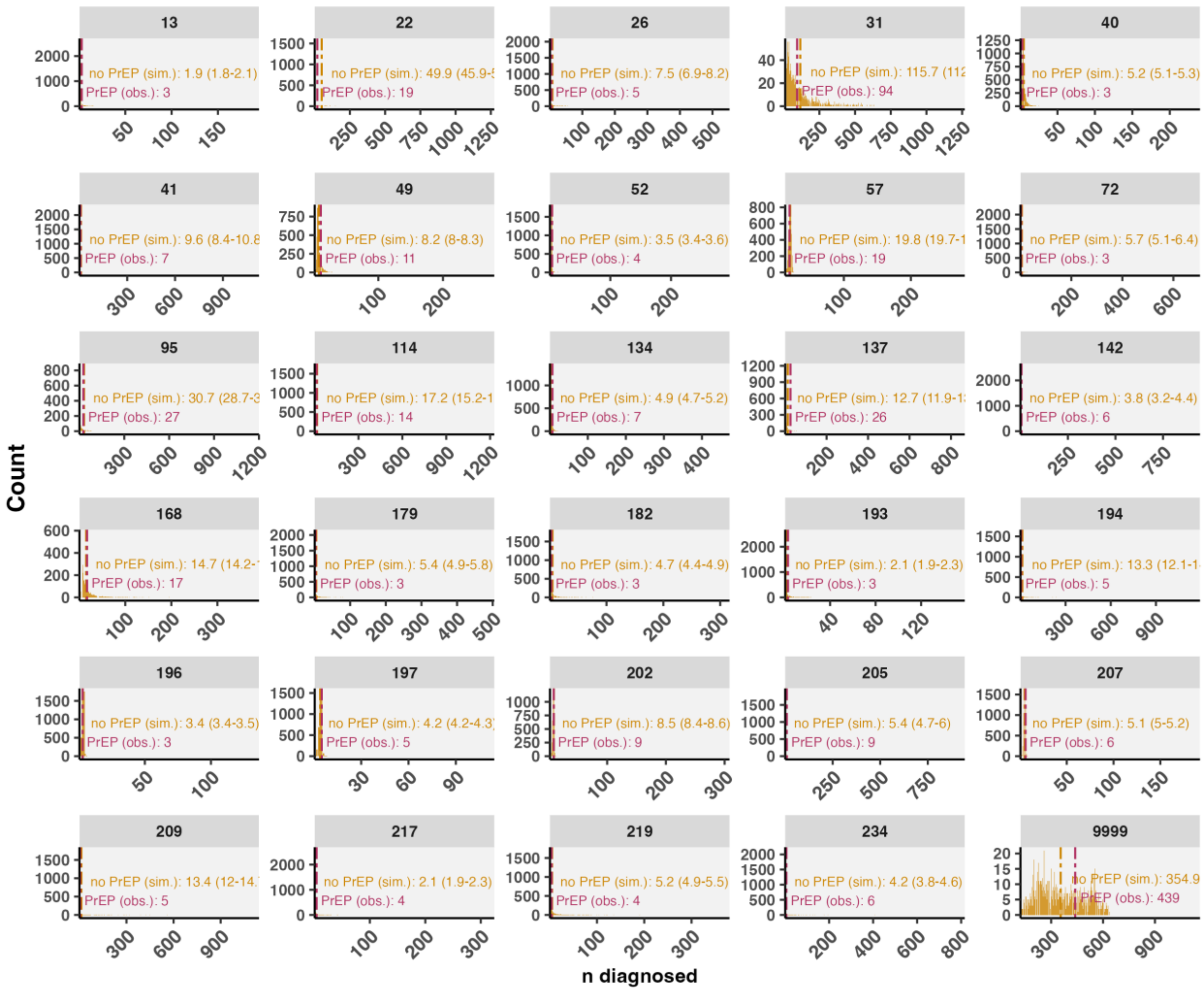

Fig AG. Simulations of cluster diagnoses in the absence of PrEP. Observed number of new diagnoses 'PrEP (obs.)' in large, active clusters from 2018 to 2022 compared to distribution of new diagnoses across 4000 simulations using clusters R_e adjusted by PrEP effect 'no PrEP (sim.)'.

Table G. Parameters of stochastic branching process simulations were specified based on the literature, observed or estimated at the cluster-level. Parameters were grouped into types by whether they were specified based on literature, observed (trends) at cluster-level, estimated at cluster-level in simulations. Standard deviation (sd). Uncertainty in R_e estimation was explored for variables marked **.

Type	Parameter	Range	Specified or output	References
**Specified based on literature**	Dispersion k	**k=0.1-0.3	Specified in simulations: UNIF(k)	Althaus 2015; Riou & Althaus 2020; Lloyd-Smith et al. 2005
	Serial interval	**Mean=0.5-5 y<br>**sd=0.5-2 y	Specified to estimate Re and in simulations: Gamma(mean, std)	Hollingsworth et al. 2008, 2015; Peruski et al. 2021
	P(diagnosed|case)	82%	Specified to estimate initial seed size, convert estimated N cases to N diagnoses	Lima et al. 2017
	P(ART|diagnosed)	76%	Specified to estimate initial seed size	
	P(virally suppressed|ART)	83%	Specified to estimate initial seed size	
**Observed, cluster-level**	Instantaneous effective reproduction number (Re)	**Estimating interval (0.25, 0.5, 1 y)<br>**Serial interval ~ Gamma(mean 0.5-5 y, sd 0.5-2 y)<br>**Smoothing interval (30 d, 90 d, 365 d)	Specified for simulations: ~ NegBin(mean=Re, disp=k);<br>Period ave. Re (pre/during PrEP;pre/during/post COVID-19) used to calculate PrEP effect	Cori et al. 2013; Fig X-S28, S30
	Observed N diagnoses, 2018-2022	Summarized as rolling average, cumulative	Specified to estimate seed adjust factor and cases averted	Table F
**Observed trend, cluster-level**	PrEP effect (fold-change Re)	Average Re with PrEP pre-COVID / average Re before PrEP	Specified to adjust Re without PrEP	Fig AC
	Adjusted Re without PrEP	Adj Re = Observed Re / PrEP effect	Used in counterfactual simulations	Fig AD
**Estimated, cluster-level**	Initial seed size (n infectious at time zero, Jan 1 2018)	(N cases 2018 – N migrants – N deaths) * (P(not diagnosed|case) + P(no ART|diagnosed) + P(not virally suppressed|ART))	Used in initial simulation	Table F
	Initial simulated N new cases and diagnoses, 2018-2022	Rolling average, cumulative for each cluster and overall for Scenarios 1 and 2	Simulation output	Table F; Fig AE
	Seed adjustment factor	Observed N diagnoses / Initial simulated N diagnoses	Simulation output	Table F
	Adjusted seed size	Initial seed size * seed adjust factor	Simulation output;<br>Used in subsequent simulation	Table F
	Simulated N new cases and diagnoses, 2018-2022	Rolling average, cumulative for each cluster and overall for Scenarios 1 and 2	Simulation output	Fig AF, S33
	Diagnosed cases averted	N diagnoses simulated without PrEP - N diagnoses observed with PrEP	Simulation output	Fig. 6

Table H. Poisson model of diagnoses averted across clusters. Counts were normalized to be positive integers (minimum averted added to all). Exponentiated coefficients reported as mean with lower and upper 95% confidence intervals (CI). Significant adjusted relationships in bold.

	**Mean**	**Lower 95% CI**	**Upper 95% CI**
**(Intercept)**	29.1698	19.1228	44.2284
**log(size2017)**	1.0120	0.9680	1.0569
**% GBM**	**1.0016**	**1.0001**	**1.0031**
**median age, 2023**	**0.9906**	**0.9822**	**0.9991**
**% Van. Coastal**	1.0006	0.9980	1.0033

References

1. Li H. Minimap2: pairwise alignment for nucleotide sequences. Bioinformatics. 2018;34(18):3094–100.

2. Moshiri N. ViralMSA: Massively scalable reference-guided multiple sequence alignment of viral genomes. Bioinformatics. 2020;37(5):714–6.

3. Struck D, Lawyer G, Ternes AM, Schmit JC, Bercoff DP. COMET: adaptive context-based modeling for ultrafast HIV-1 subtype identification. Nucleic Acids Res. 2014;42(18):e144–e144.

4. Pineda-Peña AC, Faria NR, Imbrechts S, Libin P, Abecasis AB, Deforche K, et al. Automated subtyping of HIV-1 genetic sequences for clinical and surveillance purposes: Performance evaluation of the new REGA version 3 and seven other tools. Infect Genetics Evol. 2013;19:337–48.

5. Bennett DE, Camacho RJ, Otelea D, Kuritzkes DR, Fleury H, Kiuchi M, et al. Drug Resistance Mutations for Surveillance of Transmitted HIV-1 Drug-Resistance: 2009 Update. PLoS ONE. 2009;4(3):e4724-8.

6. British Columbia Centre for Excellence in HIV/AIDS. Guidance for the use of pre-exposure prophylaxis (PrEP) for the prevention of HIV acquisition in British Columbia. 2020;

7. Molina JM, Capitant C, Spire B, Pialoux G, Cotte L, Charreau I, et al. On-Demand Preexposure Prophylaxis in Men at High Risk for HIV-1 Infection. New Engl J Medicine. 2015;373(23):2237–46.

8. CATIE. Health Canada approves Apretude, the first long-acting injectable for HIV prevention. 2024. https://www.catie.ca/catie-news/health-canada-approves-apretude-the-first-long-acting-injectable-for-hiv-prevention

9. Toy J. New HIV diagnoses in the setting of publicly funded PrEP in British Columbia. Conference on Retroviruses and Opportunistic Infections 2023 Poster. 2023.

10. Toy J, Espinoza R, Trigg J, Shen T, Sereda P, Ready E, et al. Real-world utilization of HIV PrEP medication in a population-level PrEP program in British Columbia, Canada. Conference on Retroviruses and Opportunistic Infections 2024 Poster. 2024.

11. Misra K, Huang JS, Udeagu CCN, Forgione L, Xia Q, Torian LV. Pre-exposure prophylaxis (PrEP) use history in people with antiretroviral resistance at HIV diagnosis: Findings from New York City HIV surveillance and partner services, 2015-2022. Clin Infect Dis. 2023;ciad699.

12. Jetz W, Thomas GH, Joy JB, Hartmann K, Mooers AO. The global diversity of birds in space and time. Nature. 2012;491(7424):444–8.

13. Rambaut A, Lam TT, Carvalho LM, Pybus OG. Exploring the temporal structure of heterochronous sequences using TempEst (formerly Path-O-Gen). Virus Evol. 2016;2(1):vew007-7.

14. Statistics Canada. Table 17-10-0009-01 Population estimates, quarterly. 2023 [cited 2023 Nov 20]. https://www150.statcan.gc.ca/t1/tbl1/en/tv.action?pid=1710000901
