# Supplementary material for: Heterogeneous impacts of HIV pre-exposure prophylaxis (PrEP) on drug resistance and phylogenetic cluster transmission dynamics in British Columbia, Canada: A retrospective cohort and simulation study
Source: PLoS Med. 2025 Dec 9;22(12):e1004827. doi: 10.1371/journal.pmed.1004827 (PMC12700434; doi:10.1371/journal.pmed.1004827)
Supplement: S3 File — (PDF) [file pmed.1004827.s003.pdf]

ETHICS PROTOCOL
October 7, 2020

Evaluation of HIV phylogenetic cluster growth dynamics following provision of pre-exposure prophylaxis

Principal Investigators: Dr. Jeffrey Joy
Additional Researchers: Angela McLaughlin, Julio SG Montaner

Background

Although British Columbia (BC) has experienced a reduced HIV incidence in recent years[1], ongoing HIV transmission disproportionately affects sub-populations at risk. Connecting these sub-populations, and clusters within sub-populations, with prioritized treatment and prevention is critical to both controlling and working towards ending the HIV epidemic.

BC has been a global leader in demonstrating the effectiveness of highly active antiretroviral therapy (HAART) in reducing HIV morbidity and mortality[2,3], treatment as prevention (TaSP)[4,5], safe injection sites[6], and most recently, pre-exposure prophylaxis (PrEP)[7]. Although PrEP uptake increased rapidly up to 5,653 participants in the 24-months since its introduction in January 2018[8,9], the full extent of its effect on HIV transmission has not been fully characterized. In a mathematical model of HIV infection dynamics, Irvine *et al.* identified HIV risk behavior and interest in PrEP was associated with the population-level effectiveness of PrEP[10]. We expect sub-populations in the community to differ in their patterns of risk behavior, PrEP interest, and PrEP usage, begging the question of how HIV transmission dynamics have changed in these communities since the adoption of PrEP. A better understanding of PrEP effectiveness at a population-level has implications for program optimization within BC, as well as informing other jurisdictions of best practices.

The application of Bayesian birth-death skyline phylodynamic modelling lends itself well to estimating epidemiological parameters, such as the basic reproductive number (R_0), as it jointly infers the phylogeny and the parameters with an underlying susceptible-infected-removed (SIR) model[11]. Coalescent models are unsuitable for cluster-specific analyses as they are well-sampled epidemics, negating the assumption of sparse sampling from a large population[11,12]. While mathematical models can also be applied to estimate R_0, these models do not consider the evolutionary history stamped into viral genetic sequences and thus recover R_0 with limited granularity.

Phylogenetic clustering is routinely applied for real-time HIV surveillance in BC[13] and globally[14,15]. Commonly applied methods, which primarily differ in computing either genetic or tree distance and in the cluster membership threshold they apply, can generate different results and can be sensitive to sampling bias[16]. By analyzing clusters in a phylodynamic framework, the sampling proportion is estimated jointly with the transmission and recovery rates, as well as substitution and evolutionary models, which accounts for differences in cluster sampling that may otherwise be obscured.

We endeavor to apply phylodynamic models to compare cluster-specific estimates of the reproductive number, period of infectiousness, and sampling proportion over time to illuminate the potentially differential effectiveness of PrEP and other public health interventions. In a preliminary analysis of the four largest HIV phylogenetic clusters in BC at the beginning of 2019, we found that R_0 was notably different between the biggest MSM clusters in recent years. Through methods refinements, updated data, and linkage to data on PrEP uptake and seroconversions, we expect to see significantly different

reductions in cluster-specific R_0 following the widespread availability of PrEP, based on systematic differences in PrEP usage and adherence preceding seroconversion. If any of the few (12/~6000) PrEP users who seroconverted also joined clusters, we will evaluate whether they joined clusters with R_0 above 1, and whether there were any factors (sociodemographic or PrEP usage-related, ie prescription refills) associated with cluster membership among seroconverted PrEP users. It is additionally of interest to evaluate whether there are signature patterns of drug resistance mutations among viruses from seroconverted PrEP users. Longitudinal samples combined with phylogenetics may allow us to distinguish transmitted from acquired resistance in this sub-cohort.

These findings have direct implications for improving availability and consistent use of PrEP for individuals who are contacts of clusters that have a growing R_0. Further, if we find an association between particular drug resistance mutations and PrEP seroconversion, this may inform drug regimens and underline the importance of PrEP users getting tested often.

Purpose

To evaluate cluster-specific changes in reproduction number and other epidemiological parameters over time, specifically in response to the availability of PrEP. Further, to investigate factors associated with both clustering and drug resistance among PrEP users who seroconverted.

Hypotheses

1. In alignment with a decreasing trend in HIV incidence in BC over time, we hypothesize that the province-wide R_0 will be shown to steadily decrease over time in response to HAART as well as PrEP.
2. We hypothesize that if PrEP has played a strong role in reducing HIV transmission amongst young MSM that cluster-specific estimates of R_0 for young MSM clusters will reveal a reduction in R_0 following the advent of free widely-available PrEP. Alternatively, if PrEP has not played a key role then cluster-specific estimates of R_0 will not decline concomitantly with PrEP introduction.
3. There are significant differences in cluster-specific estimates of R_0, sampling proportion, and period of infectiousness over time, where at least one cluster maintained an R_0 above 1 following the widespread availability of PrEP.
4. PrEP users who seroconverted were more likely to harbor drug resistance mutations compared to non-PrEP users who seroconverted.
5. PrEP users who seroconverted and joined a phylogenetic cluster (vs. did not join a cluster) were more likely to be young and living in the Vancouver Coastal health authority.
6. PrEP users who seroconverted and joined a phylogenetic cluster tended to join a cluster with a reproduction number greater than 1 in the preceding year.

Objectives

1. To estimate the reproduction number over time for all of BC and to correlate any changes with public health interventions (ie HAART, needle exchange, safe-injection site, PrEP).
2. To compare phylogenetic cluster-specific estimates of the reproduction number over time using phylodynamic methods and to associate changes with public health interventions.
3. To evaluate drug resistance mutation patterns in PrEP users who seroconverted and to compare these patterns to all those who seroconverted in a multiple regression framework.
4. To evaluate sociodemographic and clinical factors associated with cluster membership among seroconverted PrEP users.
5. To identify which clusters seroconverted PrEP users joined and confirm whether these are the clusters that have not experienced as marked reductions in Ro.

<u>Research procedures</u>

Dataset

We propose to re-analyze a de-identified dataset representing participants in the BC-CfE Drug Treatment Program (DTP) up until the end of 2020. We will analyze all available HIV sequences (36271 partial pol sequences from 9630 PLHIV in February 2019) generated by the BC-CfE clinical HIV resistance genotype test. We will connect HIV sequences with de-identified participants' sociodemographic and clinical information centralized in the DTP including sex, age, ethnicity, census tract of patient residence, physician census tract, date of seroconversion, date of first antiretroviral, treatment history, PrEP history, risk factors (men who have sex with men, MSM; people who inject drugs, PWID; heterosexual activity; blood exposure; previous HCV co-infection; other), plasma viral RNA loads, CD4+ T cell count, having ever had acquired immune deficiency syndrome (AIDS); if applicable date of mortality and cause of death.

To maintain confidentiality and privacy, all patient data will be de-identified and all sequences will be assigned random six-character identifiers. The extraction, PCR, and sequencing of plasma HIV RNA was performed by clinical staff at the BC-CfE and genotypic data were subsequently stored at the BC-CfE access-controlled facility in a secure, encrypted, password protected Oracle database. Investigators (including co-investigators) will have no access to nominal data. Access to nominal data is restricted within the BC-CfE's DTP Clinical Registry exclusively to a select few dedicated DTP personnel (Senior Oracle programmers and Senior Data Analysts), who have access to identifiable data in the secure Oracle server or to the key that links to that data.

Phylogenetic clustering

Sequences will be aligned to the HXB2 reference genome (GenBank Accession #K03455) using MAFFT. Insertions and deletions relative to HXB2, as well as amino acids corresponding to WHO recognized drug resistance mutation sites, will be removed from the alignment for tree inference, however their presence will be noted as categorical variables. A set of shuffled bootstrap alignments will be generated to infer 100 approximate maximum likelihood phylogenetic trees, as implemented in FastTree2.1. Alternative tree-building software, ie IQ-TREE, will be compared using topological metrics and bootstrap support values.

We will then use the bootstrap trees to identify phylogenetic clusters using the standard methodology applied for HIV public health cluster surveillance. Specifically, we will compute pairwise patristic (tree) distance threshold of 0.02 substitutions per site for every bootstrap, identify pairs of nodes supported by >90% bootstraps, and then group nodes into clusters with 5+ members. We will evaluate different tree distance and bootstrap support thresholds, as well as a TN93 (HIV-TRACE) methodology.

By iterating this method of alignment, tree-building, and cluster identification over a longitudinally down-sampled dataset (to effectively blind ourselves to new data to mimic past inference back to 1996), we will model cluster growth over time, without accounting for sampling differences.

Phylodynamic inference of epidemiological parameters

We will stratify the dataset into cluster-specific HIV sequence alignments with the oldest sample from each patient. For all clusters, we will separately apply Bayesian birth-death skyline modelling using the BEAST2 software to estimate the reproductive number, sampling proportion, and period of infectiousness over time. If there is adequate evidence for population structure (by age group or geography) in a given cluster, then a multi-type birth-death (mtbd) model will be instead assumed. This model structure additionally parameterizes the viral migration rate between sub-populations. Other priors, such as the site model and molecular clock, will be justified by robust model testing and/or averaging.

Hypothesis testing

We will investigate changes in R_0 after the widespread availability of PrEP between clusters using a non-parametric Kruskal-Wallis test. To test whether there was a reduction in R_0 following PrEP for each cluster, we will build a generalized linear model, where each observation is a cluster and the outcome is R_0. We will use Bayesian stochastic search variable selection to identify variables to include in the model and estimate their coefficient. We will consider cluster-aggregated variables including but not limited to median age, % MSM, % PrEP use among new cases in cluster, aggregated diversification rate and viral load metrics, and % Vancouver Coastal. For comparison, the outcome of cluster growth rates could also be calculated using the number of confirmed cases in past year (normalized by cluster size).

We are additionally interested in confirming whether the clusters with higher R_0 were joined by seroconverted PrEP users. As there were only 12 known seroconverters who had used PrEP in BC (by early 2020), we would likely be underpowered to make a model with the outcome of whether or not seroconverted PrEP users joined a cluster. However, we will evaluate any trends in participant characteristics among clustering seroconverted PrEP users.

Finally, we will compare drug resistance patterns (individual mutations and combinations) among PrEP users who seroconverted to all others who seroconverted in a multiple logistic regression framework, adjusting the p-value for multiple comparisons. It is possible that we will be underpowered to make any conclusions about the effect of PrEP. We will try to distinguish transmitted from acquired drug resistance on a case-by-case basis, taking into consideration the appearance of mutations in later samples only, the estimated delay in diagnosis (initial CD4 count and viral load can be informative), whether they were using PrEP over that time period, and whether the phylogeny suggests that the virus' closest relative shared the drug resistance pattern.

Expected research outcomes

By studying the transmission dynamics of the few remaining active clusters in the BC HIV epidemic in response to PrEP uptake, we hope to further corroborate the importance of PrEP in reducing new infections in sub-populations at risk within Canada and abroad.

References

1. BC Centre for Disease Control. HIV in British Columbia: Annual Surveillance Report 2017. Retrieved from http://www.bccdc.ca/health-professionals/data-reports/hiv-aids-reports. 1–46 (2019). Available at: http://www.bccdc.ca/resource-

gallery/Documents/Statistics%20and%20Research/Statistics%20and%20Reports/STI/HIV_Annual_Report_2017_FINAL.pdf. (Accessed: 1st May 2020)
2. Hogg, R. S. *et al.* Antiviral effect of double and triple drug combinations amongst HIV-infected adults: lessons from the implementation of viral load-driven antiretroviral therapy. *AIDS* **12,** 279–284 (1998).
3. Montaner, J. S. G. *et al.* Expansion of HAART coverage is associated with sustained decreases in HIV/AIDS morbidity, mortality and HIV transmission: the 'HIV Treatment as Prevention' experience in a Canadian setting. *PLoS ONE* **9,** e87872 (2014).
4. Montaner, J. S. *et al.* The case for expanding access to highly active antiretroviral therapy to curb the growth of the HIV epidemic. *The Lancet* **368,** 531–536 (2006).
5. Lima, V. D. *et al.* The Impact of Treatment as Prevention on the HIV Epidemic in British Columbia, Canada. *Curr HIV/AIDS Rep* 1–11 (2020). doi:10.1007/s11904-020-00482-6
6. Tyndall, M. W. *et al.* HIV seroprevalence among participants at a Supervised Injection Facility in Vancouver, Canada: implications for prevention, care and treatment. *Harm Reduct J* **3,** 36 (2006).
7. Nathan J Lachowsky, T. L. T. P. S. C. W. J. E. M. H. Community awareness of, use of and attitudes towards HIV pre-exposure prophylaxis (PrEP) among men who have sex with men in Vancouver, Canada: preparing health promotion for a publicly funded PrEP program. 1–7 (2019). doi:10.1071/SH18115
8. Toy, K. J. *et al.* Rapid uptake of HIV pre-exposure prophylaxis (PrEP) in a publicly funded population-based program in British Columbia, Canada. **956,** 1–1 (2019).
9. Keeney, C. L. *et al.* Population-Based HIV Pre-Exposure Prophylaxis (PrEP) in British Columbia (BC): A 24-Month Update on Client Enrolment and Prescriber Participation. in 1–5 (2020).
10. Irvine, M. A. *et al.* Predicting the impact of clustered risk and testing behaviour patterns on the population-level effectiveness of pre-exposure prophylaxis against HIV among gay, bisexual and other men who have sex with men in Greater Vancouver, Canada. *Epidemics* **30,** 100360 (2020).
11. Stadler, T., Kühnert, D., (null), S. B. P. O. & Drummond, A. J. Birth–death skyline plot reveals temporal changes of epidemic spread in HIV and hepatitis C virus (HCV). *PNAS* (2013). doi:10.1073/pnas.1207965110/-/DCSupplemental
12. Volz, E. M., Kosakovsky Pond, S. L., Ward, M. J., Leigh Brown, A. J. & Frost, S. D. W. Phylodynamics of infectious disease epidemics. *Genetics* **183,** 1421–1430 (2009).
13. Poon, A., Gustafson, R., Daly, P., Zerr, L. & Demlow, S. E. Near real-time monitoring of HIV transmission hotspots from routine HIV genotyping: an implementation case study. *The Lancet HIV* **3,** e231–e238 (2016).
14. Wertheim, J. O. *et al.* Growth of HIV-1 Molecular Transmission Clusters in New York City. *J INFECT DIS* **5,** e1000590–11 (2018).
15. Villandre, L. *et al.* Assessment of Overlap of Phylogenetic Transmission Clusters and Communities in Simple Sexual Contact Networks: Applications to HIV-1. *PLoS ONE* **11,** e0148459–18 (2016).
16. Poon, A. F. Y. Impacts and shortcomings of genetic clustering methods for infectious disease outbreaks. *Virus Evolution* **2,** vew031–9 (2016).

ETHICS APPLICATION
H20-02859 Cluster Dynamics (Version 0.1)
Principal Investigator: Jeffrey Joy
1. Principal Investigator & Study Team - Human Ethics [View Form]

1.1. Principal Investigator
Joy Jeffrey Infectious Diseases - jjoy@cfenet.ubc.ca Med

Enter Principal Investigator’s secondary appointments or affiliations (including Health Authorities), if applicable:

Is there space to enter a co-PI in the form? If so, move Dr. Montaner up here.

1.2. Primary Contact
Last Name First Name Rank
Jackson Jill Research Assistant

1.3A. Co-Investigators - Online Access
Last Name First Institution/Department Name

UBC/Science, Faculty of/McLaughlin Angela/Bioinformatics

Rank Graduate Student

Professor
Montaner
Julio S.G.
UBC/Medicine, Faculty of/Medicine, Department of/Infectious Diseases – Med

1.3B. Describe each Co-I's role in study, e.g. statistician, supervisor, adviser, student etc. Ensure individual is entered in Box 1.3A
Angela McLaughlin is a UBC PhD student working with the principal investigator on the study utilizing her bioinformatics speciality skills.

Julio Montaner is the Executive Director and Physician-in-Chief at the BC Centre for Excellence in HIV/AIDS and will oversee ethical and technical project details.

1.4A. Additional Study Team Members - Online Access
Last Name First Name Institution/Department Rank

1.4B. Describe each Additional Study Team Members' role in study, e.g. staff, research assistant etc.

1.5A. Additional Study Team Members - No Online Access

Last First Institution / Rank / Job Email Name Name Department Title Address

1.5B. Describe each Additional Study Team Members' (no online access) role in study, e.g. external supervisor, consultant etc.
Have all research personnel completed the required TCPS2 tutorial:
Yes

1.7. Project Title Enter the title of this research study as it will appear on the certificate. Title given must match the title on all study documents.
Evaluation of HIV phylogenetic cluster growth dynamics following provision of pre-exposure prophylaxis

1.8. Project Nickname Enter a nickname for this study. What would you like this study to be known as to the Principal Investigator and study team?
Cluster Dynamics

2. Study Dates and Funding - Human Ethics [View Form]
You plan to start collecting data immediately after obtaining ethics and any other required approvals
yes
You plan to start data collection at a later date i.e., 2 months or more after approvals are obtained. Click the calendar icon below to select the dates. Estimated start date:

2.1.B. Estimated end date: 2025-09-08

2.2.A. Types of Funds Please select the applicable box(es) below to indicate the type(s) of funding you are receiving to conduct this research. You must then complete section 2.3 and/or section 2.4 for the name of the source of the funds to be listed on the certificate of approval.

Grant

2.2.B. For Industry Sponsored studies, please provide a sponsor contact.

2.3.A. Research Funding Application/Award Associated with the Study that was Submitted to the UBC Office of Research Ethics
UBC Number Title Sponsor

2.3.B. Which institution is administering the funds, if not UBC or UBC affiliated institution?
2.4.A. Research Funding Application/Award Associated with the Study not listed in question 2.3.
UBC Number
Title Sponsor
Canadian Institutes of Health Research (CIHR)

2.4.B. Please enter any applicable information about your funding which is not already shown in Box 2.3A or 2.4A (including funding applied for but not yet received).
Canadian Institutes of Health Research (CIHR) – Frederick Banting and Charles Best Canada Graduate Scholarships Doctoral (CGSD)

2.5.A. Is this a DHHS grant? No

2.5.B. If yes, please select the appropriate DHHS funding agency from the selection box.
DHHS Sponsor List: Order: Active:

2.6. Study Related Conflict of Interest Conflicts of Interest (COIs) in research are situations where someone's personal interests (financial, career, or other) could compromise or could be perceived to compromise the objective conduct of research or integrity of the data. Conflicts of interest can arise naturally from an Investigator's engagement inside and outside the University, and the mere existence of a COI or the perception of a COI does not necessarily imply wrongdoing on anyone's part. Nonetheless, real and perceived COI must be recognized, disclosed, and assessed. This question asks Investigators to disclose COIs that may relate to the research study that is the subject of the REB application. Do the Principal Investigator, Co-Investigators and/or their related parties have any personal interest(s) that could compromise or reasonably be perceived to compromise the objective conduct of the research or the integrity of the data generated by the study? Personal interests may include business, commercial or financial interests, dual roles (e.g. PI and Doctor), as well as personal matters and career interests.
no

4.A. Study Type - (Boxes 4.1 to 4.2C) [View Form]

4.1. Application Type Indicate whether your application is Clinical or Behavioural.
Clinical

4.2.A. Institutions and Sites for Study (including study team members' institutional affiliations under which this research is being conducted)
Institution Site Providence Health Care St. Paul's Hospital

4.2.B. Non-UBC Institutions and Sites for Study (including study team members' institutional affiliations under which this research is being conducted)
Institution Site

4.2.C. Please enter any other locations where the research will be conducted under this Research Ethics Approval (e.g., Name of privately owned clinic, community centre, school, classroom, participant's home, in the field - provide details).
All aspects of this research will be undertaken at the BC Centre for Excellence in HIV/AIDS laboratories.

4.B. Clinical Study Type - (Boxes 4.2D to 4.5D) [View Form]

4.2.D. Roles of Study Sites and Institutions
Study Site:
Recruiting Team
Providence Health Care-St. no Paul's Hospital
Analysing AccessingData or
Records Utilizing or Charts: Lab
Space:
yes no yes
Participants: Member Affiliations:

4.3.A. If this proposal is closely linked to any other proposal previously/simultaneously submitted to a UBC REB or REBC institution, enter the Institution or Health Authority name and associated Research Ethics Board study number of that proposal. Institution Name:
PHCRI
REB study number:
H07-02559 - "HIV Drug and Immune Resistance in Canada: Mapping Transmission Throughout the provinces.

4.3.B. Please describe the relationship between this application and other ethics applications listed above.
Similarly to H07-02559, we propose to utilize Drug Treatment Program (DTP) data for phylogenetic analyses. However, this analysis proposes to additionally incorporate PrEP program data related to usage over time and prescription refills.

4.3.C. Have you received any information or are you aware of any rejection of this study by any Research Ethics Board? If yes, please provide known details and attach any available relevant documentation in Box 9.8.

no
Please provide known details: Not applicable

4.3.D. Will biological materials be collected or analyzed by researchers or a research lab?
no
If you are collecting and analyzing biological materials in your lab, please provide the UBC Biosafety Permit Number, or confirm that the lab has the appropriate biosafety permits in place.

4.3.E. Will radioisotopes be used in this project?
no
If YES, provide the institutionally applicable Radiation Permit Number(s).

4.4. Level of Risk After reviewing the minimal risk guidance notes and the criteria for minimal risk, does this study qualify for minimal risk review? Note that all studies which do not fall into the minimal risk category will undergo full board review.
yes

4.5.A. Peer review details:
This proposal has not received independent peer review. This study will only re-analyze de-identified previously obtained sequence data. There are no human participants. No new samples or data will be collected. Therefore this study meets the listed requirements for a minimal risk study.

4.6. Does this study require review and approval by another Canadian REB outside of Research Ethics British Columbia (REBC)? (Note that you CANNOT change your response to this question after the study has been approved, i.e. through an amendment.)
No

4.C. Clinical Study Type - (Boxes 4.7 to 4.8) [View Form]

4.7.A. Creation of a Registry (Data or Tissue Bank) Does this study involve the creation of a registry (data or tissue bank) with a local custodian for future use in other research? [if no, skip to 4.8]
No

4.7.B. Is the purpose of this application exclusively to obtain approval for the creation of a research database, registry or tissue bank? [Note if the creation of the database or registry or tissue repository is part of a bigger project also included in this application, you must answer no below.]
No

Clinical Chart Review
4.8.A. Is this an application for research requiring access to clinical charts OR data from registries or databases such as PopData BC or Pharmanet?
no

4.8.B. Insert the date range of the charts/data to be included in this research. (e.g. 7 September 2005 – 6 September 2011)

4.8.C. Is this study exclusively a retrospective chart review where the only source of data will be medical charts/records that are currently in existence? (i.e., will pre-date the date of your initial ethics approval?)

4.8.D. Will you have access to personally identifiable information?

4.8.E. Is this a retrospective chart review study for which participant consent will be obtained?

5. Summary of Study and Recruitment - Clinical Study [View Form]

5.1. Study Summary
5.1.A. Provide a short summary of the project written in lay language suitable for non-scientific REB members. DO NOT exceed 100 words and do not cut and paste directly from the study protocol.
Pre-exposure prophylaxis (PrEP) uptake has increased rapidly since its introduction. In order to better characterize the effectiveness of PrEP in reducing HIV transmission, we will compare changes in the basic reproduction number R0– the average number of secondary infections caused by an infectious person – over time in different clusters. Phylogenetic clusters are groups of similar viral sequences that are connected by recent transmission events. We expect to see differential reductions in cluster-specific R0 following the widespread availability of PrEP. We will further investigate whether PrEP users who seroconverted tended to join clusters and whether their viruses contained drug resistance mutations.

5.1.B. Summarize the research proposal: Purpose, Hypothesis, Justification, Objectives, Research Design and Statistical Analysis.

Purpose
To evaluate cluster-specific changes in reproduction number and other epidemiological parameters over time, specifically in response to the availability of PrEP. Further, to investigate factors associated with both clustering and drug resistance among PrEP users who seroconverted. These findings have direct implications for improving availability and consistent use of PrEP for individuals who are contacts of clusters that have a growing R0.

Hypotheses
- There are significant differences in cluster-specific estimates of R0, sampling proportion, and period of infectiousness over time, where at least one cluster maintained an R0 above 1 following the widespread availability of PrEP.
- PrEP users who seroconverted were more likely to harbor drug resistance mutations compared to non-PrEP users who seroconverted.
- PrEP users who seroconverted and joined a phylogenetic cluster (vs. did not join a cluster) were more likely to be young and living in Vancouver Coastal health authority.
- PrEP users who seroconverted and joined a phylogenetic cluster tended to join a cluster with a reproduction number greater than 1 in the preceding year.

Methodology
Dataset
We will connect HIV sequences with de-identified participants' sociodemographic and clinical information centralized in the DTP including sex, age, ethnicity, census tract of patient residence, physician census tract, date of seroconversion, date of first antiretroviral, treatment history, PrEP history, risk factors (men who have sex with men, MSM; people who inject drugs, PWID; heterosexual activity; blood exposure; previous HCV co-infection; other), plasma viral

RNA loads, CD4+ T cell count, having ever had acquired immune deficiency syndrome (AIDS); if applicable date of mortality and cause of death.

To maintain confidentiality and privacy, all patient data will be de-identified and all sequences will be assigned random six-character identifiers. The extraction, PCR, and sequencing of plasma HIV RNA was performed by clinical staff at the BCCfE and genotypic data were subsequently stored at the BCCfE access-controlled facility in a secure, encrypted, password protected Oracle database. Investigators (including co-investigators) will have no access to nominal data. Access to nominal data is restricted within the BC-CfE's DTP Clinical Registry exclusively to a select few dedicated DTP personnel (senior Oracle programmers and senior Data Analysts), who have access to identifiable data in the secure Oracle server or to the key that links to that data.

Phylogenetic Clustering

Sequences will be aligned and then a set of shuffled bootstrap alignments will be generated to infer 100 approximate maximum likelihood phylogenetic trees.

We will then identify phylogenetic clusters using the standard methodology applied for HIV public health cluster surveillance. By iterating this method of alignment, tree- building, and cluster identification over a longitudinally down- sampled dataset (to effectively blind ourselves to new data to mimic past inference back to 1996), we will model cluster growth over time, without accounting for sampling differences.

Phylodynamic inference of epidemiological parameters

We will stratify the dataset into cluster-specific HIV sequence alignments with the oldest sample from each patient. For all clusters, we will separately apply Bayesian birth-death skyline modelling using the BEAST2 software to estimate the reproductive number, sampling proportion, and period of infectiousness over time.

Hypothesis testing

We will investigate changes in R0 after PrEP between clusters using a non-parametric Kruskal-Wallis test. To test whether there was a reduction in R0 following PrEP for each cluster, we will build a generalized linear model, where each observation is a cluster and the outcome is R0. We will use Bayesian stochastic search variable selection to identify variables to include in the model and estimate their coefficient.

We are additionally interested in confirming whether the clusters with higher R0 were joined by seroconverted PrEP users. As there were only 12 known seroconverters who had used PrEP in BC (by early 2020), we would likely be underpowered to make a model with the outcome of whether or not seroconverted PrEP users joined a cluster. However, we will evaluate any trends in participant characteristics among clustering seroconverted PrEP users.

Finally, we will compare drug resistance patterns among PrEP users who seroconverted to all others who seroconverted in a multiple logistic regression framework, adjusting the p-value for multiple comparisons. It is possible that we will be underpowered to make any conclusions about the effect of PrEP. We will try to distinguish transmitted from acquired drug resistance on a case-by-case basis, taking into consideration the appearance of mutations in later samples

only, the estimated delay in diagnosis (initial CD4 count and viral load can be informative), whether they were using PrEP over that time period, and whether the phylogeny suggests that the virus' closest relative shared the drug resistance pattern.

5.2. Inclusion Criteria Describe the participants being selected for this study. List the criteria for their inclusion, and justify the grounds for their inclusion. If applicable, include age criteria for participants.

This is a retrospective study and will not involve any prospective recruitment of new subjects. All individuals registered in the Drug Treatment Program at the BCCfE between 1996 and the present with at least one HIV sequence will be included.

5.3. Exclusion Criteria Describe which potential participants will be excluded from participation. List the criteria for their exclusion, and justify the grounds for their exclusion.

Individuals in the Drug Treatment Program with no HIV sequences will be excluded.

5.4.A. Recruitment Provide a detailed description of the method of recruitment. Include, where applicable: a) who will contact prospective participants; b) by what means will recruitment be done (e.g., public posting, direct contact, third party recruitment, etc.); c) how will prospective participants be identified; d) all applicable site-specific information; e) attach letters of initial contact or other recruitment materials (i.e., posters, phone/email scripts) to page 9.
No subjects will be recruited for this study. This study uses retrospective data only.

5.4.B. Recruitment of Normal/Control Participants Describe how prospective normal/control participants will be identified, contacted, and recruited, if the method differs from the above.
Not applicable

5.5. Does this research focus on Indigenous peoples, communities, or organizations?
no

5.5.1.A. Will the research be conducted on Indigenous reserves, Métis settlement(s), or lands governed under a self- government agreement or an Inuit or First Nations land claims agreement?
If yes, please provide details:

5.5.1.B. Do any of the criteria for participation include membership in an Indigenous community, group of communities, or organization, including urban Indigenous populations?
If yes, please provide details:

5.5.1.C. Does the research seek input from participants regarding a community's cultural heritage, artifacts, traditional knowledge or unique characteristics?
If yes, please provide details:

5.5.1.D. Will Indigenous identity or membership in an Indigenous community be used as a variable for the purposes of analysis?
If yes, please provide details:

5.5.1.E. Will the results of the research refer to Indigenous communities, peoples, language, history or culture?
If yes, please provide details:

5.5.2. Community Engagement 5.5.2.A. If you answered yes to questions a), b), c), d), or e), have you initiated or do you intend to initiate an engagement process with the Indigenous collective, community or communities for this study?

5.5.2.B. If you answered Yes to question 5.5.2.A., describe the process that you have followed or will follow with respect to community engagement. Include the role or position of those consulted, including their names if appropriate. Attach any documentation of consultations (i.e. formal research agreement, letter of approval, email communications, etc.) below.
Attachment:

5.5.3. No community consultation or engagement If you answered no to question 5.5.2.A., briefly describe why community engagement will not be sought and how you can conduct a study that respects Indigenous communities and participants in the absence of community engagement.

5.6. Use of Records If existing records (e.g. health records, clinical lists or other records/databases) will be used to IDENTIFY potential participants for the purpose of recruitment, please describe how permission to access this information, and to collect and use this information, will be obtained.

Not applicable, there is not recruitment as part of study procedures.

5.7. Details of Study Procedures
Describe in a step-by-step manner the research procedures. When applicable, outline or describe standard of care or standard procedure. This is particularly important for addressing what is incremental to standard of care.

Dataset
We propose to re-analyze a de-identified dataset representing participants in the BC-CfE Drug Treatment Program (DTP) up until the Fall 2020, corresponding with the date of final ethics submission. We will analyze all available HIV sequences (there were 36271 partial pol sequences from 9630 PLHIV in February 2019) generated by the BC-CfE clinical HIV resistance genotype test. We will connect HIV sequences with de-identified participants' sociodemographic and clinical information centralized in the DTP including sex, age, ethnicity, census tract of patient residence, physician census tract, date of seroconversion, date of first

antiretroviral, treatment history, PrEP history, risk factors (men who have sex with men, MSM; people who inject drugs, PWID; heterosexual activity; blood exposure; previous HCV co-infection; other), plasma viral RNA loads, CD4+ T cell count, having ever had acquired immune deficiency syndrome (AIDS); if applicable date of mortality and cause of death.
To maintain confidentiality and privacy, all patient data will be de-identified and all sequences will be assigned random six-character identifiers. The extraction, PCR, and sequencing of plasma HIV RNA was performed by clinical staff at the BCCfE and genotypic data were subsequently stored at the BCCfE access-controlled facility in a secure, encrypted, password protected Oracle database. Investigators (including co-investigators) will have no access to nominal data. Access to nominal data is restricted within the BC-CfE's DTP Clinical Registry exclusively to a select few dedicated DTP personnel (senior Oracle programmers and senior Data Analysts), who have access to identifiable data in the secure Oracle server or to the key that links to that data.

Phylogenetic Clustering
Sequences will be aligned to the HXB2 reference genome (GenBank Accession #K03455) using MAFFT. Insertions and deletions relative to HXB2, as well as amino acids corresponding to WHO recognized drug resistance mutation sites, will be removed from the alignment for tree inference, however their presence will be noted as categorical variables. A set of shuffled bootstrap alignments will be generated to infer 100 approximate maximum likelihood phylogenetic trees, as implemented in FastTree2.1. Alternative tree-building software, ie IQ-TREE, will be compared using topological metrics and bootstrap support values.
We will then use the bootstrap trees to identify phylogenetic clusters using the standard methodology applied for HIV public health cluster surveillance. Specifically, we will compute pairwise patristic (tree) distance threshold of 0.02 substitutions per site for every bootstrap, identify pairs of nodes supported by >90% bootstraps, and then group nodes into clusters with 5+ members. We will evaluate different tree distance and bootstrap support thresholds, as well as a TN93 (HIV-TRACE) methodology.

By iterating this method of alignment, tree-building, and cluster identification over a longitudinally down-sampled dataset (to effectively blind ourselves to new data to mimic past inference back to 1996), we will model cluster growth over time, without accounting for sampling differences.

Phylodynamic inference of epidemiological parameters
We will stratify the dataset into cluster-specific HIV sequence alignments with the oldest sample from each patient. For all clusters, we will separately apply Bayesian birth-death skyline modelling using the BEAST2 software to estimate the reproductive number, sampling proportion, and period of infectiousness over time. If there is adequate evidence for population structure (by age group or geography) in a given cluster, then a multi-type birth-death (mtbb) model will be instead assumed. This model structure additionally parameterizes the viral migration rate between sub-populations. Other priors, such as the site model and molecular clock, will be justified by robust model testing and/or averaging.

Hypothesis testing
We will investigate changes in R0 after PrEP between clusters using a non-parametric Kruskal-Wallis test. To test whether there was a reduction in R0 following PrEP for each cluster, we will build a generalized linear model, where each observation is a cluster and the outcome is R0. We will use Bayesian stochastic search variable selection to identify variables to include in the model and estimate their coefficient. We will consider cluster-aggregated variables including but not limited to median age, % MSM, % PrEP use among new cases in cluster, aggregated diversification rate and viral load metrics, and % Vancouver Coastal. For comparison, the outcome of cluster growth rates could also be calculated using the number of confirmed cases in past year (normalized by cluster size).

We are additionally interested in confirming whether the clusters with higher R0 were joined by seroconverted PrEP users. As there were only 12 known seroconverters who had used PrEP in BC (by early 2020), we would likely be underpowered to make a model with the outcome of whether or not seroconverted PrEP users joined a cluster. However, we will evaluate any trends in participant characteristics among clustering seroconverted PrEP users.

Finally, we will compare drug resistance patterns (individual mutations and combinations) among PrEP users who seroconverted to all others who seroconverted in a multiple logistic regression framework, adjusting the p-value for multiple comparisons. It is possible that we will be underpowered to make any conclusions about the effect of PrEP. We will try to distinguish transmitted from acquired drug resistance on a case-by-case basis, taking into consideration the appearance of mutations in later samples only, the estimated delay in diagnosis (initial CD4 count and viral load can be informative), whether they were using PrEP over that time period, and whether the phylogeny suggests that the virus' closest relative shared the drug resistance pattern.

6. Participant Information and Consent Process - Clinical Study [View Form]

6.1. Time to Participate Not applicable

6.2. Time to Participate – Normal/Control Participants
Not applicable

6.3. Known Study Risks/Harms
There is little imaginable direct harm anticipated from this retrospective analysis of data. Data is de-identified and will be analyzed at the level of clusters of a minimum of 5, ensuring there is no plausible reason for the potential the loss of confidentiality when performing this research.

6.4. Potential Benefits Not applicable

6.5.A. Are there any costs participants can reasonably be expected to incur in order to participate – e.g. transportation, parking, child care, etc.? Specify what they are and whether or not these will be fully reimbursed.

Not applicable

6.5.B. Describe any remuneration (payments/incentives/gifts-in- kind) to be offered to the participants. Provide full details of the amounts, form of payment, payment schedules, and value of gifts-in-kind.
Not applicable

6.6. Obtaining Consent Please specify: a) who will explain the consent form, b) who will consent participants, c) details of where the consent will be obtained and under what circumstances, and d) the relationship between the person obtaining consent and the participant.
Not applicable. Participants will not be recruited for this study. This study will use previously stored, de-identified retrospective sequences and metadata in the DTP database.

6.7.A. Waiver/Alteration of Consent If you are asking for a waiver or an alteration of the requirement for participant informed consent, please justify the waiver or alteration and explain how the study meets all the criteria. CLICK on blue question mark. Ensure that you address each criteria individually. Include the corresponding letter (a, b, c, d, e) before each answer.

Consent will not be sought or obtained from patients for inclusion in this study, according to the waiver of consent criteria in TCPS2 Article 3.7:
a) The identifiable information is essential to the research as it permits modelling the structure of the study population based on age group and geography of residence, for instance. The probability of reidentification is low and the research involves minimal risk to participants.
b) The use of identifiable information without the participants' consent is unlikely to adversely affect the welfare of participants.
c) We will comply with any known preferences previously expressed by participants to not have their information used for research purposes.
d) It is not feasible to obtain consent from all the participants in the Drug Treatment Program, as they number well over 10 000. Further, some participants have passed away, are challenging to contact due to insecure housing or disconnection with care, or have chronic illness. Excluding these participants would result in a sampling bias in the study group.
e) N/A

6.7.B. Waiver of Consent in Individual Medical Emergencies If you are asking for a waiver or an alteration of the requirement for participant informed consent in individual medical emergencies, please justify the waiver or alteration and explain how the study meets all the criteria. CLICK on blue question mark. Ensure that you address each criteria individually. Include the corresponding letter (a, b, c, d, e, f) before each answer.
Not applicable

6.8. Time to Consent How long after being provided with detailed information/consent form about the study will the participant have to decide whether or not to

participate? Provide your rationale for the amount of time given.
Not applicable

6.9. Capacity to Consent Will participants have the capacity to give fully informed consent on their own behalf?
Not Applicable

6.9.A. Provide details of the nature of the incapacity (for instance, young age, mental or physical condition).

6.9.B. If a participant does not have the capacity to give fully informed consent, who will consent on their behalf? Ensure the relevant consent form (parent/caregiver, substitute decision maker, legally authorized representative) is attached to page 9.

6.9.C. If a participant does not have the capacity to give fully informed consent, will they be able to give assent to participate?

6.9.D. If yes, explain how assent will be sought. Please be sure to attach copies of the assent form to page 9.

6.10. Describe how participants' ongoing consent will be maintained throughout the research
Not applicable

6.11. Provisions for Consent (e.g., special assistance, Braille, translations/translator)
Not applicable

6.12. Restrictions on Disclosure Describe any restrictions regarding the disclosure of information to research participants (during or at the end of the study) that the sponsor has placed on investigators, including those related to the publication of results.
Not applicable

6.13. Communication of Study Results Indicate plans for communicating study results to participants.
Not applicable

7. Number of Participants and Study Drugs - Clinical Study [View Form]

7.1. Other Study Sites
7.1.A. Is this research being conducted at any sites other than those selected on page 4 of this RISe submission, including world- wide?
no
If known, please list the other sites below:
Not applicable

7.1.B. Is this study being submitted for ethical approval to any other Research Ethics Board not covered by this RISe submission, including worldwide?

Description:
No

If yes, please provide the name of the REB(s) and if available, contact information:
Not applicable

7.2. Number of Participants
7.2.A. How many participants (including controls) will be enrolled in the entire study (world-wide)?
9630

7.2.B. How many participants (including controls) will be enrolled at institutions covered by this Research Ethics Approval?
9630

7.2.B.2. If possible, breakdown the estimated number per institution.
Not applicable

7.2.C. Of these, how many are controls?
0
If possible, breakdown the estimated number per institution.
Not applicable

7.2.D. Please enter any additional comments. If your study does not involve enrollment of human participants, please enter the number of records or samples to be obtained:
No additional comments.

7.3. Drug approvals Enter the generic name of any investigational drug(s) not yet approved or any marketed drug(s) used outside of its approved indication.
Not applicable

7.4. Marketed Drugs Enter the name of any marketed drug(s) used within its approved indication.
Not applicable

7.5. Natural and Non- Prescription Health Products
Not applicable

7.6. Experimental Devices Enter the name of any new investigational devices, or marketed devices used in experimental mode, that will be used outside of their approved indication.
Not applicable

7.7. PERs If applicable, enter the name of any positron- emitting radiopharmaceuticals (PERs).
Not applicable

7.8. Health Canada Regulatory Approvals 7.8.A. Is this study a clinical trial of a drug, device, or natural health product requiring Health Canada regulatory approval (If this study does not require Health Canada approval, skip to 7.10)

7.8.B. If yes, check all that apply from the list below.
Description Regulatory Approval:

7.8.C. Name the sponsor/institution/investigator responsible for filing a Clinical Trial Application (CTA) or Investigational Testing Authorization (ITA) with Health Canada or Other.
Not applicable

7.9. Details of Health Canada Regulatory Approvals A copy of the Health Canada approval must also be attached in Box 9.1. 7.9.A. Name of Regulatory Agency
Name of Date of Date of Pending Agency Approval Application:

7.9.B. Health Canada NOL Control Number
Health Canada NOL Control Number Date of Approval

7.10. Stem Cell Research Does this research fall within the categories of pluripotent stem cell research that need to be submitted to the CIHR Stem Cell Oversight Committee (SCOC)?
no

7.11. Registration for Publication of Clinical Trials

7.11.A. Does this clinical study fall within the definition stated in the guidance (Click blue question mark)?
no

7.11.B. If yes, click Add to enter the following information.
Has it been Indicate the Enter your Clinical
registered? Authorized Registry Trial unique identifier: used:

7.12. US Regulatory Requirements 7.12.A. Is there a requirement for this research to comply with United States regulations for research ethics?
no

7.12.B. If yes, A) please indicate whether or not an FDA Investigational New Drug (IND) number (drug studies) or an FDA Investigational Device Exception (IDE) is required for the research. B) Enter the applicable number below and C) provide documentation from the Sponsor or the FDA verifying the IND/IDE number, or explaining the study exemption status, in Box 9.1.C.

Not applicable

8. Data Monitoring and Storage - Clinical Study [View Form]

8.1. Unblinding in an Emergency Describe the provisions made to break the code of a double-blind study in an emergency situation, and indicate who has the code.
Since this is a retrospective anonymous analysis there is no foreseeable need for emergency unblinding in this study. There would be no added direct benefit to patients.

8.2. Data Monitoring Procedures Describe data monitoring procedures while research is ongoing. Include details of planned interim analyses, Data and Safety Monitoring Board, or other monitoring systems.
Quality control measures such as phylogenetic checks for contamination and sample confusion will be applied to all sequences. All genotype data was generated by clinical staff at the BCCfE following clinical laboratory standard quality control measures. The data will be stored in a secure, encrypted, password protected Oracle database. There is not Data and Safety Monitoring Board as part of this research.

8.3. Study Stoppage Describe the circumstances under which the ENTIRE study could be stopped early. Should this occur, describe what provisions would be put in place to ensure that the participants are fully informed of the reasons for stopping the study.
Not applicable, as this is a retrospective study, we cannot think of likely scenarios where the study would be stopped early

8.4. Personal Identifiers
8.4.A. Describe how the identity of the participants will be protected both during and after the research study, including how the participants will be identified on data collection forms, biospecimen labels, photos, videos, scans etc.
Retrospective sample data being used will be de-identified. There is no reason to go back to the patient, nor would it be possible to do so.

8.4.B. Will any personal health information or personal identifiers be retained as part of the dataset?
no
If yes, please describe what personal identifying information will be collected, and justify the need for it to be collected.
While full date of death is available for those applicable, we will specifically request only month and year of death to remove any potential identifiers.

8.5. Data Access and Storage
8.5.A. a) Explain who will have access to the data at each stage of processing and analysis; b) indicate whether a current list of the names of study personnel (including co- investigators and research staff) and their delegated tasks will be maintained in the study file; c) if a list will not be maintained, please explain.

Access will be limited to those working on the study including the principal investigator, Dr. Jeffrey Joy and Angela McLaughlin.

8.5.B. Describe how the data will be stored (e.g., computerized files, hard copy, video-recording, audio recording, personal electronic device, other). Please confirm that any digital data will be stored on an encrypted, password protected computer, storage device, or hospital network server.

Data will be stored in restricted access/locked rooms. All data will be de-identified and stored on encrypted computers in password protected computerized files at the BCCfE.

8.5.C. Describe the safeguards in place to protect the confidentiality and security of the data.
Data will be stored in restricted access/locked rooms. All data will be de-identified and stored on encrypted computers in password protected computerized files at the BCCfE.

8.5.D. If any data or images are to be kept on the Web, what precautions have you taken to prevent it from being copied?
Not applicable

8.6. Disposition of Study Data and Biospecimens

8.6.A. Please describe: a) what will happen to the data at the end of the study; b) how long the study data will be retained; c) when and how the data will be destroyed; d) what plans there are for future use of the data; and e) who will have access to the data in the future and for what purpose.
Results of this study will be be published as a part of a peer reviewed research article and/or conference proceedings. Study data/findings will be kept for a minimum of 5 years after publication. Data will ultimately be overwritten 35 times using a Guttman overpass algorithm to securely delete from the computer systems.

8.6.B. If applicable, for each study component (eg, Main study, and Sub-studies): a) describe what will happen to the study biospecimens at the end of the study; b) how long the study biospecimens will be retained; c) where, when and how the biospecimens will be destroyed d) what plans there are for future use of the biospecimens, including who will have access to the biospecimens in the future and for what purpose.
Not applicable

8.7. Data and/or Biospecimen Transfer Out of BC Site(s) 8.7.A. Will data and/or biospecimens be sent outside of the BC site(s) where it is being collected?
no

8.7.B. If yes, please describe: a) the type of data and/or biospecimens to be transferred;

b) who the data and/or biospecimens will be transferred to; c) where the data and/or biospecimens will be transferred (list institution & location); and d) how the data and/or biospecimens will be sent.
Not applicable

8.8. Data and/or Biospecimen Transfer Received by BC Site(s) 8.8.A. Will the BC researchers be receiving data and/or biospecimens from other sites?
no

8.8.B. If yes, please describe: a) the type of data and/or biospecimens to be received; b) who the data and/or biospecimens will be received from; c) where the data and/or biospecimens will be received from (list institution and location); and d) how the data and/or biospecimens will be received.
Not applicable

8.9. Data Linkage 8.9.A. Will the data be linked to any other data source (including a biorepository)?
no

8.9.B. If yes: a) Identify the data set; b) how the linkage will occur; and c) explain how confidentiality regarding the shared information will be preserved.
Not applicable

9. Documentation - Clinical Study [View Form]

9.1.A. Protocol
DocumentName
Password VersionDate (if applicable)

Cluster Dynamics - July Protocol Version dated 24 24, July 2020 2020

9.1.B. Health Canada regulatory approval (receipt will be acknowledged). Please include details of this approval in Box 7.9 of the RISe application form.
Document Name Version Date Password (if applicable)

9.1.C. FDA IND or IDE letters (receipt will be acknowledged)
Document Name Version Date Password (if applicable)

9.2. Consent Forms
Document Name Version Date Password (if applicable)

9.3. Assent Forms
Document Name Version Date Password (if applicable)

9.4. Investigator Brochures/Product Monographs
Document Name Version Date Password (if applicable)

9.5. Advertisement to Recruit Participants
Document Name Version Date Password (if applicable)

9.6. Questionnaire, Questionnaire Cover Letter, Tests, Interview Scripts, etc. Please attach each separately.
Document Name Version Date Password (if applicable)

9.7. Letter of Initial Contact
Document Name Version Date Password (if applicable)

9.8. Data collection forms and Other Documents

9.8.A. Please attach data collection forms, chart extraction forms, case report forms, or other documents.
Document Name
DataCollectionSheet_DTP2020.xlx
Password Version Date (if applicable)

June 13, 2020

9.8.B. If a website is part of this study, enter the URL
below. Since URLs may change over time or become non-existent, you must also attach a copy of the documentation contained on the web site to this section or provide an explanation.

10. Fee for Service - Providence Health Care [View Form]

10.1. A. Select one of the following:
Fee N/A as per above criteria

10.1. B. Additional Information: No additional information

11. Hospital Information - Providence Health Care [View Form]

11.1.
11.1.A. Which of the following hospital services are required for the conduct of your research? (Please check all that apply).
Centre for Excellence in HIV/AIDS

11.1.B. If Other provide details below.

11.2.

11.2.A. Which of the following hospital areas will be required to provide services for the conduct of the research? If the PI for the research is employed by the hospital area in question and has obtained approval for use of his or her own area, please do not select the relevant option. (Please check all that apply).
Centre for Excellence in HIV/AIDS

11.2.B. Provide details below of other hospital areas affected by the study.

11.3. Does the Principal Investigator in Box 1.1 have a UBC appointment?
yes
Declaration Form if PI doesn't have UBC appointment

12. Save Application - Human Ethics [View Form]
Print
Close
