# Supplementary material for: Heterogeneous impacts of HIV pre-exposure prophylaxis (PrEP) on drug resistance and phylogenetic cluster transmission dynamics in British Columbia, Canada: A retrospective cohort and simulation study
Source: PLoS Med. 2025 Dec 9;22(12):e1004827. doi: 10.1371/journal.pmed.1004827 (PMC12700434; doi:10.1371/journal.pmed.1004827)
Supplement: Attachment — Submitted filename: Final.PLOSMed_Main_HIV-PrEP-Dynamics_0502_edits.docx [file pmed.1004827.s005.docx]

Impact of pre-exposure prophylaxis on HIV drug resistance and phylogenetic cluster growth

Angela McLaughlin^1,2^, Junine Toy^1^, Vince Montoya^1^, Paul Sereda^1^, Jason Trigg^1^, Mark Hull^1^, Chanson J. Brumme^1,3^, Rolando Barrios^1^, Julio S.G. Montaner^1,3^, Jeffrey B. Joy^1,2,3*^

^1^ British Columbia Centre for Excellence in HIV/AIDS, Vancouver, Canada

^2^ Bioinformatics, University of British Columbia, Vancouver, Canada

^3^ Department of Medicine, University of British Columbia, Vancouver, Canada

* Corresponding author

Email: jjoy@bccfe.ca

**Abstract**

**Background:** HIV pre-exposure prophylaxis (PrEP) prevents infection when taken during periods of risk, however, its population-level effectiveness is hindered by incomplete uptake, adherence, and retention. Since oral PrEP became available free-of-cost in British Columbia (BC), Canada, in January 2018, uptake has been rapid among gay, bisexual, and other men who have sex with men (GBM). Mathematical models suggested PrEP reduced new infections synergistically with free antiretroviral therapy in BC. We sought to evaluate individual and population-level impacts of PrEP on HIV drug resistance and transmission dynamics in phylogenetic clusters to elucidate the heterogeneity of its effectiveness.

**Methods and Findings:** We applied a retrospective cohort design to evaluate whether baseline drug resistance mutations and phylogenetic clustering were more likely among newly diagnosed PrEP users in BC (n=39) compared to non-PrEP users (n=566) newly diagnosed over the same period, 2018-2022. Newly HIV diagnosed PrEP users were significantly more likely than non-PrEP users to join clusters and carry baseline nucleoside-analogue reverse transcriptase inhibitor (NRTI) resistance mutation M184I/V. Subsequently, population-level impact of widespread PrEP availability on reducing transmission, quantified by the effective reproduction number (R_e_), was assessed for key populations living with HIV in BC and across active phylogenetic clusters. Empirically tuned simulations of active clusters’ growth based on observed R_e_ with or without estimated PrEP impacts were used to estimate diagnoses averted via PrEP across clusters. Despite R_e_ reductions in the GBM population overall and in 50% of active GBM-predominant clusters following PrEP, we highlight clusters of predominantly GBM as well as clusters of people who inject drugs with high and increasing R_e_.

**Conclusions:** We estimated that PrEP has averted approximately 20 new HIV diagnoses per year in BC since 2018, while infrequently contributing to baseline drug resistance. These findings corroborate PrEP effectiveness and pinpoint groups for prioritized PrEP services.

**Introduction**

Optimal control of HIV/AIDS requires equitable delivery of antiretroviral treatment (ART) to all people living with HIV (PLWH) immediately following HIV diagnosis, and prevention interventions, including pre-exposure prophylaxis (PrEP), to populations at elevated risk, informed by longitudinal epidemiological monitoring, including via phylogenetic identification of HIV transmission clusters. Although HIV morbidity, mortality and incidence have declined in many regions, including in British Columbia (BC), Canada (1), ongoing transmission continues to disproportionately affect certain populations.

Oral PrEP effectively prevents HIV-1 acquisition when taken during periods of risk among gay, bisexual, and other men who have sex with men (GBM) (2), transgender women (TGW) (2), heterosexuals (3), and people who inject drugs (PWID)(4). Barriers to access, racial disparities (5), limited eligibility or coverage, inconsistent adherence (6), and program non-retention (7) have resulted in sub-optimal oral PrEP effectiveness. In 2016, Health Canada approved once-daily oral PrEP, Truvada^®^, comprised of tenofovir disoproxil fumarate (TDF)/emtricitabine (FTC) for adults at elevated risk of HIV acquisition, including GBM and serodiscordant heterosexual couples (8), followed by generic TDF/FTC in 2017 (9).

Since January 1, 2018, PrEP has been fully publicly funded and centrally distributed for BC residents with greater likelihood of HIV acquisition as determined by local PrEP guidelines (10). PrEP uptake in BC has expanded rapidly, with 9737 cumulative participants by June 30, 2022 (7,11), consisting of 97.0% cis GBM, 1.3% TGW, 0.9% cis-women, and 0.5% transgender men (TGM) (7). Non-cisgender men (TGM, TGW, cis-women) were less likely to persist with PrEP, as were those who were younger and had no prior PrEP use (7). Differences in PrEP awareness, uptake, access, and persistence manifest as differential reductions in HIV transmission. Lapsed prescriptions (greater than six months beyond expected refill date based on daily PrEP use) and incomplete adherence have resulted in 39 newly diagnosed HIV infections in PrEP users between October 23, 2018 and December 5, 2022 (0.4% of PrEP participants).

Population-level effectiveness of PrEP has been estimated through cohort studies, simulations, and mathematical models, but not often at the phylogenetic cluster level. In a compartmental model of HIV in BC, prioritized provision of PrEP to GBM with higher likelihood of HIV acquisition, in combination with Treatment as Prevention (TasP), was associated with reduced incidence and R_e_ below one in susceptible GBM (12,13). Clustering the BC population by reported potential HIV exposure, testing behaviour, and PrEP interest increased the estimated population-level effectiveness of PrEP (14). In the Australian EPIC-NSW cohort study, PrEP availability resulted in a 25% relative reduction of new HIV diagnoses in GBM (15). In South Africa, prioritizing PrEP to groups with higher likelihood of HIV acquisition was more efficient than based on partnership alone (16). A stochastic mechanistic model suggested PrEP averted 20% of HIV acquisitions from 2015-2021 in Montreal (17). Expanding oral PrEP coverage to 80% was estimated to avert 8% of new HIV infections in Australia, 15% in Thailand, and 26% in China over 40 years (18). In the US, network-based mathematical models were applied to estimate that 33% of cases over the next decade could be averted with 40% coverage of MSM and 62% adherence (19). No HIV seroconversions were reported over 850 person-years of PrEP use in Northern California, however there were two seroconversions in individuals who had discontinued PrEP use (20), highlighting the importance of program retention and support.

Pre-treatment drug resistance, either through the transmission of resistant variants, or acquired resistance due to the use of TDF/FTC during undiagnosed or acute HIV infection, adversely impacts clinical outcomes by limiting treatment options (21–23). Mutations conferring resistance to NRTI drugs in PrEP, TDF (K65R) and FTC (M184V/I), were identified in individuals retrospectively found to have acute HIV infections who were prescribed PrEP (24). In New York, past and recent PrEP use were associated with significantly elevated risk of baseline M184I/V, adjusted for transmission risk group (21). In a meta-analysis, individuals exposed to PrEP during acute infection were significantly more likely to have TDF or FTC resistance (25). HIV acquisition during PrEP use has occurred in randomized control trials, but is confounded by incomplete adherence and reporting bias (24). Evaluating HIV drug resistance in phylogenetic trees can illuminate transmission of drug resistance in expanding phylogenetic clusters, routinely monitored for HIV surveillance in BC (26), which can represent groups linked by recent transmission.

In this study, we sought to estimate impacts of PrEP use on baseline drug resistance and phylogenetic clustering among new HIV diagnoses, and the population-level impacts of PrEP on the effective reproductive number (R_e_) of phylogenetic clusters. We tested the hypotheses that i) newly diagnosed PrEP users were more likely to cluster than non-PrEP users, under the expectation that PrEP eligibility and use is related to behaviour associated with potential exposure and linkage to sexual transmission networks, and that ii) they have elevated baseline NRTI resistance, under the expectation that incomplete PrEP adherence led to suboptimal drug concentration during acute infection. We further hypothesized that iii) cluster-specific R_e_, and diagnoses averted have been heterogeneous since PrEP availability, amid broad R_e_ reductions in the BC GBM population.

**Methods**

**Study setting and data source**

HIV sequences and patient metadata for this study were obtained on February 9, 2023, representing data up to December 31, 2022, from the Drug Treatment Program (DTP) at the BC Centre for Excellence in HIV/AIDS (BC-CfE) representing all PLWH connected to publicly-funded treatment in BC, Canada. This study includes 14,919 DTP participants, of which 10,740 had at least one HIV partial pol sequence available, De-identified participant metadata included gender ([cis-]male; trans-male; [cis-]female; trans-female), sex-at-birth, age, residence census tract and regional health authority, prescriber census tract, date of first viral load (VL) test, date of first ART, ART prescriptions, PrEP dispensations (if applicable), self-reported risk exposures at enrolment i.e., key populations (GBM; PWID; heterosexual (HET); blood; hepatitis C virus; other), VL over time, baseline CD4 cell count, prior acquired immune deficiency syndrome (AIDS) diagnosis, and if applicable, date of death.

Sequencing was performed by clinical staff at the BC-CfE and genotypic data were stored in the BC-CfE access-controlled facility within a secure, encrypted Oracle database. Investigators had no access to nominal data. Research ethics were approved by University of British Columbia-Providence Health Care Research Institute (REB #H20-02859) on December 7, 2020 and amended September 8, 2022**.**

**Phylogenetic and drug resistance analyses**

We analyzed 41,941 HIV-1 partial pol (*protease* and partial *reverse transcriptase*) sequences from 10,740 DTP participants collected between May 30, 1996 and December 31, 2022 and 8739 *integrase* (int) sequences from 4052 patients (1-35 sequences per patient). Additional details on cleaning data of incomplete dates and duplicate identifiers in Supplementary. Partial pol and int sequences were aligned to the HXB2 reference genome (GenBank Accession #K03455) using minimap2 parallelized by viralMSA (1,2). COMET was used to assign HIV-1 subtypes using partial pol (3). For COMET subtype assignments with less than 90% bootstrap support, subtype assignment was evaluated using REGA (4), and the assignment with higher support was chosen. Sequences from newly diagnosed individuals were screened for resistance-associated mutations (RAMs) using the Stanford HIV Drug Resistance Database (HIVdb) version 9.6 (27). Codons of surveillance drug resistance mutations (SDRMs) (5) and insertions relative to reference HXB2 were removed prior to phylogenetic inference.

A set of 100 bootstrap alignments were generated to infer bootstrap approximate maximum likelihood (ML) phylogenetic trees in FastTree 2.1 with a generalized time reversible (GTR) substitution model (28). Trees were outgroup rooted on the oldest subtype B sequence. Phylogenetic clusters were identified with a minimum of five members with viruses sharing pairwise patristic distance <0.02 substitutions per site in greater than 90% of bootstraps (26). We evaluated the effect of rooting using outgroups, midpoint, and root-to-tip regression on the distribution of cluster sizes (Fig. S3). Separate trees were inferred for key clusters with IQ-TREE 2 under a GTR substitution model with 1000 resamples for ultrafast bootstrap support values (29,30), then rooted using root-to-tip regression using sample collection dates optimized by residual mean squares in ape (Fig. S7-15)(31).

**Statistical comparison of newly diagnosed with and without previous PrEP use**

To evaluate individual-level impacts of PrEP on baseline drug resistance and clustering, we applied a retrospective cohort study design to compare 39 newly diagnosed PrEP users with date of first detectable VL from October 23, 2018 to December 5, 2022, and a control group of 566 newly diagnosed non-PrEP users over the same period. Individuals enrolled for PrEP who never filled prescriptions were not considered PrEP users. New participants with previous ART experience (PLWH who have immigrated) were excluded.

Characteristics associated with PrEP use among newly diagnosed were evaluated with chi-square tests for categorical variables and Kruskal-Wallis tests of medians for numeric variables (Table S3), as well as characteristics associated with clustering stratified by PrEP use (Table S4). We tested the hypotheses that newly diagnosed PrEP users included a higher proportion of clustered individuals and higher proportion of baseline NRTI resistance than non-PrEP users. Chi-squared tests were applied if greater than 80% of expected frequencies exceeded five, otherwise Fisher’s exact tests were applied. To corroborate differences in clustering, lineage-level viral diversification rates in newly diagnosed with or without PrEP were compared using Kruskal-Wallis tests (Supplementary). Proportions of newly diagnosed with and without PrEP use who had baseline drug resistance mutations were compared using Fisher’s exact tests with p-values adjusted using the Benjamini-Hochberg method (Table 2) (37).

**Effective reproduction number dynamics since PrEP**

Diagnoses over time were used to estimate incidence (33) and the instantaneous effective reproduction number (R_e_) in BC, for key populations in the DTP, and for active phylogenetic clusters (with at least one new diagnosis since 2018 and at least 10 members) using EpiEstim with gamma-distributed serial intervals (34). Parameters compared for R_e_ estimation in BC (Figs. S19-22), key populations (Figs. S23-24), and clusters (Figs. S25-28) included serial interval mean of 0.5 year (y), 1 y, 2 y, or 5 y, and standard deviation (sd) of 0.5 y, 1 y, or 2 y; estimation interval of 0.25 y, 0.5 y, or 1 y; and R_e_ smoothing over 30 days (d), 90 d, or 365 d. Primary analyses represent a serial interval with mean 1 y, sd 0.5 y, estimation interval 0.5 y, and 90 d R_e_ smoothing. We fitted average R_e_ in the periods before PrEP (Jan. 2016 – Dec. 2017), during PrEP and before COVID-19 (Jan. 2018 - Feb. 2020), during PrEP and COVID-19 (Mar. 2020 – Feb. 2022), and during PrEP and post-COVID-19 (Mar. 2022 - Dec. 2022). Fold-change (FC) in R_e_ with PrEP (‘PrEP effect’) was calculated as the ratio of R_e_ during PrEP and before COVID-19 versus R_e_ before PrEP in key populations and clusters.

**HIV cases averted by PrEP in phylogenetic cluster growth simulations**

Stochastic branching processes were used to simulate growth of active HIV clusters in BC with and without PrEP availability, whereby the number of secondary cases in each generation is drawn from a negative binomial distribution defined by mean of R_e_ and dispersion, k (38–40), reflecting infrequent superspreading events (15). R_e_ is either the observed cluster R_e_ (to recapitulate reality with PrEP) or counterfactual cluster R_e_ adjusted by the PrEP effect, which is the cluster-level FC in R_e_ with PrEP (Fig. 4C, Fig. S20, Fig. S21). Observed cluster R_e_ with 95% confidence width greater than 10 (sparse data) or NA were replaced with R_e_=0.8 to reflect inactivity. If PrEP effect could not be calculated for a cluster, either because it was a new cluster seeded since 2018 or Re was NA before or after PrEP, then the PrEP effect of the clusters’ predominant population was assigned. For each generation, R_e_ was permitted to deviate from the specified value by drawing from a binomial distribution with 50% probability of success, where R_e_ was then drawn from a gamma-distribution centered on cluster R_e_ (sd=0.1). We assumed a gamma-distributed serial interval (mean 1 y, sd 0.5 y) and k sampled uniformly between 0.1 and 0.3 for each generation.

Initially, the number of infectious individuals (starting seed) at time zero (January 1, 2018) for each active cluster was estimated as cases minus deaths and emigrations at the end of 2017, multiplied by the proportion of diagnosed cases on ART and virally unsuppressed, based on estimates from 2019 that 82% of cases in BC were diagnosed, 76% of those diagnosed were on ART, and 83% of those on ART were virally suppressed (16). Seeds were calibrated to align observed new cases in each cluster from 2018 to end of 2022, with the mean number of sampled new cases from simulations using observed cluster R_e_ (Table S6, Fig. S22). The adjusted seed size was applied in all subsequent simulations based on observed R_e_ (with PrEP available) and counterfactual R_e_ (in the absence of PrEP). Seeds were enforced to be a minimum of one, including for clusters seeded since 2018.

For scenarios with or without PrEP, 4000 simulations were run per cluster and for non-clustered cases (grouped as cluster 9999) ). Simulations began on day zero, January 1, 2018 and ran to January 1, 2023 (1827 d). Infected cases resulting from simulations were stochastically diagnosed with probability sampled from a log-normal distribution centered on 0.82, and diagnosed cases were sampled (i.e., sequenced) with probability drawn from a log-normal distribution centered on 0.95 (informed by ratio of DTP participants since 2018 with a pol sequence. Confidence intervals of diagnoses and samples averted were calculated by sampling 95% of simulations without removal 1000 times, then calculating normally-distributed confidence intervals across bootstrap means. Cluster diagnoses averted were calculated as the difference in mean and 95% CI of cumulative diagnoses in simulations with and without PrEP (Fig. S23, Fig. S24). Poisson generalized linear models were evaluated to identify cluster characteristics associated with the most and fewest diagnoses averted (Table S7). Diagnoses or samples averted were shifted up by the minimum averted to remove negative integers; factors evaluated included cluster size in 2017, predominant and percentage key populations, percentage health authority of residence, median member age in 2023 and age at first ART, and percentage of new diagnoses with PrEP experience. The sum of all cases averted across clusters was applied to estimate the projected lifetime ART cost savings, by multiplying cases averted by the average lifetime cost of treating a PLWH in Canada ($250,000) (35). This does not constitute a cost-effectiveness analysis, as we do not compare the costs of PrEP (36).

**Data availability**
The BC-CfE is prohibited from making individuals’ viral sequences or associated metadata publicly available due to provisions in our service contracts, institutional policy, and ethical requirements. Code is available upon request.

**Results**

**Clustering of newly HIV diagnosed PrEP users and non-PrEP users**

Newly HIV diagnosed PrEP users (n=39) in BC were significantly more likely than newly diagnosed non-PrEP users (n=566) to be male (100% vs. 79.5%; chi-squared test, p=0.011; Table S3), GBM (97.1% vs 56.7% of reported, p<0.001), reflecting that the majority of PrEP users are GBM, younger at first ART (median 32 vs. 37 years old; Kruskal-Wallis test, p=0.033), and have a higher baseline CD4+ T-cell count (490 vs. 380, p=0.003).

By December 31^st^, 2022, there were 246 BC HIV phylogenetic clusters (comprising at least five individuals with viruses sharing pairwise patristic distance less than 0.02 substitutions per site in at least 90% of bootstraps), 84 of which were active, with at least one new diagnosis since 2018. Tree method negligibly affected cluster identification (Fig. S3). All clusters joined by PrEP users were also joined by at least one non-PrEP user (Fig. S1). Newly diagnosed PrEP users were significantly more likely to cluster than non-PrEP users (chi-squared test p=0.0075, Fig. S2). Of 39 newly diagnosed PrEP users, 30 joined 13 unique clusters (76.9% clustered, one-group proportions test 95% confidence interval (CI): 60.3-88.3%), compared to 303 of 566 non-PrEP users (53.5% clustered, 95% CI: 49.3-57.7%), who joined 84 unique clusters (Table 1). Among GBM, 82.4% of newly diagnosed PrEP users clustered, compared to 54.7% of non-PrEP users (Table S4). Newly diagnosed PrEP users also had significantly higher lineage-level viral diversification rates, indicative of rapid, recently sampled transmission events, than non-PrEP users (Fig. S2). In a logistic regression model, newly diagnosed PrEP users had 2.6-times (95% CI: 1.2-6.4) higher odds of clustering compared to non-PrEP users, adjusted for GBM, age at first ART and baseline CD4 count.

**Table 1. Total and percentage in phylogenetic clusters among newly HIV diagnosed in BC from 2015-2022, stratified by PrEP use since 2018.**

|  | **All newly diagnosed** | | | | **Newly diagnosed, no PrEP** | | | | **Newly diagnosed, PrEP** | | | |
| --- | --- | --- | --- | --- | --- | --- | --- | --- | --- | --- | --- | --- |
| **Year** | **N** | **N clustered** | **% clustered** | **n clusters** | **N** | **N clustered** | **% clustered** | **n clusters** | **N** | **N clustered** | **% clustered** | **n clusters** |
| 2015 | 256 | 140 | 54.7 | 48 |  |  |  |  |  |  |  |  |
| 2016 | 252 | 121 | 48 | 45 |  |  |  |  |  |  |  |  |
| 2017 | 183 | 103 | 56.3 | 43 |  |  |  |  |  |  |  |  |
| 2018 | 174 | 94 | 54 | 40 | 173 | 93 | 53.8 | 40 | 1 | 1 | 100 | 1 |
| 2019 | 168 | 83 | 49.4 | 43 | 160 | 78 | 48.8 | 42 | 8 | 5 | 62.5 | 4 |
| 2020 | 127 | 83 | 65.4 | 30 | 121 | 78 | 64.5 | 30 | 6 | 5 | 83.3 | 5 |
| 2021 | 131 | 74 | 56.5 | 34 | 125 | 70 | 56 | 34 | 6 | 4 | 66.7 | 3 |
| 2022 | 124 | 67 | 54 | 24 | 106 | 52 | 49.1 | 24 | 18 | 15 | 83.3 | 7 |
| **Sum N or Mean %, 2018-2022** | | | | | 685 | 371 | 54.2 | 84 | 39 | 30 | 76.9 | 13 |

**PrEP use associated with elevated probability of baseline NRTI drug resistance**

Drug resistance mutations were identified and resistance scores were calculated using Stanford HIVdb version 9.6 (41). Differences in prevalence of baseline resistance-associated mutations (RAMs) between newly diagnosed PrEP users and non-PrEP users were evaluated by drug class (Fig. S5) and mutation (Fig. 1, Table 2). By drug class, there were no significant differences in the proportion of newly diagnosed PrEP users and non-PrEP users with baseline RAMs to any drug class (chi-squared test, p=0.12), or to specific drug classes (NNRTI, Fisher’s exact test: p=0.12; NRTI, p=0.26; PI, p=0.2 (Fig. S5). Among 39 newly diagnosed PrEP users, eight individuals had baseline NRTI RAMs.	Baseline NRTI resistance mutation M184V and M184I/V were more frequent in newly diagnosed PrEP users than non-PrEP users (Fig. 1), conferring elevated resistance scores to FTC, lamivudine (3TC), and abacavir (ABC) (Fig.S6). Three of 39 newly diagnosed PrEP users (7.7%) had baseline M184V or M184I/V, compared to 2 of 566 (0.35%) non-PrEP users (Fisher’s exact test, adjusted p-value=0.025; Table 2; power=79% at alpha=0.05). M184I/V was the only baseline RAM significantly more common in PrEP users than non-PrEP users (Table 2). Newly diagnosed PrEP users with baseline M184V had the two lowest proportion of days covered by PrEP (0.60 and 0.63 vs. median=0.88 across newly diagnosed PrEP users) and had significantly shorter time from last PrEP dispensation to first detectable VL (76 d vs. 373 d; Kruskal-Wallis test, p=0.045). The newly diagnosed PrEP user with M184I/V had a PrEP prescription filled 29 days before first detectable VL. This was later identified to be a clinical miss, as the individual had acute HIV seroconversion at PrEP initiation and was diagnosed at one month follow-up. Nearest phylogenetic neighbours to viruses with M184I/V did not have M184I/V (Fig. 1B), suggesting it was unlikely to have been transmitted between hosts.

**Table 2. Proportion of newly diagnosed PrEP users and non-PrEP users from 2018-2022 with treatment selected mutations in *reverse transcriptase* (*rt*) and *protease* (*pro*).** Frequencies were compared using Fisher’s exact tests (as the assumption of minimum expected frequencies for chi-squared violated) and p-values were adjusted using Benjamini-Hochberg method. M184V alone and including M184IV are emboldened.

|  |  |  | **Newly Diagnosed PrEP users (n=39)** | | **Newly Diagnosed Non-PrEP users (n=566)** | | **Fisher's Test** | |
| --- | --- | --- | --- | --- | --- | --- | --- | --- |
| **Drug class** | **Mutation** | **Gene** | **Total** | **%** | **Total** | **%** | **Unadj. p** | **Adj. p** |
| Nucleoside analogue reverse  transcriptase inhibitor (NRTI) | M41L | *rt* | 1 | 2.56 | 10 | 1.77 | 0.5226 | 0.5226 |
|  | K70R | *rt* | 1 | 2.56 | 37 | 6.54 | 0.5012 | 0.5226 |
|  | V75I | *rt* | 1 | 2.56 | 2 | 0.35 | 0.1815 | 0.3327 |
|  | **M184V** | ***rt*** | **2** | **5.13** | **2** | **0.35** | **0.0224*** | **0.2463** |
|  | **M184IV/V** | ***rt*** | **3** | **7.69** | **2** | **0.35** | **0.0023*** | **0.0250*** |
|  | T215E | *rt* | 3 | 7.69 | 15 | 2.65 | 0.1031 | 0.2747 |
| Non-NRTI (NNRTI) | K103N | *rt* | 2 | 5.13 | 15 | 2.65 | 0.3007 | 0.4725 |
|  | K103S | *rt* | 1 | 2.56 | 8 | 1.41 | 0.4533 | 0.5226 |
|  | Y181C | *rt* | 1 | 2.56 | 9 | 1.59 | 0.4891 | 0.5226 |
|  | N348I | *rt* | 2 | 5.13 | 6 | 1.06 | 0.0886 | 0.2747 |
| Protease inhibitors (PI) | A71I | *pro* | 1 | 2.56 | 1 | 0.18 | 0.1249 | 0.2747 |
|  | L90M | *pro* | 1 | 2.56 | 1 | 0.18 | 0.1249 | 0.2747 |

**Figure 1. Baseline HIV drug resistance in newly diagnosed individuals with and without PrEP.** A) Proportion of newly diagnosed with or without PrEP use with baseline drug resistance mutations to NRTI, NNRTI, PI, and INSTI drug classes. Annotations indicate number of individuals with baseline resistance. Mutation M184I/V shaded in blue. B) Clustered viruses with baseline M184V or M184I/V. Subtrees pruned from maximum likelihood trees inferred for individual clusters in IQ-TREE 2.

**Transmission of resistance-associated mutations in phylogenetic clusters**

We evaluated whether baseline drug resistance mutations were found at high frequencies within clusters, suggestive of onward transmission in active outbreaks. There were 28 active clusters with at least one new diagnosis since 2018 with baseline resistance mutations (Fig. 2). NRTI RAM, K70R, and NNRTI RAM, K103N, were increasingly detected at baseline (Fig. S4) and transmitted within clusters (Fig. 2, Fig. S12). NRTI RAMs detected multiple times within clusters, suggesting possible transmission include thymidine analogue mutation (TAM) revertant T215E (100% of 14 new cases in cluster 114, Fig. S12), K70R (27.3% of 11 new cases in cluster 49, Fig. S9; 100% of 2 new cases in cluster 185; and outside clusters, Fig. 2), D67N (11.5% of 26 new cases in cluster 137, Fig. S13), and M41L (4.1% of 94 new cases in cluster 31, Fig. S8; present in clade of cluster 137, Fig. S13). Baseline NNRTI RAMs detected multiple times within clusters included K103N (2.1% of 94 new cases in cluster 31, Fig. S8; 80% of 5 new cases in cluster 97; 40% of 5 in cluster 194), K103S (83.3% of 6 new cases in cluster 234), Y181C (100% of 5 new cases in cluster 197), and N348I (85.7% of 7 new cases in cluster 41). No baseline K65R, conferring resistance to tenofovir, was detected among new cases.

**Figure 2. Baseline drug resistance mutations in newly diagnosed from 2018 to 222 in BC with or without PrEP use.** Restricted to oldest sequences from participants with any baseline drug resistance (n=154). Mutations sorted by gene, then codon position, and colored by PrEP use. Phylogeny is a bootstrap approximate maximum likelihood tree of ~40,000 partial pol sequences trimmed of drug resistance mutation codons, rooted on the oldest subtype B sequence, and pruned to newly diagnosed with any baseline drug resistance mutations; scale in substitutions per site. Integrase sequences were not available for all partial pol sequences.

**Reduction of HIV reproduction number following PrEP among GBM**

Within the DTP, HIV incidence declined from 9.2 new cases per 100,000 in 2012 to 2.7 new cases per 100,000 in 2022 (Fig. S15). Before PrEP, new HIV cases overall decreased annually by -12.1% in 2016 and -23.4% in 2017 (Fig. 3), with most rapid declines among PWID (-19.7% in 2016, -49.0% in 2017), but less so among GBM (-4.7% in 2016, -11.4% in 2017, Fig. S17). Following PrEP, annual new HIV cases in GBM declined -19.2% in 2018 and -8.9% in 2019, and GBM accounted for fewer new cases. During COVID-19, new HIV cases in GBM declined -26.1% in 2020, whereas they increased 6.4% among HET. Post-COVID, new HIV cases increased by 5.7% in GBM in 2022 likely due to increased potential exposures and testing, despite lowering -54.1% in PWID and -28.3% in HET. Among GBM, average R_e_ was around 1 before PrEP, then dropped below 1 during PrEP, and further below 1 during COVID-19 (Fig. 3, Fig. S22). R_e_ among PWID (Fig. S23) and HET were elevated following PrEP, increased during COVID-19, and have been relatively low post-COVID-19.

**Figure 3. HIV dynamics in BC from 2015 to 2023 in the context of PrEP and COVID-19.** **A)** Annual new HIV diagnosed cases (based on first ART date) overall and among key populations: gay, bisexual, and other men who have sex with men (GBM), heterosexuals (HET), and people who inject drugs (PWID). **B)** Annual percentage change in new HIV diagnoses. **C)** Average effective reproduction number (R_e_) for periods i) before PrEP (2016-2017), ii) during PrEP and before COVID-19 (2018-Feb. 2020), iii) during PrEP and during COVID-19 (Mar. 2020-2021), and iv) during PrEP and post-COVID-19 (2022). **D)** Temporal dynamics of R_e_ overall and in key populations.

**Differential cluster growth following availability of PrEP**

We evaluated heterogeneity in R_e_ dynamics in phylogenetic clusters following PrEP. There were 84 active clusters with at least one new case since 2018; 53 clusters had more than 10 members by Feb. 2023 (Fig. S1, Fig. S3). Of active clusters, 52 were predominantly GBM, 30 PWID, and 2 HET (Table S5).

Transmission within and outside BC HIV phylogenetic clusters was variably impacted by PrEP (Fig. 4, Fig. S24-30). Cluster 31, the largest GBM-predominant cluster (size=391, 89% GBM, 94 new cases since 2018), had stable R_e_ near 1, followed by an increase in mid-2017, then a decline since PrEP availability (fold-change (FC) R_e_ with PrEP<1). Other GBM-predominant clusters (cluster 95, size=158, 27 new cases; and cluster 22, size=42, 19 new cases) had sustained declines through PrEP, with recent resurgences post-COVID-19 (Fig. S29). The two largest PWID-predominant clusters (clusters 49 and 57, sizes 459 and 471, 91% and 86% PWID) have had limited new cases since 2018 (11 and 19). R_e_ of PWID cluster 57 was stably below or near 1 from 2015 to 2018, then rose significantly above 1 in 2019, coming down in 2020 before rising above 1 post-COVID-19. PWID cluster 137 (size=90, 26 new cases, 88% PWID) has also had volatile R_e_, peaking in 2017-2018, dropping in 2019, and rising again in late 2019 and 2020. Comparing the PrEP effect on cluster-level R_e_ in key populations, 50% of GBM clusters had reduced R_e_ following PrEP, compared to 33.3% of PWID clusters (Fig. 4).

**Figure 4. HIV phylogenetic cluster reproduction number in the context of PrEP and COVID-19.** **A)** Phylogenetic cluster-level effective reproduction number (R_e_) for eight key active clusters in BC from 2015 to 2022. R_e_ estimated using EpiEstim with gamma-distributed serial interval with mean 1 y and sd 0.5 y, estimating window 0.5 y, and 90 d smoothing. **B)** Piecewise average R_e_ for active clusters in periods before PrEP, during PrEP and before COVID-19, during PrEP and during COVID-19, and during PrEP and post-COVID-19. Annotated by arbitrary cluster identifier, colored by predominant risk group, and area by cluster size in 2022. **C)** Fold-change (during/before) in active clusters’ R_e_ following PrEP. Clusters sorted on the x-axis by predominant risk group and size in 2022.

**Differential diagnoses averted via PrEP in phylogenetic clusters**

Diagnoses averted in phylogenetic clusters via PrEP between 2018 and 2022 were estimated using stochastic branching processes (Table S6, Fig. S31-S33)(38–40). Diagnoses averted were calculated as the difference between new diagnoses without and with PrEP simulated with cluster R_e_ specified as observed R_e_ (with PrEP) or adjusted R_e_ (counterfactual without PrEP).

PrEP averted the most diagnoses in large and medium sized GBM-predominant clusters, including clusters 31, 22, 245, and 33 (Fig. 5, Fig. S33). In cluster 31, we estimated 86.8 new diagnoses (95% CI 84.2-89.4) from 2018-2022 in simulations based on observed R_e_ (with PrEP, Fig. S31), consistent with the observed 94 new sequenced diagnoses; without PrEP, we estimated up to 115.7 (112.3-119.1) new diagnoses (Fig. S33), amounting to 28.9 (28.1-29.7) diagnoses averted in cluster 31 in five years. In cluster 22, there were 24.0 (21.7-26.4) new diagnoses with PrEP (consistent with 19 new diagnoses since 2018), compared to 49.9 (45.9-54.1) without PrEP, amounting to 25.9 (24.2-27.7) diagnoses averted. PrEP was also associated with reduced R_e_ in two medium-sized, relatively young, GBM-predominant clusters with one new diagnosis since 2018 (cluster 245, size 2023=13, median age=34; cluster and 33, size 2023=10, median age=37), dropping from R_e_ well above 1 before PrEP to below 1 during PrEP. We estimated 20.0 (17.9-22.1) diagnoses averted in cluster 245 and 6.0 (5.2-64.0) diagnoses averted in cluster 33. In total, among active phylogenetic clusters in BC with HIV diagnoses averted, we estimated 107.6 (99.1-115.7) diagnoses averted via PrEP in BC between Jan. 2018 and Jan. 2023, or 21.5 (19.8-23.1) diagnoses averted per year.

**Figure 5. Diagnoses averted via PrEP in active phylogenetic clusters estimated using stochastic branching processes. A)** Simulations of new diagnoses in clusters from January 2018 to January 2023 were run with observed cluster R_e_ (with PrEP since 2018) or with R_e_ adjusted by the estimated PrEP effect (no PrEP counterfactual scenario). **B)** Diagnoses averted by PrEP from 2018 to 2022 by cluster, estimated as the difference between new diagnoses in simulations with and without PrEP. Bar height represents the mean and error bars represent the bootstrap 95% CI across simulations. Cluster 9999 represents non-clustered cases.

Clusters with negative diagnoses averted represent missed opportunities for PrEP and include PWID-predominant clusters 137, 205, and 49, as well as non-clustered cases (cluster 9999, predominantly GBM but mixed) and GBM-predominant clusters 142, 234, and 13. Cluster characteristics univariately associated with fewer diagnoses averted include higher median age, lower proportion of new diagnoses with PrEP access, and living outside of Vancouver Coastal Health Authority. For instance, cluster 142 is comprised of relatively older individuals (median age=55, median age first ART=43), with a majority of new diagnoses in Vancouver Island Health Authority (Table S5). In a Poisson model of diagnoses averted, clusters with higher median age and lower % GBM had significantly fewer diagnoses averted, adjusted for cluster size at the end of 2017 and % residing in Vancouver Coastal Health Authority (Table S7).

Assuming the overall lifetime cost associated with each HIV diagnosis is $250,000 Canadian dollars (35), 107.6 (99.1-115.7) diagnoses averted via PrEP in BC between Jan. 2018 and Jan. 2023 represents an averted lifetime treatment cost of $26,900,000 Canadian dollars ($24,775,000 - $28,925,000). However, cost-effectiveness analyses must weigh potential benefits of prioritized PrEP programs against the costs including drugs (estimated as $360 USD per person per year in the US), clinic visits ($104 USD), diagnostic tests, and administrative hours (36).

**Discussion**

Our analyses suggest publicly funded PrEP in BC, alongside generalized access to free ART under the TasP strategy, has substantially reduced morbidity by averting more than 100 new diagnoses since 2018, while contributing to limited baseline NRTI drug resistance in instances where PrEP was prescribed or taken inadvertently during acute infection. While PrEP has dampened transmission most notably in large GBM-predominant clusters, other clusters have had fewer cases averted, illuminating treatment and prevention gaps.

The widespread availability of PrEP has impacted phylogenetic clustering patterns in multiple ways. We found newly diagnosed PrEP users were more likely to join clusters than non-PrEP users, which could reflect systematic differences in health-seeking and risk-taking behaviour among those who accessed PrEP. In other words, PrEP users may be subject to risk compensation (Powell 2019), but also may get tested more frequently and diagnosed earlier (which higher median CD4+ count, Table S3, suggests); both of these characteristics could lead to higher clustering. Furthermore, the impact of PrEP on reducing transmission in clusters representing active outbreaks has been heterogeneous, based on differential education, access, retention, or adherence across populations at risk of infection. We highlighted fewer cases averted via PrEP in clusters with higher median age, lower proportion GBM, and low proportion of previous PrEP use. PrEP uptake, adherence, and retention differ by age, risk perception (42), geography, social norms, substance use, ease of refills, community support, and stigma. Newly diagnosed PrEP users represent missed opportunities for program retention through better tools for support, such as peer workers or long-acting options. Whereas newly diagnosed non-PrEP users represent a missed opportunity for PrEP education and access. We further highlight that contacts of members of active phylogenetic clusters represent ongoing opportunities for PrEP education, eligibility, and access. Among HIV-negative GBM not using PrEP in Vancouver in 2018, 28% were uncomfortable asking doctors for PrEP, highlighting the importance of educating physicians as PrEP providers (42), and exploring potential for alternative care delivery models such as online PrEP services to improve PrEP access and retention.

Newly diagnosed PrEP users were more likely to have baseline M184I/V conferring reduced susceptibility to NRTIs, FTC, 3TC, and ABC, and individuals with M184I/V had a short time since last PrEP dispensation. This suggests inadvertent PrEP exposure during acute infection may have selected for M184V, or prevented the reversion of M184I/V-containing variants, potentially limiting treatment options with regimens containing 3TC or FTC (43) Baseline M184I/V was found among individuals with more recent PrEP dispensations and in one instance occurred in an individual with acute HIV infection (false negative) during PrEP initiation. Phylogenetic neighbours of PrEP users with baseline M184V did not carry the mutation, suggesting it was acquired *de novo*, although we cannot exclude the possibility it may have been an undetected minority variant or had reverted to wild-type prior to testing in these individuals. PrEP clients likely undergo more frequent HIV diagnostic testing, raising the possibility of earlier enrolment in the DTP and earlier drug resistance testing, which could impact the likelihood of detecting M184V. We lack evidence of onward transmission of M184I/V, corroborating its fitness costre by the time baseline drug resistance testing occurs.

In the Swiss HIV epidemic, M184V along with RAMs, D67N, K70R, and K219Q conferred negative fitness, while L90M was advantageous (44) In the absence of drug selection pressure, M184V was rapidly lost in a UK cohort, whereas T215 revertants, D67N, L210W, and K219Q/N persisted (45). We reported probable transmission of RAMs within clusters with clinical implications, as K70R reduces susceptibility to AZT and K103N reduces susceptibility to nevirapine and efavirenz (27). Detection of T215E TAM revertant mutation, in 100% of 14 new cases within a key cluster could suggest transmission of a resistant variant that is in the process of reverting to wild-type.

R_e_ estimates depend on the serial interval, i.e., the time between successive infections’ symptom onset (46). Compared to viruses with short period of infectiousness, HIV serial intervals can vary widely, as transmission could occur during acute infection (45% of transmissions in the first year in a Detroit MSM cohort) (47) or later if there is viral rebound. We explored realistic serial intervals, settling on 1 y for the primary analysis under the premise of widely available testing and treatment in BC. Our estimates assume that serial interval is comparable across clusters and key populations. The cluster-specific R_e_ confidence intervals and uncertainty were wide when newly diagnosed cluster members were sparse (Fig. 4, Fig. S24). Although we filtered estimates with CI width greater than 10, a more stringent cut-off or wider estimating window may have been warranted in some instances. Reducing noise in time-series data could improve R_e_ estimates for low incidence (48), which we circumvented with wide estimation windows and smoothing. Circumstantial changes could confound temporal dynamics. For instance, COVID-19 lockdown in 2020 reduced availability of treatment and prevention resources, but also reduced social activity and testing, altogether resulting in elevated growth of some PWID clusters (49). Although temporal trends were robust to serial intervals, R_e_ estimates remain uncertain due to noise, diagnosis delay, and incomplete sampling.

PrEP has contributed to reducing HIV transmission in BC, alongside interventions such as combination ART (50,51), TasP (52,53), and safe injection sites (54) Ongoing evaluations of the effectiveness of interventions including PrEP access, education, and delivery modes in clusters and key populations contribute towards evidence for intervention adoption in other jurisdictions, and to improve the efficiency of treatment and prevention resources locally. Our findings highlight the success of layering PrEP aimed to individuals at high risk of HIV infection on top of generalized free access to ART, under the BC TasP strategy, to optimize the control of HIV/AIDS. At the same time, longitudinal phylogenetic monitoring offers the unique advantage of identifying individuals and groups that could benefit from prioritized ART and prevention education, access, and support.

**Acknowledgments**

We thank Drug Treatment Program participants and staff, as well as colleagues in the BC-CfE Laboratory. AM was supported by a doctoral grant from the Canadian Institutes of Health Research and the BC-CfE. JSGM is supported by grants paid to his institution by the British Columbia Ministry of Health, Health Canada, Public Health Agency of Canada, Vancouver Coastal Health, Vancouver General Hospital Foundation, and Genome British Columbia. JBJ is supported by the Canadian Institutes of Health Research, Genome British Columbia, Genome Canada, the Public Health Agency of Canada, and the BC-CfE. VM, PS, JT, MH, CJB, and RB were all supported by the BC-CfE.

**Author Contributions**

AM and JBJ conceived of the study and AM designed the study. JT, PS, and CJB facilitated the data request. AM conducted analyses, generated figures, and wrote the first version of the draft. JT, CJB, and JBJ provided methodological feedback. JT, VM, PS, JT, MH, CJB, RB, JSGM, and JBJ provided edits. JBJ, JSGM, and AM received funding for the study. AM and JBJ had access to data.

**Competing Interest Statement**

JSGM received institutional grants from Gilead Sciences, Merck, and ViiV Healthcare. CJB has received grants and honoraria as speaker and consultant paid to his institution from Merck, Gilead Sciences and ViiV Healthcare. All other authors declare no competing interests.

**References**

1. British Columbia Centre for Disease Control. HIV in British Columbia: Annual Surveillance Report 2017. Retrieved from <http://www.bccdc.ca/health-professionals/data-reports/hiv-aids-reports> [Internet]. 2019. Available from: <http://www.bccdc.ca/resource-gallery/Documents/Statistics%20and%20Research/Statistics%20and%20Reports/STI/HIV_Annual_Report_2017_FINAL.pdf>

2. Grant RM, Lama JR, Anderson PL, McMahan V, Liu AY, Vargas L, et al. Preexposure Chemoprophylaxis for HIV Prevention in Men Who Have Sex with Men. New Engl J Medicine. 2010;363(27):2587–99.

3. Baeten JM, Donnell D, Ndase P, Mugo NR, Campbell JD, Wangisi J, et al. Antiretroviral Prophylaxis for HIV Prevention in Heterosexual Men and Women. New Engl J Medicine. 2012;367(5):399–410.

4. Choopanya K, Martin M, Suntharasamai P, Sangkum U, Mock PA, Leethochawalit M, et al. Antiretroviral prophylaxis for HIV infection in injecting drug users in Bangkok, Thailand (the Bangkok Tenofovir Study): a randomised, double-blind, placebo-controlled phase 3 trial. Lancet. 2013;381(9883):2083–90.

5. Jenness SM, Maloney KM, Smith DK, Hoover KW, Goodreau SM, Rosenberg ES, et al. Addressing Gaps in HIV Preexposure Prophylaxis Care to Reduce Racial Disparities in HIV Incidence in the United States. Am J Epidemiology. 2019;188(4):743–52.

6. Corneli AL, Deese J, Wang M, Taylor D, Ahmed K, Agot K, et al. FEM-PrEP. JAIDS. 2014;66(3):324–31.

7. Toy J, Espinoza R, Trigg J, Shen T, Sereda P, Ready E, et al. Real-world utilization of HIV PrEP medication in a population-level PrEP program in British Columbia, Canada. CROI 2024 Poster. 2024.

8. Biospace. Health Canada Issues Notice Of Compliance For Gilead’s Truvada For Reducing The Risk Of Sexually Acquired HIV Infection [Internet]. 2016. Available from: <https://www.biospace.com/article/releases/health-canada-issues-notice-of-compliance-for-gilead-s-truvada-for-reducing-the-risk-of-sexually-acquired-hiv-infection-/>

9. Health Canada. Product information - APO-EMTRICITABINE-TENOFOVIR [Internet]. 2023 [cited 2023 Nov 29]. Available from: <https://health-products.canada.ca/dpd-bdpp/info?lang=eng&code=93840>

10. British Columbia Centre for Excellence in HIV/AIDS. Guidance for the use of pre-exposure prophylaxis (PrEP) for the prevention of HIV acquisition in British Columbia. 2020. Available from: <https://bccfe.ca/bccfe-documents/guidance-for-the-use-of-pre-exposure-prophylaxis-prep-for-the-prevention-of-hiv-acquisition-in-british-columbia/>

11. Toy KJ, Trigg J, Zhang WW, Sereda P, Lima VD, Lepik KJ, et al. Rapid uptake of HIV pre-exposure prophylaxis (PrEP) in a publicly funded population-based program in British Columbia, Canada. CROI 2019 poster [Internet]. 2019 Feb 27;956:1–1. Available from: <https://2jg4quetidw2blbbq2ixwziw-wpengine.netdna-ssl.com/wp-content/uploads/sites/2/posters/2019/1430_Toy_0956.pdf>

12. Lima VD, Zhu J, Card KG, Lachowsky NJ, Chowell-Puente G, Wu Z, et al. Can the combination of TasP and PrEP eliminate HIV among MSM in British Columbia, Canada? Epidemics. 2021;35:100461.

13. Lima VD, Zhu J, Barrios R, Toy J, Joy JB, Williams BG, et al. Longitudinal evolution of the HIV effective reproduction number following sequential expansion of treatment as prevention and pre-exposure prophylaxis in British Columbia, Canada: a population-level programme evaluation. Lancet HIV. 2024;11(7):E461–9.

14. Irvine MA, Salway T, Grennan T, Wong J, Gilbert M, Coombs D. Predicting the impact of clustered risk and testing behaviour patterns on the population-level effectiveness of pre-exposure prophylaxis against HIV among gay, bisexual and other men who have sex with men in Greater Vancouver, Canada. Epidemics. 2020 Mar 1;30:100360.

15. Grulich AE, Guy R, Amin J, Jin F, Selvey C, Holden J, et al. Population-level effectiveness of rapid, targeted, high-coverage roll-out of HIV pre-exposure prophylaxis in men who have sex with men: the EPIC-NSW prospective cohort study. Lancet HIV. 2018;5(11):e629–37.

16. Roberts DA, Bridenbecker D, Haberer JE, Barnabas RV, Akullian A. The impact of prevention‐effective PrEP use on HIV incidence: a mathematical modelling study. J Int AIDS Soc. 2022;25(11):e26034.

17. Doyle CM, Milwid RM, Cox J, Xia Y, Lambert G, Tremblay C, et al. Population‐level effectiveness of pre‐exposure prophylaxis for HIV prevention among men who have sex with men in Montréal (Canada): a modelling study of surveillance and survey data. J Int AIDS Soc. 2023;26(12):e26194.

18. Zhao R, Fairley CK, Cook AR, Phanuphak N, He S, Tieosapjaroen W, et al. Optimising HIV pre-exposure prophylaxis and testing strategies in men who have sex with men in Australia, Thailand, and China: a modelling study and cost-effectiveness analysis. Lancet Glob Heal. 2024;12(2):e243–56.

19. Jenness SM, Goodreau SM, Rosenberg E, Beylerian EN, Hoover KW, Smith DK, et al. Impact of the Centers for Disease Control’s HIV Preexposure Prophylaxis Guidelines for Men Who Have Sex With Men in the United States. J Infect Dis. 2016;214(12):1800–7.

20. Marcus JL, Hurley LB, Hare CB, Nguyen DP, Phengrasamy T, Silverberg MJ, et al. Preexposure Prophylaxis for HIV Prevention in a Large Integrated Health Care System. JAIDS. 2016;73(5):540–6.

21. Misra K, Huang JS, Udeagu CCN, Forgione L, Xia Q, Torian LV. Pre-exposure prophylaxis (PrEP) use history in people with antiretroviral resistance at HIV diagnosis: Findings from New York City HIV surveillance and partner services, 2015-2022. Clin Infect Dis. 2023;ciad699.

22. Dimitrov DT, Boily MC, Hallett TB, Albert J, Boucher C, Mellors JW, et al. How Much Do We Know about Drug Resistance Due to PrEP Use? Analysis of Experts’ Opinion and Its Influence on the Projected Public Health Impact. PLOS ONE. 2016;11(7):e0158620.

23. Powell VE, Gibas KM, DuBow J, Krakower DS. Update on HIV Preexposure Prophylaxis: Effectiveness, Drug Resistance, and Risk Compensation. Curr Infect Dis Rep. 2019;21(8):28.

24. Gibas KM, Berg P van den, Powell VE, Krakower DS. Drug Resistance During HIV Pre-Exposure Prophylaxis. Drugs. 2019;79(6):609–19.

25. Fonner VA, Dalglish SL, Kennedy CE, Baggaley R, O’Reilly KR, Koechlin FM, et al. Effectiveness and safety of oral HIV preexposure prophylaxis for all populations. AIDS. 2016; 30:1973–1983.

26. Poon A, Gustafson R, Daly P, Zerr L, Demlow SE. Near real-time monitoring of HIV transmission hotspots from routine HIV genotyping: an implementation case study. The Lancet HIV. 2016;3(5):e231–8.

27. Liu TF, Shafer RW. Web Resources for HIV Type 1 Genotypic-Resistance Test Interpretation. Clin Infect Dis. 2006;42(11):1608–18.

28. Price MN, Dehal PS, Arkin AP. FastTree 2--approximately maximum-likelihood trees for large alignments. Poon AFY, editor. PLoS ONE. 2010 Mar 10;5(3):e9490.

29. Hoang DT, Chernomor O, Haeseler A von, Minh BQ, Vinh LS. UFBoot2: Improving the Ultrafast Bootstrap Approximation. Mol Biol Evol. 2018;35(2):518–22.

30. Minh BQ, Schmidt HA, Chernomor O, Schrempf D, Woodhams MD, Haeseler A von, et al. IQ-TREE 2: New Models and Efficient Methods for Phylogenetic Inference in the Genomic Era. Mol Biol Evol. 2020;37(5):1530–4.

31. Paradis E, Schliep K. ape 5.0: an environment for modern phylogenetics and evolutionary analyses in R. Bioinformatics. 2018;35(3):526–8.

32. Statistics Canada. Table 17-10-0009-01  Population estimates, quarterly [Internet]. 2023 [cited 2023 Nov 20]. Available from: <https://www150.statcan.gc.ca/t1/tbl1/en/tv.action?pid=1710000901>

33. Kamvar ZN, Cai J, Pulliam JRC, Schumacher J, Jombart T. Epidemic curves made easy using the R package incidence. F1000Research. 2019;8:139.

34. Cori A, Ferguson NM, Fraser C, Cauchemez S. A New Framework and Software to Estimate Time-Varying Reproduction Numbers During Epidemics. American Journal of Epidemiology. 2013;178(9):1505–12.

35. Campbell AR, Kinvig K, Côté HC, Lester RT, Qiu AQ, Maan EJ, et al. Health Care Provider Utilization and Cost of an mHealth Intervention in Vulnerable People Living With HIV in Vancouver, Canada: Prospective Study. JMIR mHealth uHealth. 2018;6(7):e152.

36. Amick AK, Eskibozkurt GE, Hosek SG, Flanagan CF, Landovitz RJ, Jin EY, et al. Daily Oral HIV Pre-exposure Prophylaxis Among Young Men Who Have Sex With Men in the United States: Cost-saving at Generic Drug Price. Clin Infect Dis. 2023;78(2):402–10.

37. Ferreira JA, Zwinderman AH. On the Benjamini–Hochberg method. Ann Stat. 2006;34(4):1827–49.

38. Althaus CL. Ebola superspreading. Lancet Infect Dis. 2015;15(5):507–8.

39. Riou J, Althaus CL. Pattern of early human-to-human transmission of Wuhan 2019 novel coronavirus (2019-nCoV), December 2019 to January 2020. Eurosurveillance. 2020;25(4):2000058.

40. Lloyd-Smith JO, Schreiber SJ, Kopp PE, Getz WM. Superspreading and the effect of individual variation on disease emergence. Nature. 2005;438(7066):355–9.

41. Bennett DE, Camacho RJ, Otelea D, Kuritzkes DR, Fleury H, Kiuchi M, et al. Drug Resistance Mutations for Surveillance of Transmitted HIV-1 Drug-Resistance: 2009 Update. PLOS ONE. 2009;4(3):e4724-8.

42. Lachowsky NJ, Tattersall TL, Sereda P, Wang C, Edwards J, Hull M. Community awareness of, use of and attitudes towards HIV pre-exposure prophylaxis (PrEP) among men who have sex with men in Vancouver, Canada: preparing health promotion for a publicly funded PrEP program. Sex Health. 2019;1–7.

43. Rhee SY, Liu T, Ravela J, Gonzales MJ, Shafer RW. Distribution of Human Immunodeficiency Virus Type 1 Protease and Reverse Transcriptase Mutation Patterns in 4,183 Persons Undergoing Genotypic Resistance Testing. Antimicrob Agents Chemother. 2004;48(8):3122–6.

44. Yang WL, Kouyos RD, Böni J, Yerly S, Klimkait T, Aubert V, et al. Persistence of Transmitted HIV-1 Drug Resistance Mutations Associated with Fitness Costs and Viral Genetic Backgrounds. PLoS Pathog. 2015;11(3):e1004722.

45. Castro H, Pillay D, Cane P, Asboe D, Cambiano V, Phillips A, et al. Persistence of HIV-1 Transmitted Drug Resistance Mutations. J Infect Dis. 2013;208(9):1459–63.

46. Wallinga J, Lipsitch M. How generation intervals shape the relationship between growth rates and reproductive numbers. Proc R Soc B: Biol Sci. 2007;274(1609):599–604.

47. Volz EM, Ionides E, Romero-Severson EO, Brandt MG, Mokotoff E, Koopman JS. HIV-1 Transmission during Early Infection in Men Who Have Sex with Men: A Phylodynamic Analysis. PLOS Medicine. 2013;10(12):e1001568-12.

48. Parag KV. Improved estimation of time-varying reproduction numbers at low case incidence and between epidemic waves. PLoS Comput Biol. 2021;17(9):e1009347.

49. Miller RL, McLaughlin A, Montoya V, Toy J, Stone S, Harding J, et al. Impact of SARS-CoV-2 lockdown on expansion of HIV transmission clusters among key populations: A retrospective phylogenetic analysis. Lancet Regional Heal - Am. 2022;100369.

50. Hogg RS, Rhone SA, Yip B, Sherlock C, Conway B, Schechter MT, et al. Antiviral effect of double and triple drug combinations amongst HIV-infected adults: lessons from the implementation of viral load-driven antiretroviral therapy. AIDS. 1998;12(3):279–84.

51. Montaner JSG, Lima VD, Harrigan PR, Lourenço L, Yip B, Nosyk B, et al. Expansion of HAART coverage is associated with sustained decreases in HIV/AIDS morbidity, mortality and HIV transmission: the “HIV Treatment as Prevention” experience in a Canadian setting. PLOS One. 2014;9(2):e87872.

52. Montaner JS, Hogg R, Wood E, Kerr T, Tyndall M, Levy AR, et al. The case for expanding access to highly active antiretroviral therapy to curb the growth of the HIV epidemic. The Lancet. 2006;368(9534):531–6.

53. Lima VD, Brumme ZL, Brumme C, Sereda P, Krajden M, Wong J, et al. The Impact of Treatment as Prevention on the HIV Epidemic in British Columbia, Canada. Current HIV/AIDS Reports. 2020;1–11.

54. Tyndall MW, Wood E, Zhang R, Lai C, Montaner JSG, Kerr T. HIV seroprevalence among participants at a Supervised Injection Facility in Vancouver, Canada: implications for prevention, care and treatment. Harm Reduction Journal. 2006;3:36.
