# Supplementary material for: Heterogeneous impacts of HIV pre-exposure prophylaxis (PrEP) on drug resistance and phylogenetic cluster transmission dynamics in British Columbia, Canada: A retrospective cohort and simulation study
Source: PLoS Med. 2025 Dec 9;22(12):e1004827. doi: 10.1371/journal.pmed.1004827 (PMC12700434; doi:10.1371/journal.pmed.1004827)
Supplement: Attachment — Submitted filename: Responses_to_reviews_McLaughlin_PLOS_JBJ_AM_0825.docx [file pmed.1004827.s007.docx]

Dear Dr. Farrell,

Thank you for coordinating review of our manuscript and we are grateful to the reviewers for their thoughtful suggestions and clarifications which, we feel, have helped us substantially improve our manuscript. As such, we have responded to comments and suggestions below in blue, with **page and line references in bold** as well as manuscript excerpts.

**PMEDICINE-D-25-01591R1 Decision - [EMID:498191a6f8d4ebd6]**

Dear Dr Joy,

Many thanks for submitting your manuscript "Impact of pre-exposure prophylaxis on HIV drug resistance and phylogenetic cluster growth" (PMEDICINE-D-25-01591R1) to PLOS Medicine. The paper has been reviewed by subject experts and a statistician; their comments are included below and can also be accessed here: https://www.editorialmanager.com/pmedicine/l.asp?i=1015166&l=JK7GNCQO. As you will see, the reviewers find the study interesting, but raise important points that must be addressed in a revised manuscript. **In particular, reviewer 1 requires additional analyses, reviewers 2 and 3 require clarification, justification and support for your definition of transmission clusters, and reviewer 3 is concerned that the conclusions may not be generalizable in view of the low number of HIV infections among PrEP users.** The editors require that you revise the presentation to better **highlight the novel insights derived from the study and how they might guide public or clinical health interventions**. Please also ensure that the language in the manuscript adheres to PLOS guidelines and is **person-centered and non-stigmatizing**. After discussing the paper with the editorial team and an academic editor with relevant expertise, I'm pleased to invite you to revise the paper in response to the reviewers' comments. We plan to send the revised paper to some or all of the original reviewers, and we cannot provide any guarantees at this stage regarding publication. We will also seek the input of an independent statistician on a revised manuscript.

In addition to these revisions, you **may need to complete some formatting changes**, which you will receive in a follow up email. A member of our team will be in touch with a set of requests shortly. If you do not receive a separate email within a few days, please assume that checks have been completed, and no additional changes are required.

When you upload your revision, please include a **point-by-point response** that addresses all of the reviewer and editorial points, **indicating the changes made in the manuscript and either an excerpt of the revised text or the location (eg: page and line number)** where each change can be found. Please also be sure to **check the general editorial comments at the end of this letter** and include these in your point-by-point response. When you resubmit your paper, **please include a clean version of the paper as the main article file and a version with changes tracked as a marked-up manuscript**. It may also be helpful to check the guidelines for revised papers at http://journals.plos.org/plosmedicine/s/revising-your-manuscript for any that apply to your paper.

We ask that you **submit your revision by Aug 28 2025** 11:59PM. However, if this deadline is not feasible, please contact me by email, and we can discuss a suitable alternative.

Don't hesitate to contact me directly with any questions (afarrell@plos.org).

Best regards,

Alison

Alison Farrell, Ph.D.

-----------------------------------------------------------

Comments from the reviewers:

**Reviewer #1:** The study examines the effect of PrEP introduction on the individual-level (i.e. emergence of drug resistance) and on population-level HIV transmission dynamics. Using HIV molecular epidemiology, the authors show that newly-diagnosed PrEP users were more likely to join existing clusters and were more likely to have M184V DRM. Using cluster-level Re inference, the authors estimated the number of new HIV infections averted due to PREP and also present RE changes over time in clusters comprising various key populations living with HIV. The authors innovatively use molecular epi to show the differential effects of PrEP on new infections in different key populations. Overall, this paper will make a strong contribution to public health knowledge, I only have a few comments to help improve it.

My main concerns are:

1)	Some of the observed elevated clustering is expected in higher-risk individuals that would be eligible for PrEP. Instead of comparing characteristics in newly-diagnosed PrEP users to all non PrEP users, a comparison to those non PrEP users who were eligible for PrEP could be more fitting.

Thank you for this suggestion. Although we do not have access to PrEP eligibility data such as sexual behaviour to inform PrEP eligibility, nearly all eligible individuals who accessed PrEP were GBM. Therefore, to address this we added a comparison of the proportion clustering among GBM newly diagnosed PrEP users to newly diagnosed GBM non-PrEP users, in addition to comparing to all newly diagnosed non-PrEP users.

**Page 5 Lines 150-153:** “We tested the hypotheses that newly diagnosed PrEP users were more likely to join phylogenetic clusters in BC than newly diagnosed non-PrEP users generally and among GBM, under the expectation that PrEP eligibility and use is related to behaviour and linkage to sexual transmission networks”

**Page 8 Lines 304-312:** “Newly diagnosed PrEP users were significantly more likely to cluster than non-PrEP users in the entire cohort (chi-squared test p=0.0075, Fig. S2), and among GBM (p=0.005). Of 39 newly diagnosed PrEP users, 30 joined 13 active clusters (76.9% clustered, one-group proportions test 95% confidence interval (CI): 60.3-88.3%), compared to 303 of 566 non-PrEP users (53.5% clustered, 95% CI: 49.3-57.7%), who joined 84 active clusters (Table 1). Among those identifying as GBM, 28/34 (82.4%, 95% CI: 64.8-92.6%) of newly diagnosed PrEP users joined phylogenetic clusters, compared to 145/265 (54.7%, 95% CI: 48.5-60.8%) of non-PrEP users (Table S4; chi-squared test p=0.005).”

2)	The number of averted infections in clusters was simulated using the cluster definition of 5+ infections and the observed cluster distribution. I suggest some sensitivity analysis on cluster size distribution for this analysis, or at least specify/discuss that the observed is likely the lower bound of the number of averted infections.

To accommodate this request we have added material to the discussion on the impact of cluster definition on our estimations of averted infections. Our ethics approval disallows studying smaller clusters to protect patient confidentiality.

**Page 19 Line 567-575:** “Patient confidentiality also dictates that phylogenetic clusters must comprise at least five individuals, which prevents the identification of small clusters that are instead grouped into a pseudo cluster representing all non-clustered individuals for simulations. The pseudo cluster represents a heterogeneous population of PLWH, for which some subpopulations may have underestimated cases averted if there was more growth potential in the absence of PrEP than modelled (small GBM clusters), while other subpopulations may have had overestimated cases averted if new cases were similarly low with or without PrEP (virally suppressed recent migrants, for example). Stratifying the non-clustered population into more homogeneous subpopulations could improve model accuracy.”

Some other minor comments:

1. Line 108 - please clarify whether the hypothesis was focused on any clustering, or clustering within the PrEP users networks.

Corrected.

**Page 5 Lines 150-153:** “We tested the hypotheses that newly diagnosed PrEP users were more likely to join phylogenetic clusters in BC than newly diagnosed non-PrEP users generally and among GBM, under the expectation that PrEP eligibility and use is related to behaviour and linkage to sexual transmission networks”

**Page 6 Lines 219-221**: “We tested the hypotheses that newly diagnosed PrEP users included a higher proportion of clustered individuals and higher proportion of baseline NRTI resistance than non-PrEP users overall and among GBM”

2. Cost analysis seems redundant as the authors mention that it does not take the alternative costs (i.e. cost of Prep) into account. As is seems like an unnecessary addition to the paper.

We appreciate the suggestion and have removed this section from the results and methods. However, we retained this in the discussion to suggest future research towards a cost-benefit analysis.

**Page 20 Lines 635-639:** “Future research should consider a comprehensive cost-benefit analysis of widespread PrEP availability, incorporating benefits based on diagnoses averted as estimated herein, the approximate lifetime cost of each HIV diagnosis, estimated as $250,000 Canadian dollars in 2018 [58], as well as costs for drugs, clinic visits, diagnostic tests, and administrative hours [59].”

3. Higher diversification rates are also expected in PrEP users since they are more likely to be diagnosed during acute infection.

This is an interesting confounder to consider for clustering and diversification rate, and we appreciate the reviewer highlighting this. We have elaborated upon this in the discussion as follows:

**Page 17 Lines 499-506:** “Newly diagnosed PrEP users were more likely than non-PrEP users to join phylogenetic clusters and have higher viral diversification rates, which could reflect higher connectivity in the sexual network to some extent, but also systematic differences in risk-taking and health-seeking behaviour among those who accessed PrEP. HIV PrEP users may be subject to risk compensation [21], engaging in riskier sex and/or with more partners, but also may get tested more frequently and diagnosed earlier, as supported by their higher median CD4+ count (Table S3), both of which could lead to more frequent clustering and higher diversification rates.”

4. Would be helpful to mention whether nearest phylogenetic neighbours to non-PrEP users with M184I/V had M184I/V (to support that it's likely direct transmissions and we're not missing intermediates, which would help the point made for PrEP users)

We have reported this in the results with further elaboration:

**Page 9 Lines 347-350:** “Nearest phylogenetic neighbours to viruses with M184I/V sampled from five newly diagnosed with and without prior PrEP use did not have detectable M184I/V (Fig. 1B), suggesting it was unlikely to have been transmitted between hosts in any cases, but rather acquired *de novo*.”

5. Integrase DRM are in the table but not discussed in the text

We apologize for this omission. We have added to the results text to mention these:

**Page 11 Lines 379-381:** “No baseline INSTI RAMs were detected among newly diagnosed PrEP users, however five non-PrEP users carried five different baseline INSTI RAMs: E138A, E138K, H51Y, T66I, and Y143S (Fig. 1).”

6. Figure 1B would be more helpful with resolved polytomies

In this case, polytomies provide context for identical or highly alike sequences, particularly those only differing based on the presence of M184I/V.

7. Did you use any threshold to define clusters as "predominantly" GBM?

Thanks for this clarifying question. Detail has been added as follows:

**Page 14 Lines 419-421:** “Of active clusters, 52 were predominantly (i.e., comprising the largest proportion of any risk group) GBM, 30 PWID, and 2 HET (Table S5).”

8. Line 427 - does Prep access here mean Prep use or Prep eligibility?

We thank the reviewer for this important question. To clarify this, we have added the following sentence.

**Page 17 Lines 482-484:** “Cluster characteristics univariately associated with fewer diagnoses averted include higher median age, lower proportion of new diagnoses with PrEP use, and living outside of Vancouver Coastal Health Authority.”

**Reviewer #2:** From the methods it is not clear how authors defined clusters, as they mentioned that they used cutoff and bootstrap as indicated in PMC4853759, but not further steps from those methods to ensure proper cluster identification. Can authors use a phylogenetic cluster informed method like PhyloPart or genetic distance based HIV-trace to define clusters and compare with their method?

We appreciate the reviewer seeking clarity on the methodology choice. To address this and clarify we have expanded upon clustering methods in the methods section and corrected the citation for justification of this cut-off.

**Page 6 Lines 197-200:** “Phylogenetic clusters were identified as groups of at least five individuals whose viruses share a pairwise patristic (tree) distance <0.02 substitutions per site in greater than 90% of bootstraps [24]. This pairwise tree distance threshold was identified as the 95th percentile of within-host pairwise distances for subtype B in BC [24] and is used for phylogenetic monitoring in BC in the phylogenetic monitoring program.”

Furthermore, we have elaborated upon the sensitivity of our results to the cluster identification method in the discussion:

**Page 19 Lines 576-591:** “Phylogenetic cluster identification is sensitive to the pairwise tree or genetic distance threshold applied, as well as other methodological considerations like tree inference or bootstrap support. Clustering methodologies can lead to differential results and sensitivities to detecting differences in transmission and sampling [53]. While HIV clusters can be alternatively identified using a pairwise genetic TN93 distance near 1.5% through HIV-TRACE [54], the patristic distance methodology has the advantage of additionally considering phylogenetic topology with an underlying evolutionary model and bootstrap support. Beyond there being an established phylogenetic monitoring program in BC based on this method, we found phylogenetic clustering identified through patristic distance was concordant with viral lineage-level diversification rate towards evaluating risk factors for elevated transmission risk [27], could be prioritized using the diversification rate [29], and delineated populations most negatively impacted by service disruption during COVID-19 [28]. A comparison of cluster membership and size distribution across methods, applying different thresholds would be worthwhile. We demonstrate heterogeneity in clusters’ transmission dynamics that can be used to more granularly simulate epidemic scenarios; how those clusters are defined will vary by setting.”

It would be interesting to know how diverse PREP-users sequences are from non-PREP.

We thank the reviewer for this suggestion. We addressed this by comparing the lineage-level diversification rate distributions between these groups (Fig. S2), which is a tip-level metric that quantifies the reciprocal of how much phylogenetic distinctness a tip carries, i.e., how close is it to its neighbours.

**Page 8 Lines 312-314:** “Newly diagnosed PrEP users also had significantly higher lineage-level viral diversification rates, indicative of rapid, recently sampled transmission events, than non-PrEP users (Fig. S2).”

Since authors used longitudinal data per patient (sometimes up to 35 sequences per patient), are clusters comprised of mainly patient longitudinal data? How large were clusters containing more homogenous clusters (longitudinal patient data) compared to heterogenous clusters (diverse patients, no longitudinal data).

Thanks for seeking clarification here. We have adjusted the definition as follows:

**Page 6 Lines 197-199:** “Phylogenetic clusters were identified as groups of at least five individuals whose viruses share a pairwise patristic (tree) distance <0.02 substitutions per site in greater than 90% of bootstraps [24].”

To address the question of an association between cluster size and representation of patients with single vs. longitudinal samples, while all data are considered when evaluating cluster composition, when ascribing a cluster each individual is only represented once in a given cluster i.e. their longitudinal sequences are not included in the cluster which would inflate it’s size.

Another question about cluster definition, is whether the clusters are BC unique or are intermixed with sequences from other Canadian provinces or foreign countries. Can authors add GenBank sequences to check the monophyly of their clusters?

The extent to which BC clusters include other provinces and countries is a fascinating question within a challenging landscape of data privacy and sharing issues. While some anonymized data is publicly available, the likelihood is relatively low that they would include representative sequences tightly epidemiologically linked to the BC epidemic given those linked sequences could be from anywhere in the world. There are ongoing efforts in BC to collaborate across Canadian provinces and territories to conduct a study of this magnitude to understand how clusters are connected across jurisdictions. This has been added as a limitation of the study

**Page 18 Lines 557-567:** “Limitations in the identification of clusters and simulation of their growth include the inherent uncertainty in the phylogenetic reconstruction, exacerbated by the absence of undiagnosed individuals and those outside of BC. In this analysis, we focus on transmission between BC residents and assume a densely sampled local epidemic, which is supported by our finding that 77% and 54% of new diagnoses joined pre-existing clusters with and without PrEP, respectively. Since clusters are partially sampled, this could lead to underestimating their actual or simulated growth. Sharing anonymized HIV data with metadata across jurisdictions is challenging due to the need to protect patient privacy and confidentiality, therefore there is a need to develop secure and equitable data sharing agreements with protective measures to better understand HIV transmission dynamics on larger scales.”

What are the epidemiological, clinical and resistance profile of the 9 PREP sequences that non clustered? are these from a unique patient or 9 patients?

I believe the clarification in cluster definition addresses the latter question; there were 9 unique patients newly diagnosed with previous PrEP use.

The characteristics of the 9 newly diagnosed PrEP users that did not cluster can be pulled from Table S4, as the difference between the total population of newly diagnosed with previous PrEP and the clustered population (n. clust). To address this we have added a sentence summarizing this groups’ notable features in the results.

**Page 8 Lines 316-318:** “The nine non-clustered newly diagnosed previous PrEP users (difference between total and n clustered in Table S4) included a heterosexual male, and two minority subtypes A1 and 02_AG.”

Based on the results that PrEP users were more likely to have baseline M184I/V, what authors would suggest as improved/new guidelines to public health officials about the issue?

We are grateful to the reviewer for pointing out this omission. We have added a paragraph outlining our suggestions based on the study findings:

**Page 20 Lines 628-639:** “In light of the potential for selection of drug resistance mutation M184I/V and its negative impact on clinical outcomes [49], we recommend reconsidering first line ART regimens containing TDF or FTC if PrEP use is known or suspected during acute infection. We further recommend PrEP eligibility be widened to be inclusive for PWID, sex workers, and trans-inclusive language. Our cluster simulations suggested more cases could be prevented through improved education, access, and retention for PrEP for older individuals and those residing outside of Vancouver, for which prescribing physicians play a key role. Future research should consider a comprehensive cost-benefit analysis of widespread PrEP availability, incorporating benefits based on diagnoses averted as estimated herein, the approximate lifetime cost of each HIV diagnosis, estimated as $250,000 Canadian dollars in 2018 [58], as well as costs for drugs, clinic visits, diagnostic tests, and administrative hours [59].”

**Reviewer #3:** In this paper the authors compare newly diagnosed PrEP users (n=39) in British Columbia to newly diagnosed non-PrEP users (n=566) over the period of 2018-2022. Transmission clusters were identified by phylogenetic analysis and their composition analyzed to assess the efficacy of PrEP. The findings show that newly HIV diagnosed PrEP users were significantly more likely than non-PrEP users to join clusters and carry baseline nucleoside-analogue reverse transcriptase inhibitor (NRTI) resistance mutation M184I/V. The authors also show through simulation that PrEP averted approximately 20 new HIV diagnoses per year in British Columbia since 2018, and infrequently contributed to baseline drug resistance. Overall, the paper is well written and the findings provide an assessment of the efficacy of PrEP. However, the result is not novel, since many studies have shown that PrEP can effectively reduce the number of transmissions.

We appreciate that there have been a multitude of studies published on PrEP efficacy in clinical trials and real-world effectiveness towards reducing cases, however this study is novel in the cluster-level approach to estimating the effectiveness of PrEP. We have shown that effective reproduction numbers can differ widely between clusters, justifying a cluster-stratified counterfactual simulation of transmission in the presence or absence of PrEP. Similarly, this is not the first study to report elevated baseline M184V among newly diagnosed PrEP users with incomplete PrEP adherence (Misra et al. 2023, for example), however, this study is novel in its reporting of M184I/V among previous PrEP users in this geography and within the phylogenetic context to support their *de novo* appearance.

We have added to the conclusion to emphasize the novelty in this study’s approach to jointly inferring the individual and population level effects of PrEP on drug resistance and transmission.

**Page 20 Lines 640-646:** “Our findings highlight the success of PrEP towards reducing HIV transmission in BC, alongside interventions such as combination ART [60,61], BC TasP strategy to optimize the control of HIV/AIDS [62,63], and safe injection sites [64]. Ongoing phylogenetic monitoring of clusters provides an epidemiologically relevant delineation of subpopulations to evaluate differences in PrEP and other interventions’ effectiveness, investigate transmission of resistance associated mutations, as well as direct prioritized treatment and prevention resources.”

Additionally the study includes a relative low number of diagnosed PrEP users (n=39), which casts doubts on the robustness of the statistical results.

We recognize this concern regarding reduced the statistical power, which we reported.

**Page 9 Lines 336-340:** “Three of 39 newly diagnosed PrEP users (7.7%) had baseline M184V or M184IV (referred to collectively as M184I/V), compared to 2 of 566 (0.35%) non-PrEP users (Fisher’s exact test, adjusted p-value=0.025; Table 2; power=79% at alpha=0.05). M184I/V was the only baseline RAM significantly more common in PrEP users than non-PrEP users (Table 2).”

We have added this point to the limitations and future research sections.

**Page 18 Lines 549-557:** “There are several limitations to consider in these analyses, first in regards to our statistical comparisons of mutation frequencies between newly diagnosed with PrEP use (n=39) or without (n=566), which are unbalanced, reducing the statistical power. We calculated 79% power to detect true differences in baseline M184I/V frequencies between these groups with a 5% chance of rejecting a false null hypothesis. Ongoing monitoring of this cohort or pooling of data across jurisdictions would improve the statistical power of detecting differences in drug resistant mutation differences across groups with consideration of PrEP use patterns.

From a technical standpoint, transmission clusters were identified as those clades in the phylogeny including at least five individuals with viruses sharing pairwise patristic distance less than 0.02 substitutions per site in at least 90% of bootstraps support. Although the authors cite Poon et al. 2016 for such a definition, the choice of a 0.02 threshold appears to be arbitrary and unjustified. How would the conclusions change if different threshold had been used?

We have corrected this citation, for which Poon et al. 2015 was more appropriate as methodological justification for the threshold, which we have included in the methods:

**Page 6 Lines 197-201:** “Phylogenetic clusters were identified as groups of at least five individuals whose viruses share a pairwise patristic (tree) distance <0.02 substitutions per site in greater than 90% of bootstraps [24]. This pairwise tree distance threshold was identified as the 95th percentile of within-host pairwise distances for subtype B in BC [24] and is used for phylogenetic monitoring in BC.”

We agree with the reviewer that the identification of clusters will be sensitive to the patristic distance threshold, which we have added as a limitation in the Discussion. We also elaborated on further studies that have used this clustering method in BC.

**Page 19 Lines 576-591**: “Phylogenetic cluster identification is sensitive to the pairwise tree or genetic distance threshold applied, as well as other methodological considerations like tree inference or bootstrap support. Clustering methodologies can lead to differential results and sensitivities to detecting differences in transmission and sampling [53]. While HIV clusters can be alternatively identified using a pairwise genetic TN93 distance near 1.5% through HIV-TRACE [54], the patristic distance methodology has the advantage of additionally considering phylogenetic topology with an underlying evolutionary model and bootstrap support. Beyond there being an established phylogenetic monitoring program in BC based on this method, we found phylogenetic clustering identified through patristic distance was concordant with viral lineage-level diversification rate towards evaluating risk factors for elevated transmission risk [27], could be prioritized using the diversification rate [29], and delineated populations most negatively impacted by service disruption during COVID-19 [28]. A comparison of cluster membership and size distribution across methods, applying different thresholds would be worthwhile. We demonstrate heterogeneity in clusters’ transmission dynamics that can be used to more granularly simulate epidemic scenarios; how those clusters are defined will vary by setting.”
