# Supplementary material for: Heterogeneous impacts of HIV pre-exposure prophylaxis (PrEP) on drug resistance and phylogenetic cluster transmission dynamics in British Columbia, Canada: A retrospective cohort and simulation study
Source: PLoS Med. 2025 Dec 9;22(12):e1004827. doi: 10.1371/journal.pmed.1004827 (PMC12700434; doi:10.1371/journal.pmed.1004827)
Supplement: Attachment — Submitted filename: PLoSMed_editorial_responses_20251031.docx [file pmed.1004827.s008.docx]

2025-October-19

**Responses to editorial requests, PMEDICINE-D-25-01591R2**
We thank the editors and reviewers for their thorough consideration and feedback on the revised manuscript for publication in PLOS Medicine. We have addressed the editors’ requests, with our responses colored in blue text and in-text changes indented with corresponding page and line numbers in the manuscript tracked changes.

**Requests from Editors:**
** Please confirm that your title complies with PLOS Medicine's style. Your title must be nondeclarative and not a question. It should begin with main concept if possible. "Effect of" should be used only if causality can be inferred, i.e., for an RCT. Please place the study design ("A randomized controlled trial," "A retrospective study," "A modelling study," etc.) in the subtitle (ie, after a colon).

We have adjusted the title to reflect that the paper includes both a retrospective cohort study and simulation model:

**Page 1 Lines 3-5; Supp. page 1 Lines 2-4:** “Heterogeneous impacts of HIV pre-exposure prophylaxis (PrEP) on drug resistance and phylogenetic cluster transmission dynamics in British Columbia, Canada: a retrospective cohort and simulation study”

* In the last sentence of the Abstract Methods and Findings section, please describe the main limitation(s) of the study's methodology.

We have added the key limitations of the methodology described in the Discussion, Methods, and Results in the abstract.

**Page 2 Lines 47-49**: “These results must be interpreted in light of uncertainty owing to incomplete sampling, the use of consensus genomes, phylogenetic inference, and the assumptions of counterfactual simulations.”

* In the abstract, please include the important dependent variables that are adjusted for in the analyses.

This was corrected in the reporting of the Poisson model of cases averted by cluster:

**Page 2 Lines 43-47:** “Most diagnoses were averted in large and medium GBM-predominant clusters. In a Poisson model, clusters with fewer diagnoses averted were associated with having a higher median age and lower proportion of new diagnoses with PrEP use, adjusted for cluster size at the end of 2017 and proportion residing in Vancouver Coastal Health Authority.”

* Please ensure that the Introduction ends with a clear description of the study question or hypothesis.

Criterium met:

**Page 5 Lines 163-167:** “We further hypothesized that Re and diagnoses averted have been impacted heterogeneously across phylogenetic clusters since PrEP availability, based on the expectation of differential PrEP awareness, access, and retention in subpopulations of social networks represented as clusters, amid broad Re reductions in the BC GBM population.”

* Please ensure that all abbreviations are defined at first use throughout the text.
We have corrected the manuscript and supplementary to abbreviate terms at first use. Definitions in the title, abstract, author summary, citation titles, and supplementary were treated as separate, whereas captions and subtitles were considered within the main text. We assumed that HIV did not need definition. We also added the abbreviation, PrEP, to the title.

**Abbreviations checked throughout main and supp.**: ART, PrEP, NRTI, NNRTI, PI, INSTI, TDF, FTC, DDI, AZT, 3TC, ABC, AZT, D4T, CAB-LA, GBM, PWID, HCV, HET, TGM, TGW, BC, BC-CfE, DTP, TasP, RAMs/RAM, SDRM, TSM, HIRI, CD4, VL, AIDS, STI, GTR, ML, R_e_, COVID-19, FC, sd, Y, d, rt, pol, int, pro, CI, PLWH

* Please confirm that all numbers presented in the abstract are present and identical to numbers presented in the main manuscript text.
We have made sure that numbers in the abstract are identical to the main manuscript text.

* PLOS defines the “**minimal data set” to consist of the data set used to reach the conclusions drawn in the manuscript with related metadata and methods, and any additional data required to replicate the reported study findings in their entirety.** Authors do not need to submit their entire data set, or the raw data collected during an investigation. Please submit the following data:

The **values behind the means, standard deviations and other measures** reported;
The **values used to build graphs**;
The points extracted from images for analysis.

We have made a public github repository for this project [hiv_prep_bc](https://github.com/AngMcL/hiv_prep_bc) which contains de-identified and summarized data used for figures and statistical comparisons. Due to patient confidentiality and privacy, we are prohibited from sharing any potentially identifying participant data including HIV sequences or patient-level metadata.

* For studies in which a novel model is central to the manuscript's findings, authors are responsible for **providing the source code needed to replicate the study's findings in a repository (such as GitHub, SourceForge or Bitbucket)** or a cloud computing service (such as Code Ocean). Protection of authors’ intellectual property will not be cause for exception. Please explain in the manuscript’s Data Availability Statement how readers can access the shared code.
* The **current explanation for lack of code inclusion is not sufficiently justified.**
We have uploaded source code for analyses in the public github repo [hiv_prep_bc](https://github.com/AngMcL/hiv_prep_bc) and changed the wording in the methods regarding code availability:

**Page 8 Lines 321-325:** “**Data availability.** The BC-CfE is prohibited from making individuals’ viral sequences or associated metadata publicly available due to provisions in our service contracts, institutional policy, and ethical requirements. Source code, tables, and figures are available at github.com/angmcl/hiv-prep-bc.”

* Please review your manuscript and edit to ensure compliance with our **inclusive language** requirements <https://journals.plos.org/plosmedicine/s/human-subjects-research#loc-categorization>

We have ensured the language complies with inclusive language requirements, including our use of appropriate terminology for people living with HIV (PLWH), people who inject drugs (PWID), gay, bisexual, and other men who have sex with men (GBM), PLWH who have immigrated, and newly HIV diagnosed people who ever filled a prescription for HIV PrEP (i.e., PrEP users).

* Please consider avoiding the use of red and green in order to make your figure more accessible (e.g Fig. 3).

While we appreciate this commentary to make the figures accessible to those with difficulty discerning red and green, we believe that the figures are sufficiently described such that interpreting the differences between red (overall) and green (HET) is possible without distinguishing them on the plots in which they are directly compared.

* Where data points are discrete, please ensure that they are depicted in the figures as discrete data and not as a continuous line.

If the editor is referring to Figure 3B, the data points are discrete estimates of yearly changes, but they are also connected through time and I think connecting the dots makes it easier to discern trends through time.

** The funding statement should include: specific grant numbers, initials of authors who received each award, **URLs to sponsors’ websites.** Also, please **state whether any sponsors or funders (other than the named authors) played any role in study design, data collection** and analysis, the decision to publish, or preparation of the manuscript. If they had no role in the research, include this sentence: “The funders had no role in study design, data collection and analysis, decision to publish, or preparation of the manuscript.”

*Please provide the missing URLs.

The Financial Disclosure section was updated to reflect the role played by the BC-CfE and the URLs for sponsors’ websites

**Pages 21 Lines 679-692:** “AM was supported by a Canada Graduate Scholarship Doctoral award from the Canadian Institutes of Health Research (CIHR) https://cihr-irsc.gc.ca/e/193.html and the BC-CfE https://bccfe.ca/. JSGM is supported by grants paid to his institution, the BC-CfE, by the BC Ministry of Health https://www2.gov.bc.ca/gov/content/governments/organizational-structure/ministries-organizations/ministries/health, Health Canada https://www.canada.ca/en/health-canada.html, Public Health Agency of Canada (PHAC) https://www.canada.ca/en/public-health.html, Vancouver Coastal Health https://www.vch.ca/en, Vancouver General Hospital Foundation https://vghfoundation.ca/, and Genome BC https://www.genomebc.ca/. JBJ is supported by CIHR, Genome BC GeneSolve Grant #GEN054, Genome Canada https://genomecanada.ca/, PHAC, and the BC-CfE. VM, PS, JT, MH, CJB, and RB were also supported by the BC-CfE. The BC-CfE contributed to data collection, entry, storage, processing, and querying. Other funders had no role in study design, data collection and analysis, decision to publish, or preparation of the manuscript.”
** All authors must declare their relevant competing interests per the PLOS policy, which can be seen here: <https://journals.plos.org/plosmedicine/s/competing-interests> For authors with ties to industry, please indicate whether any of the interests has a financial stake in the results of the current study.
We have checked the competing interest statements and confirmed they are accurate with all co-authors.

**Page 22 Lines 703-707:** “JSGM received institutional grants from Gilead Sciences, Merck, and ViiV Healthcare, which had no financial stakes in the results of the current study. CJB has received grants and honoraria as speaker and consultant paid to his institution from Merck, Gilead Sciences, and ViiV Healthcare, which also had no stakes in the results of the study. All other authors declare no competing interests.”

Please include a completed checklist that is most appropriate for your study (e.g. STROBE, GATHER, RECORD):

* For observational studies, **please ensure that the study is reported according to the STROBE guideline, and include the completed STROBE checklist as Supporting Information.** Please add the following statement, or similar, to the Methods: "This study is reported as per the Strengthening the Reporting of Observational Studies in Epidemiology (STROBE) guideline (S1 Checklist)." The STROBE guideline can be found here: <http://www.equator-network.org/reporting-guidelines/strobe/>. When completing the checklist, please use section and paragraph numbers, rather than page numbers.

We have added a statement to the methods regarding STROBE reporting:

**Pages 7 Lines 237-239**: “This component of the study was reported as per the Strengthening the Reporting of Observational Studies in Epidemiology (STROBE) guidelines (S1 Checklist).”

We have completed and attached the STROBE guideline checklist.

* Did your study **have a prospective protocol or analysis plan**? Please state this (either way) early in the Methods section.
a) If a **prospective analysis plan (from your funding proposal, IRB or other ethics committee submission, study protocol, or other planning document written before analyzing the data) was used in designing the study, please include the relevant prospectively written document** with your revised manuscript as a Supporting Information file to be published alongside your study, and cite it in the Methods section. A legend for this file should be included at the end of your manuscript.

There was a prospective analysis plan for the initial ethics application, which we have added as a supporting information file, cited in the methods section, and mentioned in a legend of supplementary information at the end of the manuscript.

b) If no such document exists, please make sure that the Methods section transparently describes when analyses were planned, and when/why any data-driven changes to analyses took place.
c) In either case**, changes in the analysis-- including those made in response to peer review comments-- should be identified as such in the Methods section of the paper**, with rationale.

Changes in the analysis from the prospective analysis plan were added to the Methods:

**Pages 5-6 Lines 191-199**: “The final study differed from the prospective analysis plan in regards to the method applied to estimate Re, for which we initially proposed using Bayesian phylodynamic birth-death skyline models [30] and instead used EpiEstim to estimate instantaneous Re [31]. This change followed the discovery that birth-death(-sampling) models are non-identifiability in the absence of additional data [32,33]. The other change from the initial protocol was the inclusion of stochastic simulations of clusters’ growth with and without PrEP based on the observed Re dynamics and in consideration of the impact of both PrEP and COVID-19 on HIV transmission.”

* Your study is observational and therefore causality cannot be inferred. Please remove language that implies causality and refer to associations instead.

We have checked that the manuscript does not mistakenly imply causality, including:

**Page 18 Lines 532-534:** “Our analyses suggest publicly funded HIV PrEP in BC since 2018, alongside generalized access to free ART under the TasP strategy, was cumulatively associated with more than 100 averted new diagnoses […]”

**Page 18 Lines 537-540:** “While PrEP availability was associated with dampened transmission in large GBM-predominant clusters, clusters with fewer cases averted tended to have higher median age, higher proportion outside of Vancouver Coastal Health, and lower proportion GBM, illuminating prevention gaps and opportunities.”

* For all observational studies, in the manuscript text, please indicate:

(1) the specific hypotheses you intended to test,

We have stated our hypotheses in the introduction:

**Page 5 Lines 158-167:** “We tested the hypotheses that newly HIV diagnosed PrEP users were more likely to be included in phylogenetic clusters in BC than newly diagnosed non-PrEP users generally and among GBM, under the expectation that PrEP eligibility and use is related to behaviour and linkage to sexual transmission networks, and that this results in an elevated likelihood of baseline NRTI resistance for newly HIV diagnosed with previous PrEP use. We further hypothesized that Re and diagnoses averted have been heterogeneous across phylogenetic clusters since PrEP availability, based on the expectation of differential PrEP awareness, access, and retention in subpopulations of social networks represented as clusters, amid broad Re reductions in the BC GBM population.”

(2) the analytical methods by which you planned to test them,

Please see Methods **Pages 5-8 Lines 169-325.**

(3) the analyses you actually performed, and

The analyses performed were described in the Results, **Pages 8-18 Lines 327-529.**

(4) when reported analyses differ from those that were planned, transparent explanations for differences that affect the reliability of the study's results. If a reported analysis was performed based on an interesting but unanticipated pattern in the data, please be clear that the analysis was data-driven.
We have clarified where the reported analyses differ from what was planned:

**Pages 5-6 Lines 191-199**: “The final study differed from the prospective analysis plan in regards to the method applied to estimate Re, for which we initially proposed using Bayesian phylodynamic birth-death skyline models [30] and instead used EpiEstim to estimate instantaneous Re [31]. This change followed the discovery that birth-death(-sampling) models are non-identifiability in the absence of additional data [32,33]. The other change from the initial protocol was the inclusion of stochastic simulations of clusters’ growth with and without PrEP based on the observed Re dynamics and in consideration of the impact of both PrEP and COVID-19 on HIV transmission.”

***For a population health/registry study, please report that data according to the RECORD guideline (available from** [**https://www.record-statement.org**](https://www.record-statement.org/)**) and include the completed checklist as Supporting Information.** Please add the following statement, or similar, to the Methods: "This study is reported as per the Reporting of Studies Conducted using Observational Routinely-Collected Data (RECORD) guideline (S1 Checklist)." When completing the checklist, please use section and paragraph numbers, rather than page numbers
N/A

*** For population-level health estimates, we as that you report your data according to GATHER and enclose a completed GATHER checklist as a supplementary document.** See <http://gather-statement.org/> In the checklist please include sufficient text excerpted from the manuscript to explain how you accomplished all applicable items.

N/A

For the simulations of cluster growth, please consider whether these guidelines for modeling should be adopted (derived from Geoffrey P Garnett, Simon Cousens, Timothy B Hallett, Richard Steketee, Neff Walker. Mathematical models in the evaluation of health programmes. (2011) Lancet DOI:10.1016/S0140-6736(10)61505-X):

Thank you for the recommendation. We have reviewed Garnett et al. 2011, cited this paper, and reported how we followed those recommendations, including through reporting of simulation parameters specified, observed, and estimated in Table S7 and ranges of uncertainty evaluated, as well as a flow diagram (Figure 5).

**Page 8 Lines 289-291**: “The simulation parameters specified and estimated were reported with ranges of uncertainty (Table S7) in accordance with guidelines for reporting simulations and mathematical models [53].”

* Please provide a diagram that shows the model structure, including how the disease natural history is represented, the process and determinants of disease acquisition, and how the putative intervention could affect the system.

We have provided a flow diagram (Figure 5) to clarify the model structure and how the intervention could differentially impact cluster growth. **Page 16 Lines 487-491:**

**Figure 5. Flow diagram of stochastic simulations fit to observed scenario with PrEP and for counterfactual scenario without PrEP.** Orange circles were observed parameters, blue were parameters specified based on the literature or PrEP effect, and green were estimated parameters (Table S7).

* Please provide a complete list of model parameters, including clear and precise descriptions of the meaning of each parameter, together with the values or ranges for each, with justification or the primary source cited, and important caveats about the use of these values noted.

We have added a complete list of model parameters, ranges, and citations where applicable in the newly added, **Table S7**.

**Table S7**. **Parameters of stochastic branching process simulations were specified based on the literature, observed or estimated at the cluster-level.** Parameters were grouped into types by whether they were specified based on literature, observed (trends) at cluster-level, estimated at cluster-level in simulations. Standard deviation (sd). Uncertainty in R_e_ estimation was explored for variables marked **.

| Type | Parameter | Range | Specified or output | References |
| --- | --- | --- | --- | --- |
| Specified based on literature | Dispersion k | **k=0.1-0.3 | Specified in simulations: UNIF(k) | Althaus 2015; Riou & Althaus 2020; Lloyd-Smith et al. 2005 |
|  | Serial interval | **Mean=0.5-5 y | Specified to estimate Re and in simulations: Gamma(mean, std) | Hollingsworth et al. 2008, 2015; Peruski et al. 2021 |
|  |  | **sd=0.5-2 y |  |  |
|  | P(diagnosed\|case) | 82% | Specified to estimate initial seed size, convert estimated N cases to N diagnoses | Lima et al. 2017 |
|  | P(ART\|diagnosed) | 76% | Specified to estimate initial seed size |  |
|  | P(virally suppressed\|ART) | 83% | Specified to estimate initial seed size |  |
| Observed, cluster-level | Instantaneous effective reproduction number (Re) | **Estimating interval (0.25, 0.5, 1 y) | Specified for simulations:  ~ NegBin(mean=Re, disp=k);  Period ave. Re (pre/during PrEP;pre/during/post COVID-19) used to calculate PrEP effect | Cori et al. 2013; Fig. S24-S28, S30 |
|  |  | **Serial interval ~ Gamma(mean 0.5-5 y, sd 0.5-2 y) |  |  |
|  |  |  |  |  |
|  |  | **Smoothing interval (30 d, 90 d, 365 d) |  |  |
|  | Observed N diagnoses, 2018-2022 | Summarized as rolling average, cumulative | Specified to estimate seed adjust factor and cases averted | Table S6 |
| Observed trend, cluster-level | PrEP effect (fold-change Re) | Average Re with PrEP pre-COVID / average Re before PrEP | Specified to adjust Re without PrEP | Fig. S29 |
|  | Adjusted Re without PrEP | Adj Re = Observed Re / PrEP effect | Used in counterfactual simulations | Fig. S30 |
| Estimated, cluster-level | Initial seed size (n infectious at time zero, Jan 1 2018) | (N cases 2018 – N migrants – N deaths) * (P(not diagnosed\|case) + P(no ART\|diagnosed) + P(not virally suppressed\|ART) ) | Used in initial simulation | Table S6 |
|  | Initial simulated N new cases and diagnoses, 2018-2022 | Rolling average, cumulative for each cluster and overall for Scenarios 1 and 2 | Simulation output | Table S6; Fig. S31 |
|  | Seed adjustment factor | Observed N diagnoses / Initial simulated N diagnoses | Simulation output | Table S6 |
|  | Adjusted seed size | Initial seed size * seed adjust factor | Simulation output;  Used in subsequent simulation | Table S6 |
|  |  |  |  |  |
|  | Simulated N new cases and diagnoses, 2018-2022 | Rolling average, cumulative for each cluster and overall for Scenarios 1 and 2 | Simulation output | Fig. S32, S33 |
|  | Diagnosed cases averted | N diagnoses simulated without PrEP - N diagnoses observed with PrEP | Simulation output | Fig. 6 |

We have discussed parameters’ caveats:

**Pages 20 Lines 621-651:** “Our estimations of averted infections are sensitive to clusters’ Re, with its underlying uncertainty, smoothing, and serial interval, as well as dispersion and the seed size. Cluster Re confidence intervals were wide when cases were sparse (Fig. 4, Fig. S24), especially for small and medium-sized clusters. We addressed this by comparing estimation and smoothing intervals for Re to maximize signal and reduce noise, and screening for wide confidence intervals. Further reducing noise in time-series data could improve Re estimates for low incidence [63]. Simulations could be impacted by inaccurate Re due to noise or delayed diagnoses, as well as by inaccurate initial seeds, justifying the calibration of the initial number of infectious individuals for each cluster to yield a similar number of new cases as observed. The specification of observed cluster Re partially controls for co-occurring epochal effects in simulations. For instance, the COVID-19 pandemic led to reduced access to treatment and prevention resources like safe injection sites, resulting in elevated growth of some PWID-predominant HIV clusters during COVID-19 [28]. The effects of COVID-19 were excluded from estimating the effect of PrEP on cluster transmission by estimating the fold-change of average Re in the period preceding PrEP to the period with PrEP and before COVID-19 interventions (January 2018 to January 2020). Stochastic simulations also incorporated uncertainty through the number of secondary infections, as drawn from a negative binomial distribution defined by a mean of Re and dispersion parameter k. It is possible that while accommodating infrequent superspreading, we could have overestimated the number of secondary infections in some cluster simulations; this is why the seed size and hyperparameters were optimized to minimize the difference between observed cases with PrEP compared to simulated in the scenario with PrEP. Re estimates depend on the serial interval, i.e., the time between successive infections’ symptom onset [64], which for HIV can vary widely as transmission could occur during acute infection (45% of transmissions in the first year in a Detroit MSM cohort) [65] or later if an individual has viral rebound (to a detectable viral load more than 200 copies/mL) due to treatment interruption or emergence of drug resistance. We compared serial intervals with a mean from 0.5 to five years, reporting one year for the primary analysis under the premise testing and treatment in BC are widely available. Although temporal trends in Re were robust to serial interval specifications, Re estimates carry uncertainty forward to estimates of cases averted.”

* Please provide a clear statement about how the model was fitted to the data including where relevant goodness-of-fit measure, the numerical algorithm used, which parameter varied, constraints imposed on parameter values, and starting conditions.

The addition of Table S7 satisfies this criterium, in addition to the description of methodologies to adjust the seed to minimize the difference between observed new diagnoses in clusters from 2018-2022 and simulated new diagnoses from 2018-2022 based on observed Re (with PrEP):

* For uncertainty analyses, please state the sources of uncertainties quantified and not quantified this can include parameter, data, and model structure.

We have summarized parameters in the simulation, and uncertainty ranges that were evaluated in **Table S7.**

* Please provide sensitivity analyses to identify which parameter values are most important in the model. Uncertainty estimates seek to derive a range of credible results on the basis of an exploration of the range of reasonable parameter values. The choice of method should be presented and justified.

We appreciate the recommendation to add sensitivity analyses for the simulation model, for which we could modify any of the specified parameters (Table S6) including the proportion diagnosed of infected, proportion on ART of diagnosed, proportion virally suppressed of those on ART, serial interval hyperparameters, R_e_ hyperparameters, k, or the periods over which R_e_ was averaged in order to estimate the PrEP effect to see. However, evaluating the behaviour of stochastic branching processes in light of relative changes in the underlying parameters beyond a primary set of parameters informed through empirical observations is a study in and of itself and seems out of scope for this manuscript, which serves to evaluate heterogeneity in clusters’ transmission dynamics based on PrEP availability. We have considered uncertainty in several parameters (Table S6) including the serial interval mean and standard deviation (sd), for Re, the estimating interval, serial interval mean and sd, and the smoothing interval. We acknowledge in the limitations that there are multiple sources of uncertainty in the model, to which it is challenging to ascribe the relative impacts.

* Please discuss the scientific rationale for this choice of model structure and identify points where this choice could influence conclusions drawn. Please also describe the strength of the scientific basis underlying the key model assumptions.
Rationale for stochastic branching processes for simulations:

**Page 7 Lines 275-276:** “To reflect infrequent superspreading and variable generation times, stochastic branching processes were used [..]”

Rationale for serial intervals:

**Page 7 Lines 262-265:** “These serial interval ranges reflect that a substantial proportion of transmission occurs during early infection due to a high viral load, with infrequent longer intervals for transmission from chronic infections [46,47]. Further, 36% of HIV infections in the US from 2004 to 2008 were diagnosed within 1 y [48].”

Discussion of limitations of simulation model and parameters:

**Page 20 Lines 621-651:** “Our estimations of averted infections are sensitive to clusters’ R_e_, with its underlying uncertainty, smoothing, and serial interval, as well as dispersion and the seed size. Cluster R_e_ confidence intervals were wide when cases were sparse (Fig. 4, Fig. S24), especially for small and medium-sized clusters. We addressed this by comparing estimation and smoothing intervals for R_e_ to maximize signal and reduce noise, and screening for wide confidence intervals. Further reducing noise in time-series data could improve R_e_ estimates for low incidence [63]. Simulations could be impacted by inaccurate R_e_ due to noise or delayed diagnoses, as well as by inaccurate initial seeds, justifying the calibration of the initial number of infectious individuals for each cluster to yield a similar number of new cases as observed. The specification of observed cluster R_e_ partially controls for co-occurring epochal effects in simulations. For instance, the COVID-19 pandemic led to reduced access to treatment and prevention resources like safe injection sites, resulting in elevated growth of some PWID-predominant HIV clusters during COVID-19 [28]. The effects of COVID-19 were excluded from estimating the effect of PrEP on cluster transmission by estimating the fold-change of average R_e_ in the period preceding PrEP to the period with PrEP and before COVID-19 interventions (January 2018 to January 2020). Stochastic simulations also incorporated uncertainty through the number of secondary infections, as drawn from a negative binomial distribution defined by a mean of R_e_ and dispersion parameter k. It is possible that while accommodating infrequent superspreading, we could have overestimated the number of secondary infections in some cluster simulations; this is why the seed size and hyperparameters were optimized to minimize the difference between observed cases with PrEP compared to simulated in the scenario with PrEP. R_e_ estimates depend on the serial interval, i.e., the time between successive infections’ symptom onset [64], which for HIV can vary widely as transmission could occur during acute infection (45% of transmissions in the first year in a Detroit MSM cohort) [65] or later if an individual has viral rebound (to a detectable viral load more than 200 copies/mL) due to treatment interruption or emergence of drug resistance. We compared serial intervals with a mean from 0.5 to five years, reporting one year for the primary analysis under the premise testing and treatment in BC are widely available. Although temporal trends in R_e_ were robust to serial interval specifications, R_e_ estimates carry uncertainty forward to estimates of cases averted.”

**Text edits:**
Use the active voice throughout.

We have taken this into consideration throughout the manuscript, including

**Abstract Page 1 Lines 41-43:** “We applied simulations of active clusters’ growth based on their empirically observed Re with or without estimated PrEP impacts to estimate diagnoses averted via PrEP across clusters, with non-clustered cases grouped together.”

Line 18: replace ‘taken’ with ‘used’

Corrected.

Line 23: add ‘against HIV acquisition after ‘effectiveness’

Corrected.

Please revise and shorten sentence line 24-28. ‘including heterogeneity’ is not clear. ‘propose key groups..’ seems adjacent to the main findings.

Changed:

**Page 1 Lines 25-28:** “We evaluated individual and population-level impacts of PrEP on HIV drug resistance and transmission in phylogenetic clusters, representing groups of individuals linked by recent outbreaks, to elucidate heterogeneity in its effectiveness.”

In the Methods and Findings: please state the size and source of the cohort and clarify that you are using sequence data to identify phylogenetic clusters and baseline resistance mutations.

Clarified in the abstract:

**Page 2 Lines 32-33**: “(i.e., PrEP users) in BC (n=39) compared to non-PrEP users (n=566) diagnosed from 2018 to 2022 in the BC Drug Treatment Program with at least one sequence available.”

And in the Methods:

**Page 5 Lines 180-182**: “The retrospective cohort of newly diagnosed PrEP users and non-PrEP users included a subset of DTP participants described below.”

**Page 6 Lines 229-236**: “To evaluate individual-level impacts of PrEP on baseline drug resistance and clustering, we applied a retrospective cohort study design to compare newly HIV diagnosed previous PrEP users (n=39) with date of first detectable VL from Oct. 23, 2018 to Dec. 5, 2022, with a control group of newly diagnosed non-PrEP users (n=566) over the same period. Cohort eligibility included DTP participants with at least one sequence available and a first detectable VL from Oct. 23, 2018 to Dec. 5, 2022, excluding those with previous ART experience (PLWH who have immigrated). Individuals enrolled for PrEP who never filled prescriptions were considered non-PrEP users.”

Line 31: please qualify that newly diagnosed is with HIV.

Thank you for noting this omission. Corrected here and elsewhere in the manuscript, at least in the first sentence of each paragraph mentioning ‘newly diagnosed’.

Lines 29-41 can be slightly abbreviated, but specific results need to be added. For example, revise to: “Using a retrospective cohort design, we  evaluated the frequencies of baseline drug resistance mutations and membership in phylogenetic clusters among newly diagnosed PrEP users in BC (n=39) compared to non-PrEP users (n=566) diagnosed from 2018 to 2022. Newly HIV-diagnosed persons using PrEP were significantly more likely **[add 95% CI]** than newly diagnosed non-PrEP  users to have/carry viruses that formed part of phylogenetic clusters and contained the baseline nucleoside analogue reverse transcriptase inhibitor (NRTI) resistance mutation M184I/V”.

We have incorporated your suggestions and added clarification of the terminology ‘PrEP user’, added statistical tests’ p-values:

**Page 1 Lines 29-37:** “Using a retrospective cohort design, we evaluated the frequencies of baseline drug resistance mutations and membership in phylogenetic clusters among newly HIV diagnosed people who ever filled a prescription for HIV PrEP (i.e., PrEP users) in BC (n=39) compared to non-PrEP users (n=566) diagnosed from 2018 to 2022 in the BC Drug Treatment Program with at least one sequence available. Newly HIV diagnosed PrEP users were significantly more likely than newly diagnosed non-PrEP users to be included in phylogenetic clusters (chi-squared test, p=0.0075) and carry baseline nucleoside analogue reverse transcriptase inhibitor (NRTI) resistance mutation M184I/V (Fisher’s exact test, adjusted p-value=0.025).”

*Can PrEP ‘users’ be revised to more inclusive language?

We appreciate the need to use terminology that is inclusive of individuals’ different experiences with PrEP. We opted to keep the brief terminology PrEP user in the majority of the text as it is used profusely (72 times in fact). We defined the term upon first usage in the abstract, summary, and introduction:

**Page 2 Lines 31-33; Page 3 Lines 69-70; Page 4 Lines 121-122:** “newly HIV diagnosed people who ever filled a prescription for HIV PrEP (i.e., PrEP users)”.

*Please don’t add conclusions to the Methods and Findings section of the Abstract, and remove causal language. E.g. “Most diagnoses were averted in large…” As this is a modeling study, causal language should be removed and associations described.

We appreciate the clarification needed and have changed the language where causative associations were exaggerated beyond limitations.

However, in the case of “Most diagnoses were averted in large…”, this was not a model finding, but a direct observation of the clusters we found had the most cases averted (Fig. 5). We have clarified this direct observation from the statement regarding factors associated with clusters having fewer cases averted based on the adjusted Poisson model.

**Page 2 Lines 43-47:** “Most diagnoses were averted in large and medium GBM-predominant clusters. In a Poisson model of diagnoses averted within clusters, clusters with fewer diagnoses on average were associated with having a higher median age and lower proportion of new diagnoses with PrEP use, adjusted for cluster size at the end of 2017 and proportion residing in Vancouver Coastal Health Authority.”

Lines 42-43: this clause “and fewer diagnoses were averted in clusters with a higher median age, lower proportion of new diagnoses with PrEP use” is not clear to the general reader.

We have clarified this sentence:

**Abstract Page 2 Lines 44-48:** “In a Poisson model, clusters with fewer diagnoses averted were associated with having a higher median age and lower proportion of new diagnoses with PrEP use, adjusted for cluster size at the end of 2017 and proportion residing in Vancouver Coastal Health Authority.”

**Summary Page 3 Lines 83-84:** “In a Poisson model, fewer cases were averted in clusters comprised of older individuals with infrequent previous PrEP use.”

Please add limitations of the methodology as the last sentence of the Methods and Findings section.
We have added a limitations statement:

**Page 2 Lines 47-49:** “These results must be interpreted in light of uncertainty owing to incomplete sampling, the use of consensus genomes, phylogenetic inference, and the assumptions of counterfactual simulations.”

Line 50: “and 50 pinpoint groups for prioritized PrEP services.” Please temper ‘pinpoint’ Please also note that the heterogeneity mentioned is not clear in the Methods and Findings subsection.

We have re-worded:

**Page 2 Line 55**: ‘pinpoint groups’ to ‘suggest groups’

Description of cluster R_e_ heterogeneity in Results:

**Page 14 Lines 455-468**: “Transmission within and outside BC HIV phylogenetic clusters was variably impacted by PrEP (Fig. 4, Fig. S24-30). Cluster 31, the largest GBM-predominant cluster (size=391, 89% GBM, 94 new cases since 2018), had stable R_e_ near 1, followed by an increase in mid-2017, then a decline since PrEP availability (fold-change R_e_ with PrEP<1). Other GBM-predominant clusters (cluster 95, size=158, 27 new cases; and cluster 22, size=42, 19 new cases) had sustained declines through PrEP, with recent resurgences post-COVID-19 (Fig. S29). The two largest PWID-predominant clusters (clusters 49 and 57, sizes 459 and 471, 91% and 86% PWID) have had limited new cases since 2018 (11 and 19). R_e_ of PWID cluster 57 was stably below or near 1 from 2015 to 2018, then rose significantly above 1 in 2019, coming down in 2020 before rising above 1 post-COVID-19. PWID cluster 137 (size=90, 26 new cases, 88% PWID) has also had volatile R_e_, peaking in 2017-2018, dropping in 2019, and rising again in late 2019 and 2020. Comparing the PrEP effect on cluster-level R_e_ in key populations, 50% of GBM clusters had reduced R_e_ following PrEP, compared to 33.3% of PWID clusters (Fig. 4).”

Lines 74-78: please break in 2 for clarity. However please note that all of the results do not need to be reiterated in the Author Summary.

We have broken up the author summary and made it more concise:

- **Page 3 Lines 80-84:** “Across clusters, we estimate that PrEP averted about 20 new HIV infections annually from 2018 to 2022, but it was most effective in reducing R_e_ and averting new cases in medium and large gay, bisexual, and other men who have sex with men (GBM) clusters. In a Poisson model, fewer cases were averted in clusters comprised of older individuals with infrequent previous PrEP use.”

Please also note that the Conclusions section does not explain what the phylogenetic clustering might mean. Overall the Abstract needs to better communicate what the key findings are in this report and what it adds to the literature.

We reworded for clarity:

**Abstract Background Page 2 Lines 27-28:** “phylogenetic clusters, representing groups of individuals linked by recent outbreaks,”

Line 122: THE is capitalized. Please correct.

Corrected.

Line 151 and elsewhere: suggest to delete ‘join’ and replace with ‘form part of’, given that ‘join’ is active and seems inappropriate in this instance.

We changed ‘joined/join’ to ‘were included in’ throughout.

For clarity: is preventing 20 new HIV diagnoses a per cluster estimate? If so, please rephrase.

We re-worded for clarity:

**Abstract Page 2 Lines 50-52:** “We estimated that the oral PrEP program in BC from 2018 to 2022 averted approximately 20 new HIV diagnoses per year across phylogenetic clusters”

**Summary Page 3 Lines 81-82:** “Across clusters, we estimate that PrEP averted about 20 new HIV infections annually from 2018 to 2022,”

Line 345: please rephrase ‘a clinical miss’

Changed to “diagnostic false negative” and “false negative”.

For all confidence intervals, please use a comma rather than a hyphen.

Corrected.

Please ensure that no new results are reported in the Discussion and that all findings are discussed in the Results section.

We have checked that there are no new results in the Discussion.

Please ensure that the power calculation is discussed in the Methods.

We have added the power calculation in the methods:

**Page 7 Lines 251:** “statistical power was calculated for the M184IV Fisher’s test using alpha 0.05.”

Line 493: where does the number ‘100’ come from? Here it would be helpful to understand the% of projected at risk persons accessing PrEP.

We have clarified where this number came from, and how to interpret this across persons at risk who accessed PrEP over the study period:

**Page 18 Lines 532-534:** “was cumulatively associated with more than 100 averted new diagnoses from 2018 to the end of 2022 across active phylogenetic clusters and non-clustered cases”

We have reported cumulative PrEP participants at risk of seroconverting in the Introduction:

**Page 4 Lines 114-125:** “PrEP uptake in BC has expanded rapidly, with 9737 cumulative participants by June 30, 2022 [7,11], consisting of 97.0% cis GBM, 1.3% TGW, 0.9% cis-women, and 0.5% transgender men (TGM) [7]. […] Lapsed prescriptions (greater than six months beyond expected refill date based on daily PrEP use) and incomplete adherence have resulted in 39 newly HIV diagnosed people who ever filled a prescription for HIV PrEP (i.e., PrEP users) between October 23, 2018 and December 5, 2022 (0.4% of PrEP participants).”

The Discussion exceeds 200 words. Please remove repetition of results and streamline. Focus on the novel findings that might inform other studies and ensure the Discussion is interpretable by a general reader. Please do not repeat information in the Introduction and limit recapitulation of the results. Please communicate what is of most interest to public health efforts and interventions.

We have reduced the text in the discussion to focus on topics of utmost interest to public health efforts and interventions, although 200 words was not possible given this research has implications for drug resistance surveillance, clinical outcomes, public health programs, as well as phylogenetic, mathematical, and health economics modelling.

**Comments from Reviewers:**

Reviewer #1: The authors answered all of my questions and satisfactory addressed my concerns. Where additional analyses were not possible, I understand the justifications.

Reviewer #2: the authors have satisfactorily addressed my comments, I do not have further revisions to suggest.

Reviewer #3: I must compliment the authors for the excellent job done in revising the manuscript. All concerns from the reviewers were, in my opinion, satisfactorily addressed. In particular, I commend the authors for clarifying and discussing the potential limitations of their work. This is now an original and well written manuscript.

Thank you very much to the reviewers and editors for their time in evaluating and improving this manuscript.

Best regards,

Angela McLaughlin,

Jeffrey Joy, et al.
